# A VgrG2b fragment cleaved by caspase-11/4 promotes *Pseudomonas aeruginosa* infection through suppressing the NLRP3 inflammasome

**DOI:** 10.7554/eLife.99939

**Published:** 2025-02-25

**Authors:** Yan Qian, Qiannv Liu, Xiangyun Cheng, Chunlei Wang, Chun Kong, Mengqian Li, Chao Ren, Dong Jiang, Shuo Wang, Pengyan Xia

**Affiliations:** 1 Department of Immunology, School of Basic Medical Sciences, Peking University Beijing China; 2 NHC Key Laboratory of Medical Immunology, Peking University Beijing China; 3 Key Laboratory of Molecular Immunology, Chinese Academy of Medical Sciences Beijing China; 4 Department of Sports Medicine, Peking University Third Hospital Beijing China; 5 Beijing Key Laboratory of Sports Injuries, Institute of Sports Medicine of Peking University Beijing China; 6 Department of Respiratory and Critical Care Medicine, Beijing Institute of Respiratory Medicine, Beijing Chao-Yang Hospital, Capital Medical University Beijing China; 7 CAS Key Laboratory of Pathogen Microbiology and Immunology, Institute of Microbiology, Chinese Academy of Sciences Beijing China; Yale University United States; Yale University United States

**Keywords:** *P. aeruginosa*, non-canonical inflammasome, caspase-11/4, VgrG2b, NLRP3, Human, Mouse

## Abstract

The T6SS of *Pseudomonas aeruginosa* plays an essential role in the establishment of chronic infections. Inflammasome-mediated inflammatory cytokines are crucial for host defense against bacterial infections. We found that *P. aeruginosa* infection activates the non-canonical inflammasome in macrophages, yet it inhibits the downstream activation of the NLRP3 inflammasome. The VgrG2b of *P. aeruginosa* is recognized and cleaved by caspase-11, generating a free C-terminal fragment. The VgrG2b C-terminus can bind to NLRP3, inhibiting the activation of the NLRP3 inflammasome by rejecting NEK7 binding to NLRP3. Administration of a specific peptide that inhibits caspase-11 cleavage of VgrG2b significantly improves mouse survival during infection. Our discovery elucidates a mechanism by which *P. aeruginosa* inhibits host immune response, providing a new approach for the future clinical treatment of *P. aeruginosa* infections.

## Introduction

The non-canonical inflammasomes, consisting of murine caspase-11 or human caspase-4/5, recognize bacterial lipopolysaccharide (LPS) in the cytoplasm (Hagar et al., 2013; Kayagaki et al., 2013; Shi et al., 2014). Once activated, non-canonical inflammasomes induce the maturation of gasdermin D (GSDMD) to form pores on cell and mitochondrial membranes (Huang et al., 2020; Kayagaki et al., 2015; Shi et al., 2015). It also activates the NOD-, LRR-, and pyrin domain-containing protein 3 (NLRP3) inflammasome (Kayagaki et al., 2015; Rathinam et al., 2019). NLRP3 is a key intracellular receptor that responds to a range of endogenous and exogenous stimuli (Barnett et al., 2023; Christgen et al., 2020). NLRP3 recruits NIMA-related kinase 7 (NEK7) and undergoes polymerization, inducing the activation of caspase-1 and maturation of interleukin-1β (IL-1β) (Barnett et al., 2023; He et al., 2016). Non-canonical inflammasomes promote the assembly and final activation of the NLRP3 inflammasome through activating the nuclear orphan receptor NUR77 with the help of LPS and mitochondrial dsDNA (Zhu et al., 2023).

*Pseudomonas aeruginosa* is an opportunistic pathogenic bacterium widely distributed in the environment, which can cause acute and chronic infections in humans (Cendra and Torrents, 2021; Rossi et al., 2021). *P. aeruginosa* employs multiple secretion systems to release virulence factors into the extracellular environment or host cells, facilitating its survival and infection (Rossi et al., 2021). Among them, the type III secretion system (T3SS) and the type VI secretion system (T6SS) are two important secretion systems that play important roles in acute and chronic infections of *P. aeruginosa*, respectively (Faure et al., 2014; Goodman et al., 2004). T3SS can directly inject effector proteins into target cells to alter their function or cause target cell death (Hauser, 2009; Qin et al., 2022). *P. aeruginosa* encodes three different T6SSs, namely Hcp secretion island-I (H1)-T6SS, H2-T6SS, and H3-T6SS (Hachani et al., 2011; Mougous et al., 2006). These T6SSs play a crucial role in biofilm formation, competition between bacteria, and toxicity to host cells (Chen et al., 2015; Ho et al., 2014). H1-T6SS only targets bacteria and is used for competition between bacteria (Hood et al., 2010). H2-T6SS and H3-T6SS are involved in interactions between bacteria and host cells (Jiang et al., 2014; Russell et al., 2013).

The effector proteins of H2-T6SS can target both bacteria and eukaryotic cells (Ho et al., 2014; Jiang et al., 2014). T6SS is composed of structural proteins and virulence factors (Durand et al., 2014; Ho et al., 2014). Structural proteins such as hemolysin-coregulated protein (Hcp), valine–glycine repeat protein G (VgrG), and proline–alanine–alanine–arginine repeats (PAAR) form a stable spike-like structure (Leiman et al., 2009; Shneider et al., 2013). Toxic factors are transported to the extracellular space through T6SS, thereby exerting toxic effects on other bacteria or host cells (Ho et al., 2014; Qin et al., 2022). VgrG2b is an important component of T6SS, which usually binds tightly to other components of T6SS such as the Hcp protein, together forming the core component of the secretion system (Sana et al., 2015). The VgrG2b protein has a special structure for recognizing and binding effector proteins, which are transported to the tip of the secretion system and injected into the target cells (Durand et al., 2014; Sana et al., 2015). VgrG2b, upon entering eukaryotic cells, can also modulate host cell biology by interacting with cytoskeletal proteins (Sana et al., 2015; Wood et al., 2019).

Here, we found that *P. aeruginosa* infection activates caspase-11 but inhibits the activation of NLRP3 inflammasomes in macrophages. The VgrG2b entering the host cell is recognized and cleaved by caspase-11 to generate a free C-terminal fragment. This fragment can bind to NLRP3 and inhibit its binding to NEK7, thereby preventing the activation of the NLRP3 inflammasome. This inhibitory effect by *P. aeruginosa* effectively prevents further host immune activation and plays a crucial role in establishing bacterial infection. Our findings may provide a new perspective for the treatment of *P. aeruginosa* infections in the future.

## Results

### *P. aeruginosa* causes abnormal signaling of non-canonical inflammasome

**Figure 1.**
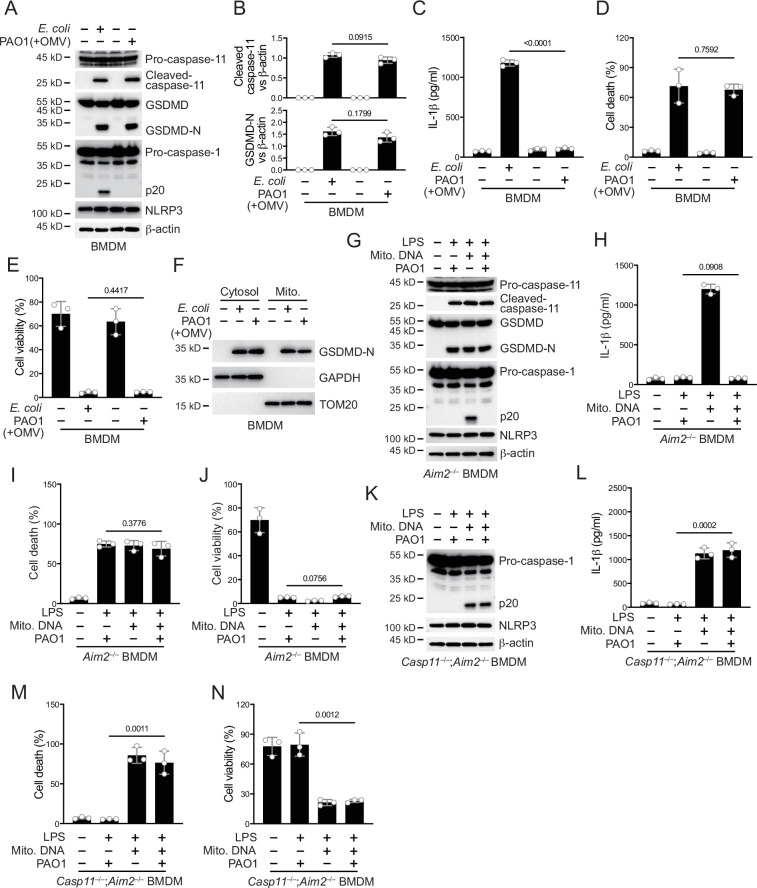
*P. aeruginosa* infection suppresses NLRP3 inflammasome. (**A–E**) Wild-type BMDM cells were primed overnight with 1 μg/ml Pam3CSK4 and incubated with *E. coli* or *P. aeruginosa* (ΔRetS PAO1) at a multiplicity of infection (MOI) of 30 and 20 μg/ml outer membrane vesicles (OMVs) for 2 hr. Cells were then supplemented with fresh medium containing 100 μg/ml Gentamycin. Cells were lysed and immunoblotted as indicated 16 hr post infection (**A**). Band intensities of cleaved caspase-11 (top) and GSDMD (bottom) were quantified and compared to that of β-actin (**B**). Cell culture supernatants were collected for an ELISA assay to determine the secreted IL-1β protein levels 16 hr post infection (**C**). Cytotoxicity was determined by lactate dehydrogenase (LDH) release assay in cell culture supernatants 16 hr post infection (**D**). Cell viability was determined by an ATP quantification assay in cell pellets 16 hr post infection (**E**). (**F**) Wild-type BMDM cells were primed overnight with 1 μg/ml Pam3CSK4 and incubated with *E. coli* or ΔRetS PAO1 at an MOI of 30 and 20 μg/ml OMVs for 2 hr. Cells were then supplemented with fresh medium containing 100 μg/ml Gentamycin. Cells were subjected to cellular component fractionation and immunoblotted as indicated 16 hr post infection. (**G–J**) *Aim2*^–/–^ BMDM cells were primed overnight with 1 μg/ml Pam3CSK4, followed by transfection of 2 μg/ml lipopolysaccharide (LPS) and 2 μg/ml extracted BMDM mitochondrial DNA using DOTAP with or without the incubation of ΔRetS PAO1 at an MOI of 30 for 2 hr. Cells were then supplemented with fresh medium containing 100 μg/ml Gentamycin. Cells were lysed and immunoblotted 16 hr post infection (**G**). Cell culture supernatants were collected for an ELISA assay to determine the secreted IL-1β protein levels 16 hr post infection (**H**). Cytotoxicity was determined by LDH release assay in cell culture supernatants 16 hr post infection (**I**). Cell viability was determined by an ATP quantification assay in cell pellets 16 hr post infection (**J**). (**K–N**) *Casp11*^–/–^;*Aim2*^–/–^ BMDM cells were primed overnight with 1 μg/ml Pam3CSK4, followed by transfection of 2 μg/ml LPS and 2 μg/ml extracted BMDM mitochondrial DNA using DOTAP with or without the incubation of ΔRetS PAO1 at an MOI of 30 for 2 hr. Cells were then supplemented with fresh medium containing 100 μg/ml Gentamycin. Cells were lysed and immunoblotted 16 hr post infection (**K**). Cell culture supernatants were collected for an ELISA assay to determine the secreted IL-1β protein levels 16 hr post infection (**L**). Cytotoxicity was determined by LDH release assay in cell culture supernatants 16 hr post infection (**M**). Cell viability was determined by an ATP quantification assay in cell pellets 16 hr post infection (**N**). Data were shown as means ± SD. For C–E, H–J, L–N, data of three independent experiments were calculated. Experiments were repeated three times with similar results. Figure 1—source data 1.File containing labeled original western blots for Figure 1. Figure 1—source data 2.Original gel image files for western blot analysis displayed in Figure 1. Figure 1—source data 3.Original source data for graphs displayed in Figure 1, Figure 1—figure supplement 1.

Non-canonical inflammasomes are activated during the infection of Gram-negative bacteria such as *P. aeruginosa* in vivo (Balakrishnan et al., 2018; Kayagaki et al., 2013; Rathinam et al., 2019). Obstruction of the inflammasome pathway can lead to chronic infections (Huus et al., 2016; Phuong et al., 2021). To investigate whether T6SS of *P. aeruginosa* modulated the non-canonical inflammasome pathway to establish chronic infections, we used an *RetS* mutant strain that specifically expresses T6SS (Allsopp et al., 2017; Goodman et al., 2004; Han et al., 2019). As an extracellular bacterial, *P. aeruginosa* synthesizes outer membrane vesicles (OMVs) to induce non-canonical inflammasome activation (Deo et al., 2020). We therefore added *P. aeruginosa* and OMVs to macrophages in vitro to simulate the real situation of infection in vivo as much as possible (Esoda and Kuehn, 2019). We found that activation of caspase-11 and cleavage of GSDMD in bone marrow-derived macrophage (BMDM) cells were not affected after incubation with *P. aeruginosa* and OMVs (Figure 1A, B). However, activation of caspase-1 was significantly inhibited in cells infected with *P. aeruginosa* (Figure 1A). Consistent with the results of immunoblotting, the secretion of IL-1β was diminished after infection with *P. aeruginosa* (Figure 1C). Although the mortality rate of *P. aeruginosa*-infected cells was increased (Figure 1D) and the survival rate was decreased (Figure 1E), there was not much difference compared to cells stimulated with intracellular *E. coli*. These results indicate that the activation of NLRP3 inflammasomes post non-canonical inflammasome stimulation is inhibited in the presence of *P. aeruginosa*. Similar results were observed in human macrophage experiments, with normal caspase-4 activation and GSDMD cleavage after *P. aeruginosa* infection (Figure 1—figure supplement 1A, B). However, the activation of caspase-1 was suppressed and IL-1β could not be secreted normally in these cells (Figure 1—figure supplement 1A, C). With *P. aeruginosa* infection, the cell mortality rate was increased (Figure 1—figure supplement 1D) and the survival rate was decreased (Figure 1—figure supplement 1E), showing no much difference with cells treated only with intracellular *E. coli*.

**Figure 1—figure supplement 1.**
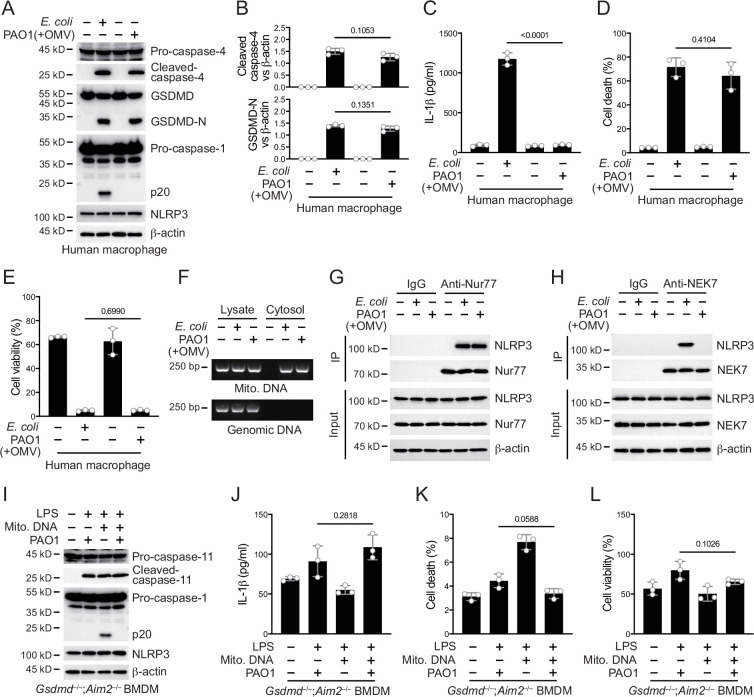
*P. aeruginosa* infection suppresses NLRP3 inflammasome in human macrophages. (**A–E**) Human macrophages were primed overnight with 1 μg/ml Pam3CSK4 and incubated with *E. coli* or *P. aeruginosa* (ΔRetS PAO1) at a multiplicity of infection (MOI) of 30 and 20 μg/ml outer membrane vesicles (OMVs) for 2 hr. Cells were then supplemented with fresh medium containing 100 μg/ml Gentamycin. Cells were lysed and immunoblotted 16 hr post infection (**A**). Band intensities of cleaved caspase-4 (top) and GSDMD (bottom) were quantified and compared to that of β-actin (**B**). Cell culture supernatants were collected for an ELISA assay to determine the secreted IL-1β protein levels 16 hr post infection (**C**). Cytotoxicity was determined by lactate dehydrogenase (LDH) release assay in cell culture supernatants 16 hr post infection (**D**). Cell viability was determined by an ATP quantification assay in cell pellets 16 hr post infection (**E**). (**F**) Wild-type BMDM cells were primed overnight with 1 μg/ml Pam3CSK4 and incubated with *E. coli* or ΔRetS PAO1 at an MOI of 30 and 20 μg/ml OMVs for 2 hr. Cells were then supplemented with fresh medium containing 100 μg/ml Gentamycin. Cells were subjected to cellular component fractionation and PCR analysis of mitochondrial or genomic DNAs 16 hr post infection. (**G, H**) Wild-type BMDM cells were primed overnight with 1 μg/ml Pam3CSK4 and incubated with *E. coli* or ΔRetS PAO1 at an MOI of 30 and 20 μg/ml OMVs for 2 hr. Cells were then supplemented with fresh medium containing 100 μg/ml Gentamycin. Cells were lysed and immunoprecipitated with a control IgG or antibody against Nur77 (**G**) or NEK7 (**H**) 16 hr post infection. Precipitates were immunoblotted as indicated. (**I–L**) *Gsdmd*^–/–^;*Aim2*^–/–^ BMDM cells were primed overnight with 1 μg/ml Pam3CSK4, followed by transfection of 2 μg/ml LPS and 2 μg/ml extracted BMDM mitochondrial DNA using DOTAP with or without the incubation of ΔRetS PAO1 at an MOI of 30 for 2 hr. Cells were then supplemented with fresh medium containing 100 μg/ml Gentamycin. Cells were lysed and immunoblotted 16 hr post infection (**I**). Cell culture supernatants were collected for an ELISA assay to determine the secreted IL-1β protein levels 16 hr post infection (**J**). Cytotoxicity was determined by LDH release assay in cell culture supernatants 16 hr post infection (**K**). Cell viability was determined by an ATP quantification assay in cell pellets 16 hr post infection (**L**). Data were shown as means ± SD. For C–E, J–L, data of three independent experiments were calculated. Experiments were repeated three times with similar results. Figure 1—figure supplement 1—source data 1.File containing labeled original western blots for Figure 1—figure supplement 1. Figure 1—figure supplement 1—source data 2.Original gel image files for western blot analysis displayed in Figure 1—figure supplement 1.

There might be two mechanisms by which NLRP3 inflammasomes were inhibited during this process. One was that the NLRP3 inflammasome lacked necessary stimuli for activation, and the other was that the NLRP3 inflammasome was inhibited by a bacterial substance. After the activation of caspase-11, GSDMD pores can be detected in mitochondrial membranes, causing the release of mtDNA and ROS which amplify NLRP3 activation (Miao et al., 2023; Xian et al., 2022). We first detected the localization of GSDMD N-terminus on the mitochondrial membrane during *P. aeruginosa* infection. The results indicate that GSDMD-N can be normally localized on the mitochondrial membrane (Figure 1F). In addition, the release of mitochondrial DNA into the cytoplasm was not affected by *P. aeruginosa* infection (Figure 1—figure supplement 1F). The binding of NUR77 and NLRP3 was not affected during the infection of *P. aeruginosa* (Figure 1—figure supplement 1G). However, the binding of NLRP3 to NEK7 was significantly inhibited (Figure 1—figure supplement 1H). These data suggest that the inhibition of NLRP3 is potentially influenced by a certain inhibitory factor from *P. aeruginosa*.

To verify this, we introduced LPS and mitochondrial DNA into macrophages to induce NLRP3 inflammasome activation, bypassing the caspase-11-GSDMD prerequisite (Zhu et al., 2023). To avoid the activation of the AIM2 inflammasome by mitochondrial DNA, we conducted the following experiments under an *Aim2* knockout background. We found that after introducing LPS and mitochondrial DNA, NLRP3 inflammasomes were activated (Figure 1G). However, NLRP3 activation was inhibited (Figure 1G) and the secretion of IL-1β was tremendously inhibited (Figure 1H) after incubation with *P. aeruginosa*. The percentage of cells undergoing pyroptosis (Figure 1I) and the cell viability (Figure 1J) did not change due to infection with *P. aeruginosa*. This was consistent with previous reports suggesting that GSDMD cleavage by caspase-11, rather than subsequent GSDMD cleavage by NLRP3, is the main factor causing cell death (Broz et al., 2020; Kayagaki et al., 2011). Next, we used *Gsdmd* and *Aim2* double knockout cells to detect the effect of *P. aeruginosa* on NLRP3 inflammasome activation. We found that after *Gsdmd* knockout, the NLRP3 inflammasome became active when LPS and mitochondrial DNA were introduced into macrophages. However, NLRP3 was still inhibited after incubation with *P. aeruginosa* (Figure 1—figure supplement 1I). The secretion of IL-1β was also inhibited due to *Gsdmd* deficiency (Figure 1—figure supplement 1J). The cell death rate was low due to the absence of GSDMD (Figure 1—figure supplement 1K), while the percentage of viable cells was relatively high (Figure 1—figure supplement 1L). These results suggest that potent inhibitory factors of *P. aeruginosa* may act upstream of GSDMD. Therefore, we conducted infection experiments using *Casp11* and *Aim2* double knockout cells. After incubation with *P. aeruginosa*, the activation of NLRP3 inflammasome was not affected (Figure 1K). The secretion of IL-1β was no longer inhibited (Figure 1L). And the cell death rate was high due to the processing of GSDMD by activated caspase-1 (Figure 1M), while the survival rate was low (Figure 1N). These data indicate that the inhibitory factors of *P. aeruginosa* requires caspase-11 to take effect.

### Caspase-11 cleaves VgrG2b of *P. aeruginosa*

**Figure 2.**
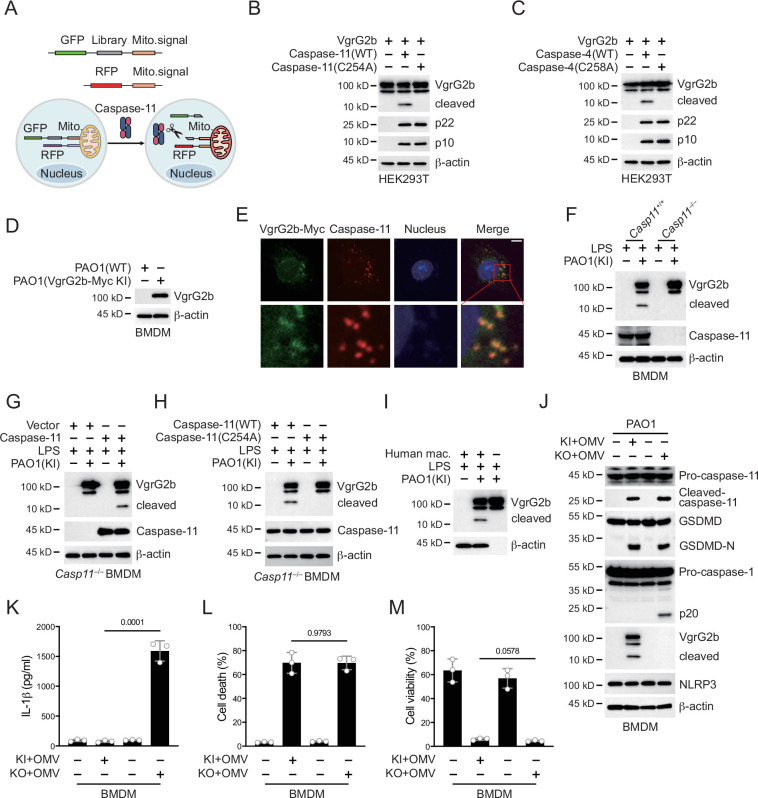
*P. aeruginosa* VgrG2b is cleaved by caspase-11. (**A**) Screening strategy for identifying caspase-11 substrates in *P. aeruginosa*. Briefly, *P. aeruginosa* cDNAs were cloned into GFP vectors containing a mitochondrial localization signal. RFP vectors with a mitochondrial localization signal were used as transfection controls. HEK293T cells were co-transfected with these vectors and caspase-11. Cells losing GFP signals on mitochondria were sequenced. Plasmids encoding VgrG2b and mouse caspase-11 p22/p10 (**B**) or human caspase-4 p22/p10 (**C**) were co-transfected into HEK293T cells for 24 hr, followed by immunoblotting with antibodies against the indicated proteins. (**D**) Wild-type BMDM cells were primed overnight with 1 μg/ml Pam3CSK4 and incubated with ΔRetS PAO1 or VgrG2b-Myc knockin ΔRetS PAO1 at a multiplicity of infection (MOI) of 30 and 20 μg/ml outer membrane vesicles (OMVs) for 2 hr. Cells were then supplemented with fresh medium containing 100 μg/ml Gentamycin. Cells were lysed and immunoblotted as indicated 16 hr post infection. (**E**) Wild-type BMDM cells were primed overnight with 1 μg/ml Pam3CSK4 and incubated with VgrG2b-Myc knockin ΔRetS PAO1 at an MOI of 30 and 20 μg/ml OMVs for 2 hr. Cells were then supplemented with fresh medium containing 100 μg/ml Gentamycin. Cells were fixed and stained with antibodies against Myc or caspase-11 16 hr post infection. Nucleus was stained with DAPI. Scale bar, 5 μm. (**F**) *Casp11*^+/+^ and *Casp11*^–/–^ BMDM cells were primed overnight with 1 μg/ml Pam3CSK4, followed by transfection of 2 μg/ml lipopolysaccharide (LPS) using DOTAP with or without the incubation of VgrG2b-Myc knockin ΔRetS PAO1 at an MOI of 30 for 2 hr. Cells were then supplemented with fresh medium containing 100 μg/ml Gentamycin. Cells were lysed and immunoblotted 16 hr post infection. (**G**) *Casp11*^–/–^ BMDM cells were infected with lentiviruses encoding a control vector of caspase-11. Cells were primed overnight with 1 μg/ml Pam3CSK4, followed by transfection of 2 μg/ml LPS using DOTAP with or without the incubation of VgrG2b-Myc knockin ΔRetS PAO1 at an MOI of 30 for 2 hr. Cells were then supplemented with fresh medium containing 100 μg/ml Gentamycin. Cells were lysed and immunoblotted as indicated 16 hr post infection. (**H**) *Casp11*^–/–^ BMDM cells were infected with lentiviruses encoding wild-type or mutant caspase-11. Cells were primed overnight with 1 μg/ml Pam3CSK4, followed by transfection of 2 μg/ml LPS using DOTAP with or without the incubation of VgrG2b-Myc knockin ΔRetS PAO1 at an MOI of 30 for 2 hr. Cells were then supplemented with fresh medium containing 100 μg/ml Gentamycin. Cells were lysed and immunoblotted as indicated 16 hr post infection. (**I**) Human macrophages were primed overnight with 1 μg/ml Pam3CSK4, followed by transfection of 2 μg/ml LPS using DOTAP with or without the incubation of VgrG2b-Myc knockin ΔRetS PAO1 at an MOI of 30 for 2 hr. Cells were then supplemented with fresh medium containing 100 μg/ml Gentamycin. Cells were lysed and immunoblotted as indicated 16 hr post infection. (**J–M**) Wild-type BMDM cells were primed overnight with 1 μg/ml Pam3CSK4, followed by incubation of VgrG2b-Myc knockin (KI) or knockout (KO) ΔRetS PAO1 at an MOI of 30 and 20 μg/ml OMVs for 2 hr. Cells were then supplemented with fresh medium containing 100 μg/ml Gentamycin. Cells were lysed and immunoblotted as indicated 16 hr post infection (**J**). Cell culture supernatants were collected for an ELISA assay to determine the secreted IL-1β protein levels 16 hr post infection (**K**). Cytotoxicity was determined by lactate dehydrogenase (LDH) release assay in cell culture supernatants 16 hr post infection (**L**). Cell viability was determined by an ATP quantification assay in cell pellets 16 hr post infection (**M**). Data were shown as means ± SD. For K–M, data of three independent experiments were calculated. Experiments were repeated three times with similar results. Figure 2—source data 1.File containing labeled original western blots for Figure 2. Figure 2—source data 2.Original gel image files for western blot analysis displayed in Figure 2. Figure 2—source data 3.Original source data for graphs displayed in Figure 2, Figure 2—figure supplement 1.

In order to search for potential substrates of caspase-11 in *P. aeruginosa*, we designed a systematic screening system (Figure 2A). Simply, we constructed two fluorescent plasmids, one containing green fluorescent protein (GFP), polyclonal sites, and mitochondrial outer membrane localization signals, and the other containing red fluorescent protein (RFP) and mitochondrial outer membrane localization signals. We extracted the mRNA of *P. aeruginosa* and prepared it into a library for insertion into GFP containing plasmids. We co-transfected it with the RFP plasmid into HEK293T cells and sorted the monoclonal cells containing both RFP and GFP signals into a 96-well plate using flow cytometry. Subsequently, the plasmid encoding caspase-11 was transfected into cells, and cells with disappearance of green signal on mitochondria were selected. The target proteins in these cells were cleaved by caspase-11, causing the detachment of GFP from mitochondria. After sequencing, we found that VgrG1a and VgrG2b repeatedly appeared in cells that met the screening criteria.

**Figure 2—figure supplement 1.**
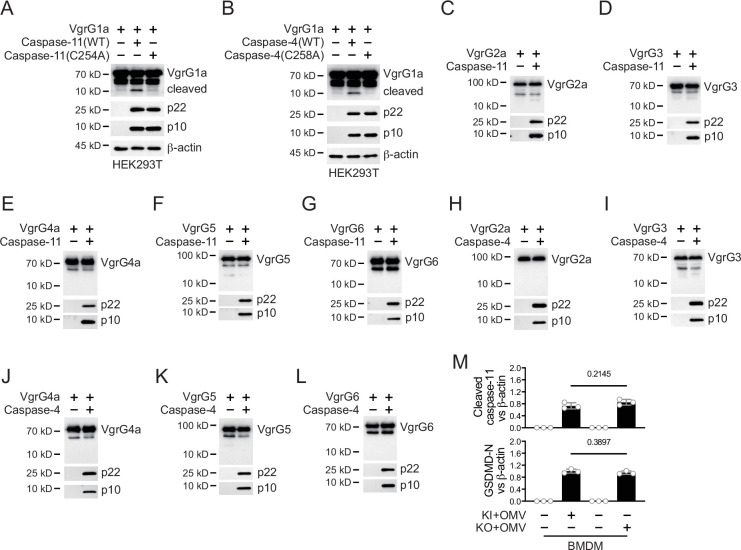
Caspase-11 cleaves limited VgrG family members. Plasmids encoding VgrG1a and mouse caspase-11 p22/p10 (**A**) or human caspase-4 p22/p10 (**B**) were co-transfected into HEK293T cells for 24 hr, followed by immunoblotting with antibodies against the indicated proteins. (**C–L**) Plasmids encoding the indicated VgrG family members and mouse caspase-11 p22/p10 or human caspase-4 p22/p10 were co-transfected into HEK293T cells for 24 hr, followed by immunoblotting with antibodies against the indicated proteins. (**M**) Wild-type BMDM cells were primed overnight with 1 μg/ml Pam3CSK4, followed by incubation of VgrG2b-Myc knockin (KI) or knockout (KO) ΔRetS PAO1 at a multiplicity of infection (MOI) of 30 and 20 μg/ml outer membrane vesicles (OMVs) for 2 hr. Cells were then supplemented with fresh medium containing 100 μg/ml Gentamycin. Cells were lysed and immunoblotted 16 hr post infection. Band intensities of cleaved caspase-11 (top) and GSDMD (bottom) were quantified and compared to that of β-actin. Data were shown as means ± SD. Experiments were repeated three times with similar results. Figure 2—figure supplement 1—source data 1.File containing labeled original western blots for Figure 2—figure supplement 1. Figure 2—figure supplement 1—source data 2.Original gel image files for western blot analysis displayed in Figure 2—figure supplement 1.

We cloned the genes of VgrG1a and VgrG2b and cleaved them using mouse caspase-11. We found that they can both be cleaved (Figure 2—figure supplement 1A, Figure 2B). We also found that they can be cleaved by human caspase-4 (Figure 2—figure supplement 1B, Figure 2C), but not by caspase-11/4 mutants lacking enzymatic activities. These results indicate that our screening system is reliable. The number of target genes screened was very limited, which was consistent with the particularly narrow substrate spectrum of caspae-11 (Shi et al., 2023). We also tested other members of the VgrG family and found that neither mouse caspase-11 nor human caspase-4 could cleave these members (Figure 2—figure supplement 1C–L). VgrG1a and VgrG2b belong to the secretion systems of H1-T6SS and H2-T6SS, and H1-T6SS does not have an effect on eukaryotic cells (Hood et al., 2010; Sana et al., 2015). Therefore, our subsequent research focused on VgrG2b.

Our next concern was whether VgrG2b entered macrophages. We found that after co-culturing of *P. aeruginosa* with BMDM cells, the VgrG2b protein appeared in macrophages (Figure 2D). Immunofluorescence experiments also showed the presence of bacterial proteins in macrophages after co-incubation with *P. aeruginosa* (Figure 2E). We then incubated *P. aeruginosa* with wild-type BMDM cells and found that VgrG2b was cleaved in macrophages, while the cleavage band of VgrG2b disappeared in *Casp11*^–/–^ BMDM cells (Figure 2F). We restored caspase-11 expression in *Casp11*^–/–^ BMDM cells and found that the presence of caspase-11 induced VgrG2b cleavage (Figure 2G). We also rescued *Casp11*^–/–^ BMDM cells with wild-type and enzymatic mutant caspase-11 and found that mutant caspase-11 could not induce VgrG2b cleavage (Figure 2H). In addition, we incubated human macrophages with *P. aeruginosa*. As expected, VgrG2b was cleaved in human cells (Figure 2I). Finally, to verify that *P. aeruginosa* affected inflammasome activation through H2-T6SS, we generated a *P. aeruginosa* strain with VgrG2b deficiency. We found that *P. aeruginosa* lacking VgrG2b no longer inhibited the NLRP3 inflammasome (Figure 2J, Figure 2—figure supplement 1M) and IL-1β could be secreted normally in the mutant bacteria-treated cells (Figure 2K). However, the percentage of cell death was not affected (Figure 2L) and the level of cell viability remained unchanged among mutants and wild-type controls (Figure 2M). These data indicate that H2-T6SS plays an important role in inhibiting host immune response through VgrG2b during *P. aeruginosa* infection, primarily targeting NLRP3 inflammasome activation rather than caspase-11 activation and GSDMD cleavage.

### VgrG2b cleavage at its C-terminus is crucial for NLRP3 inhibition

**Figure 3.**
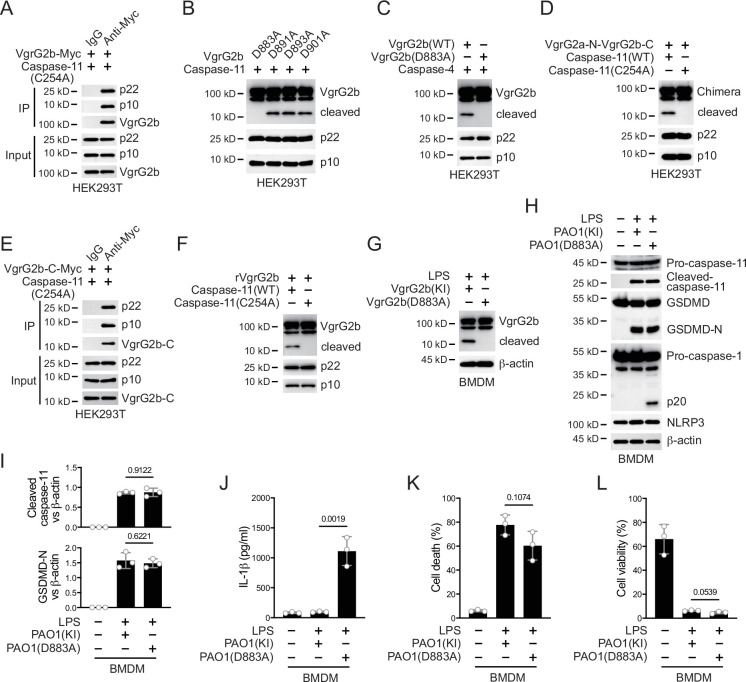
Caspase-11 cleaves VgrG2b at D883. (**A**) Plasmids encoding VgrG2b and mutant caspase-11 p22/p10 were co-transfected into HEK293T cells for 24 hr, followed by immunoprecipitation with a control IgG or antibody against Myc. Precipitates were immunoblotted as indicated. (**B**) Plasmids encoding caspase-11 p22/p10 and VgrG2b mutants were co-transfected into HEK293T cells for 24 hr, followed by immunoblotting with antibodies against the indicated proteins. (**C**) Plasmids encoding caspase-4 p22/p10 and VgrG2b variants were co-transfected into HEK293T cells for 24 hr, followed by immunoblotting with antibodies against the indicated proteins. (**D**) Plasmids encoding VgrG2a-N-VgrG2b-C chimera and caspase-11 p22/p10 were co-transfected into HEK293T cells for 24 hr, followed by immunoblotting with antibodies against the indicated proteins. (**E**) Plasmids encoding VgrG2b C-terminus and mutant caspase-11 p22/p10 were co-transfected into HEK293T cells for 24 hr, followed by immunoprecipitation with a control IgG or antibody against Myc. Precipitates were immunoblotted as indicated. (**F**) Recombinant VgrG2b were incubated with caspase-11 p22/p10 for 4 hr, followed by immunoblotting with antibodies against the indicated proteins. (**G**) Wild-type BMDM cells were primed overnight with 1 μg/ml Pam3CSK4, followed by transfection of 2 μg/ml lipopolysaccharide (LPS) using DOTAP with or without the incubation of VgrG2b wild-type (KI) or mutant (D883A) knockin ΔRetS PAO1 at a multiplicity of infection (MOI) of 30 for 2 hr. Cells were then supplemented with fresh medium containing 100 μg/ml Gentamycin. Cells were lysed and immunoblotted 16 hr post infection. (**H–L**) Wild-type BMDM cells were primed overnight with 1 μg/ml Pam3CSK4, followed by transfection of 2 μg/ml LPS using DOTAP with or without the incubation of VgrG2b wild-type (KI) or mutant (D883A) knockin ΔRetS PAO1 at an MOI of 30 for 2 hr. Cells were then supplemented with fresh medium containing 100 μg/ml Gentamycin. Cells were lysed and immunoblotted as indicated 16 hr post infection (**H**). Band intensities of cleaved caspase-11 (top) and GSDMD (bottom) were quantified and compared to that of β-actin (**I**). Cell culture supernatants were collected for an ELISA assay to determine the secreted IL-1β protein levels 16 hr post infection (**J**). Cytotoxicity was determined by lactate dehydrogenase (LDH) release assay in cell culture supernatants 16 hr post infection (**K**). Cell viability was determined by an ATP quantification assay in cell pellets 16 hr post infection (**L**). Data were shown as means ± SD. For J–L, data of three independent experiments were calculated. Experiments were repeated three times with similar results. Figure 3—source data 1.File containing labeled original western blots for Figure 3. Figure 3—source data 2.Original gel image files for western blot analysis displayed in Figure 3. Figure 3—source data 3.Original source data for graphs displayed in Figure 3, Figure 3—figure supplement 1.

Caspase-11 recognizes its substrate through a tertiary structure formed by its p22/p10 fragments (Wang et al., 2020). To further verify that VgrG2b was a substrate of caspase-11, we co-expressed enzymatically inactive mutant caspase-11 p22/p10 and VgrG2b in HEK293T cells. Co-immunoprecipitation results showed that VgrG2b bound to the p22/p10 complex (Figure 3A). VgrG2b also bound to the p22/p10 complex of mutant human caspase-4 (Figure 3—figure supplement 1A). These results suggest that VgrG2b can indeed be recognized by caspase-11/4. Based on the size of the cleaved VgrG2b fragment, we speculated that the cleavage site should be at its C-terminus. We constructed mutant plasmids carrying aspartic acid substitutions for VgrG2b and found that only the D883A mutant was no longer cleaved by caspase-11 (Figure 3B). We also verified that this VgrG2b mutant could not be cleaved by human caspase-4 (Figure 3C). Meanwhile, we constructed a chimeric protein containing VgrG2a N-terminus, a linker sequence around D883 and VgrG2b C-terminus (Figure 3—figure supplement 1B). This chimeric protein could be cleaved by caspase-11 (Figure 3D). Another chimeric protein containing VgrG2b N-terminus, a linker sequence around D883 and GFP cannot be cleaved by caspase-11 (Figure 3—figure supplement 1B, C). Based on the substrate cleavage characteristic of caspase-11 that it needs to bind the target first to mediate cleavage (Wang et al., 2020), we speculated that the VgrG2b C-terminus was the site where VgrG2b bound to caspase-11. Therefore, we tested the binding of VgrG2b C-terminus with caspase-11. Indeed, the C-terminus of VgrG2b bound to the p22/p10 complex (Figure 3E). Similarly, VgrG2b C-terminus also bound to p22/p10 of human caspase-4 (Figure 3—figure supplement 1D). VgrG2b was recombinantly expressed and incubated with the p22/p10 complex. We found that caspase-11 induced the cleavage of the recombinant VgrG2b (Figure 3F, Figure 3—figure supplement 1E).

**Figure 3—figure supplement 1.**
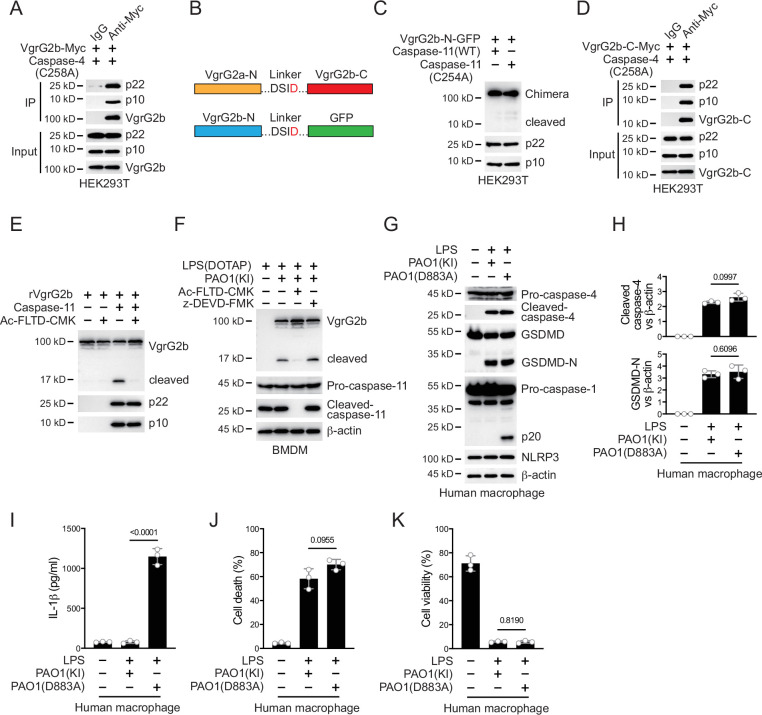
Caspase-11 cleaves VgrG2b during *P. aeruginosa* infection. (**A**) Plasmids encoding VgrG2b and mutant caspase-4 p22/p10 were co-transfected into HEK293T cells for 24 hr, followed by immunoprecipitation with a control IgG or antibody against Myc. Precipitates were immunoblotted as indicated. (**B**) Scheme for VgrG2b chimeras. (**C**) Plasmids encoding VgrG2b-N-GFP chimera and caspase-11 p22/p10 were co-transfected into HEK293T cells for 24 hr, followed by immunoblotting with antibodies against the indicated proteins. (**D**) Plasmids encoding VgrG2b-C and mutant caspase-4 p22/p10 were co-transfected into HEK293T cells for 24 hr, followed by immunoprecipitation with a control IgG or antibody against Myc. Precipitates were immunoblotted as indicated. (**E**) Recombinant VgrG2b was incubated with caspase-11 p22/p10 subunits with or without the presence of 10 μM Ac-FLTD-CMK, followed by immunoblotting with antibodies against the indicated proteins. (**F**) BMDM cells were primed overnight with 1 μg/ml Pam3CSK4, followed by transfection of 2 μg/ml lipopolysaccharide (LPS) using DOTAP with or without the incubation of VgrG2b-Myc knockin (KI) ΔRetS PAO1 at a multiplicity of infection (MOI) of 30 for 2 hr. Cells were then supplemented with fresh medium containing 100 μg/ml Gentamycin and 10 μM Ac-FLTD-CMK or 10 μM z-DEVD-FMK. Cells were lysed and immunoblotted 16 hr post infection. (**G–K**) Human macrophages were primed overnight with 1 μg/ml Pam3CSK4, followed by transfection of 2 μg/ml LPS using DOTAP with or without the incubation of VgrG2b (KI) or mutant (D883A) knockin ΔRetS PAO1 at an MOI of 30 for 2 hr. Cells were then supplemented with fresh medium containing 100 μg/ml Gentamycin. Cells were lysed and immunoblotted as indicated 16 hr post infection (**G**). Band intensities of cleaved caspase-4 (top) and GSDMD (bottom) were quantified and compared to that of β-actin (**H**). Cell culture supernatants were collected for an ELISA assay to determine the secreted IL-1β protein levels 16 hr post infection (**I**). Cytotoxicity was determined by lactate dehydrogenase (LDH) release assay in cell culture supernatants 16 hr post infection (**J**). Cell viability was determined by an ATP quantification assay in cell pellets 16 hr post infection (**K**). Data were shown as means ± SD. For I–K, data of three independent experiments were calculated. Experiments were repeated three times with similar results. Figure 3—figure supplement 1—source data 1.File containing labeled original western blots for Figure 3—figure supplement 1. Figure 3—figure supplement 1—source data 2.Original gel image files for western blot analysis displayed in Figure 3—figure supplement 1.

We generated a *P. aeruginosa* strain with a D883A mutation in the VgrG2b gene. After incubating this bacterium with mouse BMDM, mutant VgrG2b did not undergo cleavage (Figure 3G). Consistently, the NLRP3 inflammasome was no longer inhibited (Figure 3H, I) and the secretion of IL-1β was restored post incubation with this *P. aeruginosa* mutant (Figure 3J), indicating that VgrG2b cleavage by caspase-11 is crucial for NLRP3 inhibition. The cell death and survival rates were not affected (Figure 3K, L). Moreover, inhibition of caspase-11 but not caspase-3 or -7 blocked VgrG2b cleavage (Figure 3—figure supplement 1F). Similarly, after incubation of the *P. aeruginosa* mutant with human macrophages, the NLRP3 inflammasome was not suppressed (Figure 3—figure supplement 1G, H) and IL-1β was secreted normally (Figure 3—figure supplement 1I). The cell death and survival rates were not affected (Figure 3—figure supplement 1J, K). These results indicate that VgrG2b cleavage at its C-terminus is crucial for NLRP3 inhibition.

### VgrG2b C-terminus interacts with NLRP3

**Figure 4.**
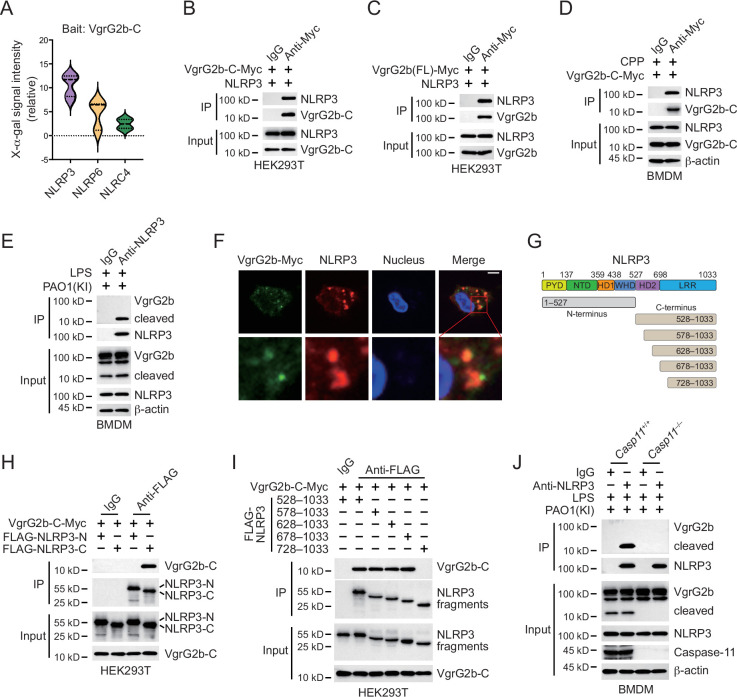
VgrG2b C-terminus interacts with NLRP3. (**A**) Yeast two-hybrid screening was performed using VgrG2b C-terminus as bait and a mouse bone marrow cDNA library was screened. The interaction strength between bait and preys were visualized by X-α-gal assays. Plasmids encoding VgrG2b C-terminus (**B**) or full-length (**C**) and NLRP3 were co-transfected into HEK293T cells for 24 hr, followed by immunoprecipitation with a control IgG or antibody against Myc. Precipitates were immunoblotted as indicated. (**D**) VgrG2b C-terminus proteins were introduced into BMDM cells with the help of cell-penetrating peptides (CPP) for 6 hr. Cells were lysed and immunoprecipitated with a control IgG or antibody against Myc. Precipitates were immunoblotted as indicated. (**E**) BMDM cells were primed overnight with 1 μg/ml Pam3CSK4, followed by transfection of 2 μg/ml lipopolysaccharide (LPS) using DOTAP with the incubation of VgrG2b knockin (KI) ΔRetS PAO1 at a multiplicity of infection (MOI) of 30 for 2 hr. Cells were then supplemented with fresh medium containing 100 μg/ml Gentamycin. Cells were lysed and immunoprecipitated with a control IgG or antibody against NLRP3 16 hr post infection. Precipitates were immunoblotted as indicated. (**F**) BMDM cells were primed overnight with 1 μg/ml Pam3CSK4, followed by transfection of 2 μg/ml LPS using DOTAP with the incubation of VgrG2b knockin (KI) ΔRetS PAO1 at an MOI of 30 for 2 hr. Cells were then supplemented with fresh medium containing 100 μg/ml Gentamycin. Cells were fixed and stained with antibodies against Myc and NLRP3 16 hr post infection. Nucleus was stained with DAPI. Scale bar, 5 μm. (**G**) Scheme for NLRP3 truncations. (**H, I**) Plasmids encoding VgrG2b C-terminus and NLRP3 truncations were co-transfected into HEK293T cells for 24 hr, followed by immunoprecipitation with a control IgG or antibody against FLAG. Precipitates were immunoblotted as indicated. (**J**) *Casp11*^+/+^ and *Casp11*^–/–^ BMDM cells were primed overnight with 1 μg/ml Pam3CSK4, followed by transfection of 2 μg/ml LPS using DOTAP with the incubation of VgrG2b-Myc knockin (KI) ΔRetS PAO1 at an MOI of 30 for 2 hr. Cells were then supplemented with fresh medium containing 100 μg/ml Gentamycin. Cells were lysed and immunoprecipitated with a control IgG or antibody against NLRP3 16 hr post infection. Precipitates were immunoblotted as indicated. Data were shown as means ± SD. Experiments were repeated three times with similar results. Figure 4—source data 1.File containing labeled original western blots for Figure 4. Figure 4—source data 2.Original gel image files for western blot analysis displayed in Figure 4. Figure 4—source data 3.Original source data for graphs displayed in Figure 4.

In order to investigate how VgrG2b C-terminus inhibited the NLRP3 inflammasome, we performed a yeast two-hybrid screening using the C-terminus of VgrG2b as a bait and screened a mouse bone marrow cDNA library. In the positive clones obtained from the screening, the NLRP3 fragments repeatedly appeared (Figure 4A). We validated their interaction in HEK293T cells and found that VgrG2b C-terminus indeed bound to NLRP3 (Figure 4B). Full-length VgrG2b also bound to NLRP3 (Figure 4C), but the N-terminus of VgrG2b did not bind to NLRP3 (Figure 4—figure supplement 1A). VgrG2b C-terminus was introduced into BMDM cells through cell-penetrating peptides, and it was found that the C-terminus interacted with endogenous NLRP3 (Figure 4D). Similarly, VgrG2b C-terminus also interacted with the human NLRP3 (Figure 4—figure supplement 1B). In addition, we incubated *P. aeruginosa* with mouse BMDM cells and found that NLRP3 interacted with the C-terminus of VgrG2b rather than the full-length one (Figure 4E). The same result was observed after incubation of *P. aeruginosa* with human macrophages, where NLRP3 bound to the C-terminus of VgrG2b (Figure 4—figure supplement 1C). The colocalization between VgrG2b C-terminus and NLRP3 was observed inside BMDM cells (Figure 4F). The VgrG2b C-terminus did not bind to mouse GSDMD (Figure 4—figure supplement 1D), ASC (Figure 4—figure supplement 1E), nor caspase-1 (Figure 4—figure supplement 1F, G), indicating that the binding targets of VgrG2b in host cells is very specific. We further searched for the region where VgrG2b C-terminus interacted with NLRP3 (Figure 4G), and found that their interaction area was at the NACHT–LRR junction of NLRP3 (Figure 4H, I).

**Figure 4—figure supplement 1.**
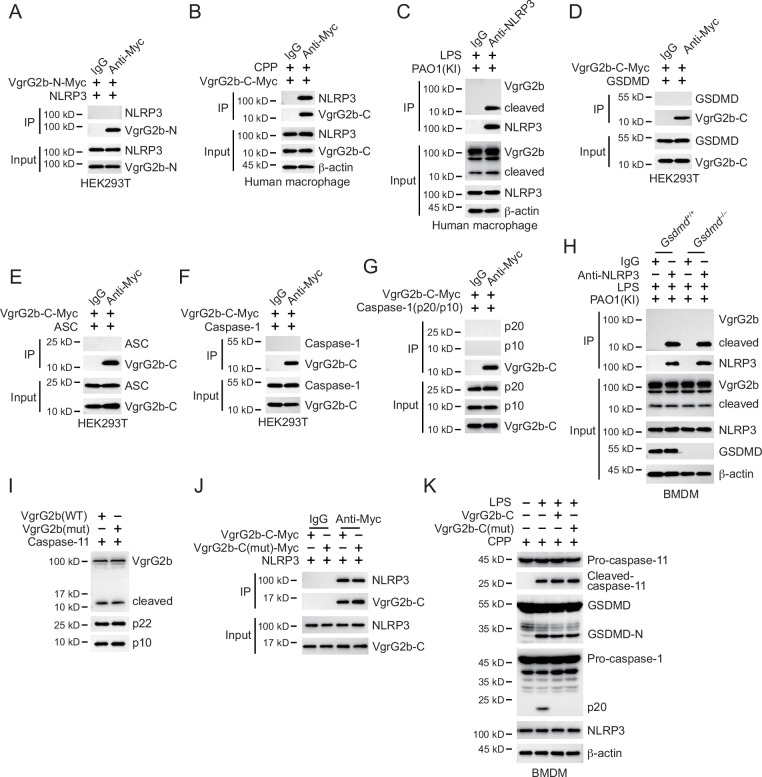
Cleaved VgrG2b fragment binds to NLRP3. (**A**) Plasmids encoding VgrG2b N-terminus and NLRP3 were co-transfected into HEK293T cells for 24 hr, followed by immunoprecipitation with a control IgG or antibody against Myc. Precipitates were immunoblotted as indicated. (**B**) VgrG2b C-terminus proteins were introduced into human macrophage cells with the help of cell-penetrating peptides (CPP) for 6 hr. Cells were lysed and immunoprecipitated with a control IgG or antibody against Myc. Precipitates were immunoblotted as indicated. (**C**) Human macrophage cells were primed overnight with 1 μg/ml Pam3CSK4, followed by transfection of 2 μg/ml lipopolysaccharide (LPS) using DOTAP with the incubation of VgrG2b knockin (KI) ΔRetS PAO1 at a multiplicity of infection (MOI) of 30 for 2 hr. Cells were then supplemented with fresh medium containing 100 μg/ml Gentamycin. Cells were lysed and immunoprecipitated with a control IgG or antibody against NLRP3 16 hr post infection. Precipitates were immunoblotted as indicated. Plasmids encoding VgrG2b C-terminus and GSDMD (**D**), ASC (**E**), caspase-1 (**F**), and caspase-1 p20/p10 (**G**) were co-transfected into HEK293T cells for 24 hr, followed by immunoprecipitation with a control IgG or antibody against Myc. Precipitates were immunoblotted as indicated. (**H**) *Gsdmd*^+/+^ and *Gsdmd*^–/–^ BMDM cells were primed overnight with 1 μg/ml Pam3CSK4, followed by transfection of 2 μg/ml LPS using DOTAP with the incubation of VgrG2b-Myc knockin (KI) ΔRetS PAO1 at an MOI of 30 for 2 hr. Cells were then supplemented with fresh medium containing 100 μg/ml Gentamycin. Cells were lysed and immunoprecipitated with a control IgG or antibody against NLRP3 16 hr post infection. (**I**) Plasmids encoding wild-type VgrG2b, mutant VgrG2b (H935A;H936A;H939A;E983A), and caspase-11 p22/p10 were co-transfected into HEK293T cells for 24 hr, followed by immunoblotting with antibodies against the indicated proteins. (**J**) Plasmids encoding wild-type VgrG2b C-terminus, mutant VgrG2b C-terminus (H935A;H936A;H939A;E983A) and NLRP3 were co-transfected into HEK293T cells for 24 hr, followed by immunoprecipitation with a control IgG or antibody against Myc. Precipitates were immunoblotted as indicated. (**K**) BMDM cells were primed overnight with 1 μg/ml Pam3CSK4. Recombinant wild-type VgrG2b C-terminus and mutant VgrG2b C-terminus (H935A;H936A;H939A;E983A) were introduced into BMDM cells with the help of cell-penetrating peptides (CPP) for 6 hr, followed by transfection of 2 μg/ml LPS using DOTAP. Cells were immunoblotted with antibodies against the indicated proteins. Precipitates were immunoblotted as indicated. Data were shown as means ± SD. Experiments were repeated three times with similar results. Figure 4—figure supplement 1—source data 1.File containing labeled original western blots for Figure 4—figure supplement 1. Figure 4—figure supplement 1—source data 2.Original gel image files for western blot analysis displayed in Figure 4—figure supplement 1.

In *Casp11* knockout BMDM cells, VgrG2b could not be cleaved and did not bind to NLRP3 (Figure 4J). However, in *Gsdmd* knockout cells, VgrG2b still underwent cleavage and bound to NLRP3 (Figure 4—figure supplement 1H). In the absence of GSDMD, the NLRP3 inflammasome was not activated (Broz et al., 2020; Kayagaki et al., 2015). This result indicates that the binding of VgrG2b C-terminus to NLRP3 is independent of NLRP3 activation, but requires a cleaved segment of VgrG2b processed by caspase-11. VgrG2b also possesses a metalloprotease activity (Wood et al., 2019). To test whether its protease activity is responsible for VgrG2b cleavage observed in our study, we mutated VgrG2b to disable its protease activity and found that this mutant form did not prohibit caspase-11 cleavage on VgrG2b (Figure 4—figure supplement 1I). Moreover, VgrG2b mutant C-terminus bound NLRP3 and inhibited NLRP3 activation just like its wild-type counterpart (Figure 4—figure supplement 1J and K).

In all, these results suggest that the cleaved VgrG2b C-terminus binds NLRP3.

### VgrG2b C-terminus suppresses the NLRP3 inflammasome

**Figure 5.**
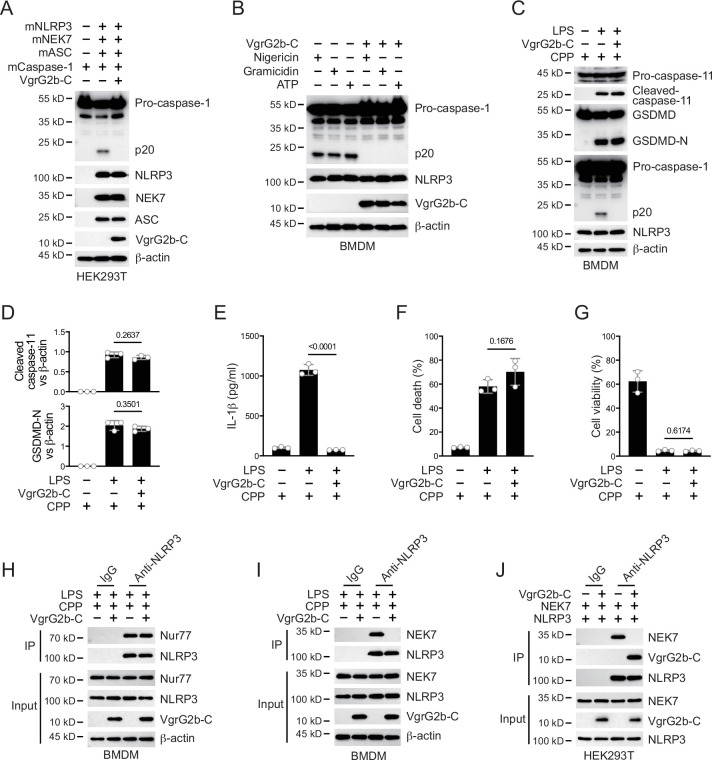
VgrG2b C-terminus suppresses the NLRP3 inflammasome. (**A**) Plasmids encoding VgrG2b C-terminus and the indicated murine proteins were co-transfected into HEK293T cells for 24 hr, followed by immunoblotting with antibodies against the indicated proteins. (**B**) Wild-type BMDM cells were infected with lentiviruses encoding a control vector or VgrG2b C-terminus and primed with 1 μg/ml lipopolysaccharide (LPS) for 3 hr, followed by stimulation of 10 μM nigericin for 30 min, 0.5 μM gramicidin for 1 hr and 2 mM ATP for 30 min. Cells were lysed and immunoblotted as indicated. (**C–G**) Wild-type BMDM cells were primed overnight with 1 μg/ml Pam3CSK4, followed by transfection of 2 μg/ml LPS using DOTAP with or without the transfection of VgrG2b C-terminus proteins using cell-penetrating peptides (CPP) for 2 hr. Cells were then supplemented with fresh medium. Cells were lysed and immunoblotted 16 hr post transfection (**C**). Band intensities of cleaved caspase-11 (top) and GSDMD (bottom) were quantified and compared to that of β-actin (**D**). Cell culture supernatants were collected for an ELISA assay to determine the secreted IL-1β protein levels 16 hr post infection (**E**). Cytotoxicity was determined by lactate dehydrogenase (LDH) release assay in cell culture supernatants 16 hr post infection (**F**). Cell viability was determined by an ATP quantification assay in cell pellets 16 hr post infection (**G**). (**H, I**) Wild-type BMDM cells were primed overnight with 1 μg/ml Pam3CSK4, followed by transfection of 2 μg/ml LPS using DOTAP with or without the transfection of VgrG2b C-terminus proteins using cell-penetrating peptides (CPP) for 2 hr. Cells were then supplemented with fresh medium. Cells were lysed and immunoprecipitated with a control IgG or antibody against NLRP3 16 hr post transfection. Precipitates were immunoblotted with antibody against Nur77 (**H**) or NEK7 (**I**). (**J**) Plasmids encoding VgrG2b C-terminus, NEK7 and NLRP3 were co-transfected into HEK293T cells for 24 hr, followed by immunoprecipitation with a control IgG or antibody against NLRP3. Precipitates were immunoblotted as indicated. Data were shown as means ± SD. For E–G, data of three independent experiments were calculated. Experiments were repeated three times with similar results. Figure 5—source data 1.File containing labeled original western blots for Figure 5. Figure 5—source data 2.Original gel image files for western blot analysis displayed in Figure 5. Figure 5—source data 3.Original source data for graphs displayed in Figure 5, Figure 5—figure supplement 1.

Whether VgrG2b C-terminus directly inhibited the activation of NLRP3 inflammasomes was the focus of our next exploration. We validated this hypothesis in the HEK293T recombination system. The VgrG2b C-terminus significantly inhibited the activation of NLRP3 inflammasome originated from mouse (Figure 5A) or human (Figure 5—figure supplement 1A). In addition, overexpression of VgrG2b C-terminus suppressed the activation of classical NLRP3 inflammasomes in both mouse (Figure 5B) and human macrophages (Figure 5—figure supplement 1B). These data indicate that VgrG2b C-terminus exhibits a broad-spectrum inhibition of the NLRP3 inflammasome. Introducing VgrG2b C-terminus into BMDM cells through cell-penetrating peptides can significantly inhibit the activation of NLRP3 post intracellular LPS stimulation, without affecting the activation of caspase-11 and GSDMD (Figure 5C, D). The secretion of IL-1β was significantly decreased (Figure 5E). The cell death (Figure 5F) and survival rates (Figure 5G) were not affected. The activation of NLRP3 could also be inhibited by introducing VgrG2b C-terminus through cell-penetrating peptides when classical NLRP3 inflammasomes stimuli were present (Figure 5—figure supplement 1C), accompanied by a reduction of IL-1β secretion (Figure 5—figure supplement 1D), a decrease in cell death (Figure 5—figure supplement 1E), and an increase in cell viability (Figure 5—figure supplement 1F).

**Figure 5—figure supplement 1.**
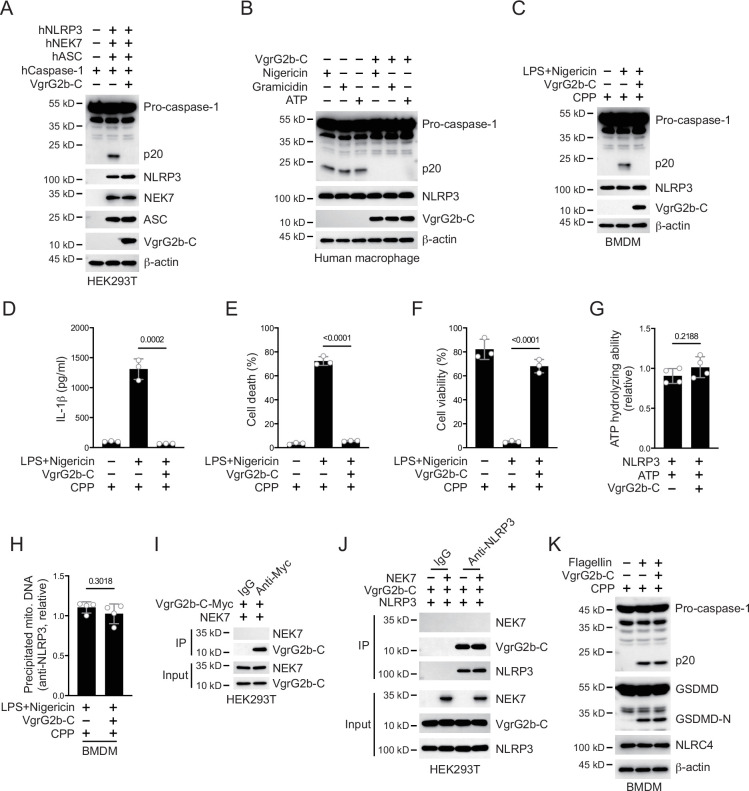
VgrG2b C-terminus inhibits NLRP3 inflammasome activation in human cells. (**A**) Plasmids encoding VgrG2b C-terminus and the indicated human proteins were co-transfected into HEK293T cells for 24 hr, followed by immunoblotting with antibodies against the indicated proteins. (**B**) Human macrophage cells were infected with lentiviruses encoding a control vector or VgrG2b C-terminus and primed with 1 μg/ml lipopolysaccharide (LPS) for 3 hr, followed by stimulation of 10 μM nigericin for 30 min, 0.5 μM gramicidin for 1 hr and 2 mM ATP for 30 min. Cells were lysed and immunoblotted as indicated. (**C–F**) Wild-type BMDM cells were primed with 1 μg/ml LPS for 3 hr, followed by stimulation of 10 μM nigericin with or without the transfection of VgrG2b C-terminus proteins using cell-penetrating peptides (CPP) for 30 min. Cells were lysed and immunoblotted (**C**). Cell culture supernatants were collected for an ELISA assay to determine the secreted IL-1β protein levels (**D**). Cytotoxicity was determined by lactate dehydrogenase (LDH) release assay in cell culture supernatants (**E**). Cell viability was determined by an ATP quantification assay in cell pellets (**F**). (**G**) FLAG-NLRP3 was purified from HEK293T cells using anti-FLAG-conjugated beads. NLRP3-containing beads were incubated with 100 μCi[γ-^32^P]ATP in the presence of 100 ng/ml VgrG2b C-terminus. Beads were washed with PBS and the associated radioactive ATP was determined by liquid scintillation. (**H**) Wild-type BMDM cells were primed with 1 μg/ml LPS for 3 hr, followed by stimulation of 10 μM nigericin with or without the transfection of VgrG2b C-terminus proteins using cell-penetrating peptides (CPP) for 30 min. Cells were lysed and immunoprecipitated with antibody against NLRP3. The amount of mitochondrial DNA in the precipitates was examined through quantitative PCR. (**I**) Plasmids encoding VgrG2b C-terminus and NEK7 were co-transfected into HEK293T cells for 24 hr, followed by immunoprecipitation with a control IgG or antibody against Myc. Precipitates were immunoblotted as indicated. (**J**) Plasmids encoding VgrG2b C-terminus, NEK7 and NLRP3 were co-transfected into HEK293T cells for 24 hr, followed by immunoprecipitation with a control IgG or antibody against NLRP3. Precipitates were immunoblotted as indicated. (**K**) Recombinant VgrG2b C-terminus proteins were introduced into BMDM cells with the help of cell-penetrating peptides (CPP) for 6 hr. Cells were then transfected with 4 μg/ml flagellin with the help of lipofectin for 4 hr. Cells were lysed and immunoprecipitated with antibodies against the indicated proteins. Data were shown as means ± SD. For D–F, data of three independent experiments were calculated. Experiments were repeated three times with similar results. Figure 5—figure supplement 1—source data 1.File containing labeled original western blots for Figure 5—figure supplement 1. Figure 5—figure supplement 1—source data 2.Original gel image files for western blot analysis displayed in Figure 5—figure supplement 1.

We next searched for the specific mechanism by which VgrG2b C-terminus inhibited the NLRP3 inflammasome. We first tested the ability of NLRP3 to catalyze ATP hydrolysis after the addition of VgrG2b C-terminus and found that the hydrolysis potential of NLRP3 was not affected (Figure 5—figure supplement 1G). Meanwhile, VgrG2b C-terminus did not influence the binding of NLRP3 to mitochondrial DNA (Figure 5—figure supplement 1H), as well as to Nur77 (Figure 5H). The binding of NLRP3 to NEK7 was immensely inhibited by the C-terminus of VgrG2b (Figure 5I). VgrG2b C-terminus did not bind to NEK7 (Figure 5—figure supplement 1I). Domain mapping showed that the VgrG2b C-terminus bound to the same NLRP3 segment as NEK7 (Figure 4G–I). We therefore detected the competitive binding between VgrG2b C-terminus and NEK7 to NLRP3. VgrG2b C-terminus competed against NEK7 (Figure 5J), while NEK7 could not compete with VgrG2b C-terminus (Figure 5—figure supplement 1J), indicating that VgrG2b C-terminus has a stronger binding ability with NLRP3 compared to NEK7. Binding assays showed that VgrG2b-C had a tight binding ability for NLRP3 than NEK7 (326 vs. 681 nM). Indeed, VgrG2b C-terminus did not inhibit the NLRC4 inflammasome (Figure 5—figure supplement 1K).

### VgrG2b cleavage is essential for *P. aeruginosa* infection

**Figure 6.**
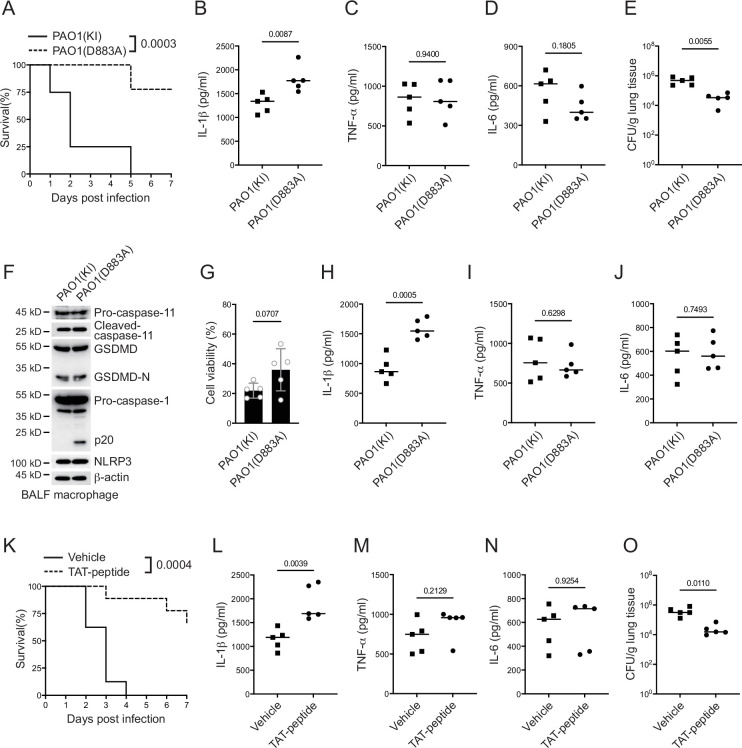
*P. aeruginosa* suppresses NLRP3 inflammasome in vivo. (**A–E**) Wild-type mice were intraperitoneally challenged with poly(I:C) at a dose of 2 mg/kg body weight for 6 hr and then intranasally infected with 1 × 10^9^ cfu of VgrG2b (KI) or mutant (D883A) knockin ΔRetS PAO1, followed by survival calculation at the indicated days (**A**). Serum protein levels of IL-1β (**B**), TNF-α (**C**), and IL-6 (**D**) were examined through ELISA assays 24 hr post infection. Bacterial load was examined in mouse lung tissues 4 days post infection (**E**). (**F–J**) Wild-type mice were intraperitoneally challenged with poly(I:C) at a dose of 2 mg/kg body weight for 6 hr and then intranasally infected with 1 × 10^9^ cfu of VgrG2b (KI) or mutant (D883A) knockin ΔRetS PAO1. Bronchoalveolar lavage fluids (BALF) were collected 16 hr post infection. Cells were immunoblotted as indicated (**F**). Cell viability was also determined by an ATP quantification assay in cell pellets (**G**). Protein levels of IL-1β (**H**), TNF-α (**I**), and IL-6 (**J**) in BALF supernatants were examined through ELISA assays. (**K–O**) Wild-type mice were intraperitoneally challenged with poly(I:C) at a dose of 2 mg/kg body weight for 6 hr and then intranasally infected with 1 × 10^9^ cfu of ΔRetS PAO1 and administrated with 20 μg TAT sequence containing VgrG2b C-terminus peptide, followed by survival calculation at the indicated days (**K**). Serum protein levels of IL-1β (**L**), TNF-α (**M**), and IL-6 (**N**) were examined through ELISA assays 24 hr post infection. Bacterial load was examined in mouse lung tissues 4 days post infection (**O**). Data were shown as means ± SD. Experiments were repeated three times with similar results. Figure 6—source data 1.File containing labeled original western blots for Figure 6. Figure 6—source data 2.Original gel image files for western blot analysis displayed in Figure 6. Figure 6—source data 3.Original source data for graphs displayed in Figure 6, Figure 6—figure supplement 1.

Finally, we infected mice with a *P. aeruginosa* strain carrying a D883A mutation in the VgrG2b gene. The survival rate of mice was greatly improved when infected with the mutant strain (Figure 6A). the level of IL-1β in the serum was also increased (Figure 6B), while other inflammatory cytokines such as TNF-α and IL-6 showed little difference among wild-type and mutant bacteria (Figure 6C, D). The bacterial load in the lungs of mice infected with the mutant strain was also decreased (Figure 6E). We also detected the activation of inflammasomes in macrophages from lung lavage fluid and found that the activation of the NLRP3 inflammasome was activated in cells infected with the mutant strain (Figure 6F), but the cell viability was not affected (Figure 6G). We detected the protein levels of cytokines in the lavage fluid and found that the level of IL-1β was increased from mice infected with *P. aeruginosa* mutant strain (Figure 6H). Other inflammatory cytokines such as TNF-α and IL-6 showed little difference (Figure 6I, J).

**Figure 6—figure supplement 1.**
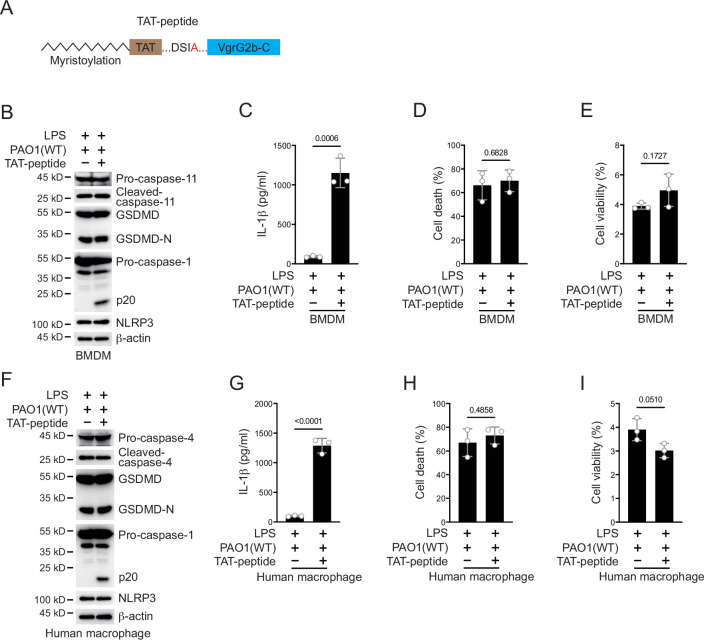
Activation of NLRP3 inflammasome ameliorates *P. aeruginosa* infection. (**A**) Scheme for design of a VgrG2b C-terminus peptide. VgrG2b C-terminus was added with an N-terminal TAT sequence, followed by myristoylation. (**B–E**) BMDM cells were primed overnight with 1 μg/ml Pam3CSK4, followed by transfection of 2 μg/ml lipopolysaccharide (LPS) using DOTAP with or without the incubation of ΔRetS PAO1 at a multiplicity of infection (MOI) of 30 and 2 μg/ml TAT-peptide for 2 hr. Cells were then supplemented with fresh medium containing 100 μg/ml Gentamycin. Cells were lysed and immunoblotted as indicated 16 hr post infection (**B**). Cell culture supernatants were collected for an ELISA assay to determine the secreted IL-1β protein levels 16 hr post infection (**C**). Cytotoxicity was determined by lactate dehydrogenase (LDH) release assay in cell culture supernatants 16 hr post infection (**D**). Cell viability was determined by an ATP quantification assay in cell pellets 16 hr post infection (**E**). (**F–I**) Human macrophages were primed overnight with 1 μg/ml Pam3CSK4, followed by transfection of 2 μg/ml LPS using DOTAP with or without the incubation of ΔRetS PAO1 at an MOI of 30 and 2 μg/ml TAT-peptide for 2 hr. Cells were then supplemented with fresh medium containing 100 μg/ml Gentamycin. Cells were lysed and immunoblotted as indicated 16 hr post infection (**F**). Cell culture supernatants were collected for an ELISA assay to determine the secreted IL-1β protein levels 16 hr post infection (**G**). Cytotoxicity was determined by LDH release assay in cell culture supernatants 16 hr post infection (**H**). Cell viability was determined by an ATP quantification assay in cell pellets 16 hr post infection (**I**). Data were shown as means ± SD. For C–E, G–I, data of three independent experiments were calculated. Experiments were repeated three times with similar results. Figure 6—figure supplement 1—source data 1.File containing labeled original western blots for Figure 6—figure supplement 1. Figure 6—figure supplement 1—source data 2.Original gel image files for western blot analysis displayed in Figure 6—figure supplement 1.

We designed a short peptide of VgrG2b C-terminus containing the VgrG2b linker region with a D883A substitution and a transmembrane TAT sequence. Peptides were then myristoylated (Zuber et al., 1989), aiming to target this peptide to inner membrane and suppress bacterial VgrG2b cleavage through competing with wild-type VgrG2b for caspase-11 (Figure 6—figure supplement 1A). We first conducted tests at the cellular level and found that adding this peptide to BMDM cells after incubation with *P. aeruginosa* relieved the inhibition of the NLRP3 inflammasome (Figure 6—figure supplement 1B) and the secretion of IL-1β was significantly increased (Figure 6—figure supplement 1C). The cell death rate did not change significantly and the viability rate was not affected by this peptide (Figure 6—figure supplement 1D, E). The same effect was observed in human macrophages, where this designed peptide could relieve the inhibition of *P. aeruginosa* on the NLRP3 inflammasome (Figure 6—figure supplement 1F) and the secretion of cytokine IL-1β was also increased (Figure 6—figure supplement 1G). Although it had no effect on cell death and viability (Figure 6—figure supplement 1H, I), Finally, we delivered this peptide into the lungs of mice infected with *P. aeruginosa* through nebulization, and found that the survival rate of mice was increased (Figure 6K). The level of IL-1β in the serum was increased (Figure 6L), while other inflammatory factors such as TNF-α and IL-6 remained unchanged (Figure 6M, N). The bacterial load was also decreased (Figure 6O), indicating the potential medicinal value of this designed peptide in the future clinical treatment of *P. aeruginosa* infections.

## Discussion

In this study, we found that the structural protein VgrG2b from *P. aeruginosa* can be cleaved by caspase-11 in host cells. The C-terminal fragment of VgrG2b bound to NLRP3, inhibiting the activation of the NLRP3 inflammatory and the secretion of IL-1β. The delivery of VgrG2b C-terminus mimics significantly improved the infection symptoms caused by *P. aeruginosa*, providing an optional treatment approach for chronic infections of *P. aeruginosa*.

The infection of *P. aeruginosa*, especially those in chronic infections, is an important factor causing pulmonary fibrosis in clinicals (Qin et al., 2022; Rossi et al., 2021). LPS from Gram-negative bacterium can be transported to host cells and activate non-canonical inflammasomes within them (Kayagaki et al., 2013; Shi et al., 2014). There are many ways for LPS from bacteria to be transported into host cells, including binding with HMGB1 originated from the liver and entering the interior of the cells through the surface of macrophages, as well as enveloping and delivering LPS into the host cells through extracellular vesicles (Deng et al., 2018; Vanaja et al., 2016). We observed the activation of non-canonical inflammasomes in macrophages after infection with *P. aeruginosa*, indicating that LPS was also transported into the cells. *P. aeruginosa* is not a typical intracellular bacterium, and its LPS entry into target cells is likely through the out membrane vesicles in vivo.

*P. aeruginosa* mainly utilizes the T6SS secretion system to promote the establishment of chronic infections (Qin et al., 2022). During this process, H2-T6SS plays many important roles, responsible for delivering known and yet to be explored virulence factors into host cells (Jiang et al., 2014; Sana et al., 2015). Here, we have found that VgrG2b is not only responsible for the delivery of bacterial virulence proteins, but it can also serve as a virulence factor on its own. Its uniqueness lies in the fact that it requires cutting to be effective. Whether there are other proteins secreted through the H2-T6SS in *P. aeruginosa* that need to be cleaved by host proteases before they exert their function is a very worthwhile research topic. Of course, this processing from the host is not limited to cleavage, but may also be post-translational modifications of proteins such as phosphorylation or ubiquitination (Ashida et al., 2014; Li et al., 2021). Systematically identifying these possible modifications and their substrates will be of great significance for understanding the pathogenic mechanism of *P. aeruginosa*.

The non-canonical inflammasome receptor caspase-11 has a very narrow substrate spectrum (Shi et al., 2023). The process of substrate cleavage by inflammatory caspases can be divided into two parts. Firstly, inflammatory caspases bind to a part of the substrate, which is often not their cleavage site, and the other is the recognition of the true site of their cleavage. This mechanism ensures that limited proteins are cleaved by host cells when inflammasomes are activated, thereby confining the severity of the inflammatory response. Here, we found that caspase-11/4 specifically cleaves the structural proteins of the secretion system of *P. aeruginosa*, and the generated new fragments can play an inhibitory role. This is a new way for pathogenic microorganisms to hijack the host’s immune system. Because traditional hijacking usually directly affects the host immune system without cutting. It is very effective for *P. aeruginosa* using VgrG2b fragment to inhibit NLRP3. On the one hand, VgrG2b transports virulence factors into the host cell, and on the other hand, the cleaved substrate plays a role in inhibiting the NLRP3 inflammasome.

In summary, we have discovered a new pattern of recognition between bacterial proteins and the host immune system during *P. aeruginosa* infection, which involves hijacking the host’s protease to produce a new virulence factor that can inhibit the host’s immune response.

## Materials and methods

### Antibodies and reagents

Antibodies against NLRP3 (15101), IL-1β (12703, 12426), cleaved IL-1β (83186, 63124), GSDMD (93709), caspase-1 (3866, 24232, 2225), and ASC (13833, 67824) were purchased from Cell Signaling Technology. Antibodies against caspase-1 (ab207802), NEK7 (ab133514), Nur77 (ab153914), and GSDMD (ab215203) were purchased from Abcam. Antibodies against caspase-11 were: ab180673 from Abcam, 14340 from Cell Signaling Technology, 14-9935-82 from Invitrogen, and M029-3 from MBL Beijing Biotech. Anti-c-Myc magnetic beads (88843) were purchased from Pierce. Antibody against 6xHis tag (MA121315) was purchased from Invitrogen. Antibodies against FLAG tag (F3165), β-actin (A1978), and anti-FLAG M2 magnetic beads (M8823) were purchased from Sigma-Aldrich. Antibodies against c-Myc (sc-40), Tom20 (sc-11415), GAPDH (sc-32233), and GST (sc-138) were purchased from Santa Cruz Biotechnology. Anti-HA tag antibody (HX1820) was purchased from Huaxingbio (Beijing). HRP-conjugated goat anti-mouse IgG (SA00001-1) and HRP-conjugated goat anti-rabbit IgG (SA00001-2) were purchased from Proteintech Group. Alexa Fluor Plus 488-conjugated goat anti-mouse IgG (A32723) and Alexa Fluor 594-conjugated goat anti-rabbit IgG (A32740) were purchased from Invitrogen. Alexa Fluor Plus 488-conjugated donkey anti-goat IgG (bs-0294D-AF488) and Alexa Fluor 594-conjugated donkey anti-rabbit IgG (bs-0295D-AF594) were purchased from Bioss (Beijing).

Glutathione sepharose 4B resin (17075601) was purchased from Cytiva. Protein A/G PLUS-Agarose beads (sc-2003) were purchased from Santa Cruz Biotechnology. DAPI (2879038) was purchased from PeproTech (BioGems). SMART MMLV reverse transcriptase (639524) was purchased from Clontech, Takara Bio. Random primer (Hexadeoxyribonucleotide mixture, pd(N)6) (3801), X-α-Gal (630462), and carrier DNA (630440) were purchased from Takara Bio. Protease inhibitor cocktail (11697498001) was purchased from Roche. Total RNA extraction kit (8034111) was purchased from Dakewe (Beijing). Ni-NTA agarose beads (R90115) and AminoLink coupling resin (20381) were purchased from Invitrogen. LPS (B46894) was purchased from Innochem (Beijing). DOTAP was: D10530 from Psaitong (Beijing) and 11202375001 from Roche. Nigericin (481990), Gramicidin (368020), and ATP (A6559) were purchased from Merck Millipore. Poly(I:C) (tlrl-picw) and Pam3CSK4 (tlrl-pms) were purchased from InvivoGen. Cell-penetrating peptides: ab142343 from Abcam and sc-396807 from Santa Cruz. Human M-CSF (300-25) and murine M-CSF (315-02) were purchased from PeproTech. CellTiter-Glo luminescent cell viability assay kit (G7570) and CytoTox 96 non-radioactive cytotoxicity assay kit (G1780) were purchased from Promega.

### Bacteria and CFU determination

*E. coli* (CGMCC1.2389, ATCC11775) was from China General Microbiological Culture Collection Center. *Pseudomonas aeruginosa* (PAO1) was a gift from Dr. Lvyan Ma (Institute of Microbiology, Chinese Academy of Sciences). *E. coli* and *P. aeruginosa* were preserved in LB plates and inoculated to liquid LB medium, followed by shaking at 37°C until they grew to the exponential phase (optical density = 0.6–0.8). Bacteria were diluted and spread into LB plates to determine the CFU numbers. When the bacteria were cultured to the mid-exponential stage, they were washed three times with PBS and resuspended in PBS or DMEM.

### *P. aeruginosa* OMV purification

When *P. aeruginosa* was cultured to the mid-exponential stage, cultures were centrifugated to obtain the supernatant. After filtering through a 0.22 μm filter, the supernatant was placed in a discontinuous sucrose gradient tube and centrifuged at a speed of 100,000 × *g* for 16 hr. The precipitate was resuspended by PBS and centrifuged again to remove residual culture medium components. The precipitate was resuspended with PBS again, resulting in the OMV sample. The sample was immediately used or frozen and stored in a −80°C freezer. 20 μg/ml OMV was used in each in vitro experiment.

### *P. aeruginosa* knockout and knockin strategy

The knockout plasmid pK18mobsacBGm was digested with BamHI and HindIII, and then recovered by agarose gel electrophoresis. Primers were designed to clone homologous arms of approximately 900 bp upstream and downstream of the target gene. This knockout strategy preserved the starting 99 bp and ending 99 bp of the target gene. The upstream and downstream homologous arms were then connected with the linearized pk18mobsacBGm vector using homologous recombination, and the knockout vector pk18mobsacBGm-target were obtained through sequencing. Primers used were: RetS-up-F: 5′-ctcggtacccggggatccccgccgtgcgcgacatgctcgccggcaa-3′, RetS-up-R: 5′-agcacgtcgctgc ccggcgaagtcccttcg-3′, RetS-down-F: 5′-ccttcgaagggacttcgccgggcagcgacgtgctccgg-3′, RetS-down-R: 5′-cgttgtaaaacgacggccagtgccaagcttcgagggtcaggcaggcgag-3′ for RetS knockout; KO-VgrG2b-up-F: 5′-attcgagctcggtacccggggatcccttgatggaaaagagtttcaagacc-3′, KO-VgrG2b-up-R: 5′-tctcgaggaaataatctcgaacgataggctcgcagagcgcttcttccagt-3′, KO-VgrG2b-down-F: 5′-actggaagaagcgctctgcgagcctatcgttcgagattatttcctcga-3′, KO-VgrG2b-down-R: 5′-taaaacgacggccagtgccaagcttgacgacgtcggggttctctgcctt-3′ for VgrG2b knockout; KI-Myc-VgrG2b-up-F: 5′-tcgagctcggtacccggggatccgagcacatcaccctgatgtgcggcggcgcct-3′, KI-Myc-VgrG2b-up-R1: 5′-ttcgaaccgcgggccctctagactcgagcggtatcccgttgggaagtttttcagt-3′, KI-Myc-VgrG2b-up-R2: 5′-atcctcttctgagatgagtttttgttcgaaccgcgggccctctagactc-3′, KI-Myc-VgrG2b-down-F: 5′-aaaaactcatctcagaagaggatctgtgaccaatgaaatgcaagaccttgctca-3′, KI-Myc-VgrG2b-down-R: 5′-taaaacgacggccagtgccaagcttcggccccgccggcgccactggcgaa-3′ for VgrG2b-Myc knockin; VgrG2b-D883A-up-F: 5′-aattcgagctcggtacccggggatccgagcaccagg gcgtggggcacgacg-3′, VgrG2b-D883A-up-R: 5′-aggtcttgcatttcattggtcacagatcctcttctgagat gag-3′, VgrG2b-D883A-down-F: 5′-actcatctcagaagaggatctgtgaccaatgaaatgcaagaccttgctc a-3′, VgrG2b-D883A-down-R: 5′-taaaacgacggccagtgccaagcttttcgccggccaggcagaattcgacg-3′ for VgrG2b D883A knockin. The recipient bacterium *P. aeruginosa*, co-plasmid pRK2013, and donor bacterium (pK18mobsacBGm-target) were inoculated in LB liquid medium (with corresponding antibiotics added), and incubated overnight in a shaking table at 37°C and 200 rpm. 1 ml of co-plasmid and donor bacterial culture medium were collected separately and centrifuged at 5000 rpm for 2 min. Pellets were collected and resuspended in 200 μl LB medium. Co-plasmids and donor bacteria were mixed in a 1:1 ratio, and inoculated on LB solid culture medium at 37°C for 2 hr. Simultaneously, the recipient strain *P. aeruginosa* was placed in 42°C for 2 hr of cultivation. 1 ml of receptor bacteria was collected and centrifuged at 5000 rpm for 2 min and resuspended in 200 μl LB liquid medium. The bacteria were spotted onto a mixed plaque of co-plasmids and donor bacteria and cultured at 37°C for 4 hr. The plaque was picked up and suspended in the 500–800 μl LB liquid medium and plated onto LB plates with Irg and Gm resistance, followed by culture overnight at 37°C. A single colony was selected and transferred again to LB plates with Irg and Gm resistance. The next day, bacteria were drawn a line on a 20–22% sucrose agar plate and cultured at 37°C for 24–48 hr. Single colonies were selected again according to the above method. Colonies that could grow on LB plates but not on Gm-resistant plates were chosen and subjected to PCR validation.

### Cell component fractionation and mitochondrial DNA quantification

Cells were fractionated according to the guidelines of a cell fractionation kit from Abcam (ab109719). Mitochondria were isolated from macrophages using a mitochondrial isolation kit from Abcam (ab110170) or from Invitrogen (89874), followed by mitochondrial DNA purification through a mitochondrial DNA isolation kit from Abcam (ab65321). Total cytosolic DNA was extracted using a DNeasy Blood & Tissue Kit (QIAGEN) following the manufacturer’s instructions. For mitochondrial DNA quantification, samples containing mitochondrial DNA were subjected to PCR analyses using the following primers: 5′-GAGATGACCAAATTTACAATG-3′ (forward) and 5′-TCCTGTTCCTGCTCCTGCTTC-3′ (reverse) for mouse mitochondrial gene *mt-Co1*.

### Mice and infection

*Casp11*^–/–^ (S-KO-01332), *Gsdmd*^–/–^ (S-KO-12963), and *Aim2*^–/–^ (S-KO-09889) mice were purchased from Cyagen Biosciences (Jiangsu, China). Mice were intraperitoneally challenged with poly(I:C) at a dose of 2 mg/kg body weight for 6 hr and then intranasally infected with 1 × 10^9^ cfu of *P. aeruginosa* strain PAO1, followed by survival calculation at the indicated days and bacterial load determination 4 days later. Sera were collected and subjected to ELISA assays. For bronchoalveolar lavage fluid (BALF) collection, mice were anaesthetized, followed by thoracic cavity opened and a flush of circulating blood cells by injecting about 10 ml ice-cold PBS into the right ventricle. Mouse neck was then dissected to expose trachea. A puncture needle was inserted into the upper end of the trachea, followed by repeated rinsing with 0.8 ml PBS for five times. BALF collections were centrifuged at 300 × *g* at 4°C for 5 min, followed by ELISA assays of the supernatants or macrophage isolation in the cell pellets. Study was licensed by the Ethics Committee of Peking University (BCJE0167).

### Primary cell separation and culture

To generate BMDM cells, bone marrow cells were flushed from mouse femurs and tibias, followed by red blood cell removal using red blood cell lysis buffer. Cells were resuspended in alpha-MEM containing 10% (vol/vol) fetal bovine serum and 10 ng/ml M-CSF and grown at 37°C with a 5% CO_2_ humidified atmosphere for 7 days. To produce human macrophages, human bloods collected from healthy donors were mixed with an equal volume of saline and then added to lymphocyte separation medium, followed by centrifugation and peripheral blood mononuclear cell (PBMC) collection. Human PBMCs were incubated with APC-conjugated anti-CD14 antibody on ice for 30 min, followed by incubation with anti-APC-conjugated magnetic beads. Monocytes were enriched on a magnetic separator. To test the purity of sorted monocytes, cells were stained with APC-conjugated anti-CD14 antibody, followed by FACS examination. Cells with purity above 95% were used for subsequent experiments. Cells were resuspended in RPMI-1640 medium containing 10% (vol/vol) fetal bovine serum and 50 ng/ml human M-CSF, followed by growing at 37°C with a 5% CO_2_ humidified atmosphere for 7 days. Healthy blood samples were provided by the researchers volunteering. Informed consents were obtained from all subjects and experiments conformed to related principles. Study was licensed by the Ethics Committee of Peking University (PUIRB-YS2023204).

### Inflammasome induction

For canonical NLRP3 inflammasome activation, cells were incubated with 1 μg/ml LPS for 3 hr, followed by stimulation with 10 μM nigericin for 30 min, 0.5 μM gramicidin for 1 hr and 2 mM ATP for 30 min. Cells were grown at 37°C with a 5% CO_2_ humidified atmosphere. For non-canonical inflammasome stimulation, macrophages were primed overnight with 1 μg/ml Pam3CSK4, followed by transfection of 2 μg/ml LPS using DOTAP for 16 hr. Otherwise, activation induced by *P. aeruginosa* infection, bacteria were cultured in LB at 37°C until OD_600_ reached 0.8, then washed by PBS and diluted in serum-free DMEM medium to infect cells. Macrophages were primed overnight with 1 μg/ml Pam3CSK4 and incubated with *P. aeruginosa* strain PAO1 at an MOI of 30 and OMVs for 2 hr. Cells were then supplemented with fresh medium containing 100 μg/ml Gentamycin and cultured for 16 hr.

### Plasmid transfection and lentiviral production

Transfection reagent JetPRIME (Polyplus Transfection) was used to conduct transfection in HEK293T cells. HEK293T cells were plated 14 hr earlier to achieve a density of about 70% when transfection was performed. Lentiviral plasmids containing exogenous genes of interest and packaging plasmids pSPAX2 and pMD2.G were transfected into HEK293T cells at a ratio of 4:3:1. Forty-eight hours later, supernatants were collected, followed by centrifugation at 1000 rpm for 5 min and filtration using 0.45-μm sterile syringe filters (Merck Millipore). The filtrate was then concentrated with a 100-kD Amicon ultra centrifugal filter unit (Merck Millipore). Macrophage cells were incubated with lentiviruses for 6 hr, followed by culture medium replacement with fresh medium. 2 μg/ml puromycin was added to the medium 48 hr post infection for 3 days.

### Protein transfection using cell-penetrating peptides

For a single well of 6-well plate macrophages, 10 μg of wild-type or mutant VgrG2b-C (884–1019) was incubated with 10 μg cell-penetrating peptides in 100 μl of PBS at room temperature for 30 min, followed by addition of the peptide–cell-penetrating peptides mixture to the cell medium. The culture medium was replaced with fresh medium later.

### Cell viability and cytotoxicity assay

Cells following inflammasome activation were centrifuged at 187 × *g* for 5 min, and supernatants and cell pellets were collected, respectively. Cell pellets from cells stimulated with inflammasome activators were used to measure cell viability through checking cellular ATP levels with a CellTiter-Glo luminescent cell viability assay kit (Promega). The supernatants from cells stimulated with inflammasome activators were collected and subjected to lactate dehydrogenase (LDH) assay to assess cell death using a CytoTox 96 non-radioactive cytotoxicity assay kit (Promega) and cellular cytotoxicity was displayed as a percentage of total cellular LDH (100% cell lysis control). Both the supernatants and cell pellets were collected for immunoblotting analyses.

### ELISA

ELISA kits detecting human IL-1β (D711068), mouse IL-1β (D721017), mouse IL-6 (D721022), and mouse TNF-α (D721217) were purchased from BBI life sciences (Shanghai). Cell culture supernatants were collected and subjected to ELISA assays following manufacturer’s instructions.

### RNA extraction and RT-PCR

A total RNA extraction kit (Dakewe, Beijing) was used to extract RNA from cells according to the manufacturer’s instructions. For reverse transcription, 1 μg RNA was mixed with 2 μl of N6 primers and 7 μl of DEPC water, followed by incubation at 72°C for 2 min. The mixture was then chilled on ice for 2 min and mixed with 4 μl of 5× first-strand buffer (TaKaRa Bio), 2 μl of 100 mM DTT, 2 μl of 10 mM dNTP mix (Solarbio, Beijing), and 2 μl of SMART MMLV reverse transcriptase (Clontech, TaKaRa Bio). The mixture was incubated under the following conditions: at 25°C for 10 min, at 42°C for 1 hr, and at 75°C for 10 min. cDNA samples were diluted for further PCR analysis.

### Recombinant protein expression and purification

For recombinant expression of PAO1 VgrG2b (full-length and C-terminus), cDNA of full-length PAO1 VgrG2b, amino acids (aa) 884–1019, and mutant (H935A;H936A;H939A;E983A) aa 884–1019 was cloned into pGEX-6P-1 vector with an N terminal GST tag and a C terminal Myc tag. Plasmids were transformed into *E. coli* strain BL21 and the bacteria grew in LB medium supplemented with 100 μg/ml ampicillin at 37°C for 8 hr, followed by addition of isopropyl β-D-1-thiogalactopyranoside (IPTG) to a final concentration of 0.5 mM after OD_600_ reached 0.8 and shaking at 150 rpm at 37°C for 3 hr. Cells were harvested and resuspended with PBS containing 1% Triton X-100 and lysed by sonication. Cell lysates were centrifuged at 13,000 rpm at 4°C for 30 min and filtered through sterile syringe filters. For crude purification of recombinant proteins, the filtered lysate was loaded on column containing glutathione sepharose 4B resin, followed by washing with PBS containing 1% Triton X-100. The protein was eventually eluted by 1 ml 10 mM L-glutathione solution. For removal of LPS contaminations, crudely purified proteins were further eluted through Pierce high-capacity endotoxin removal spin columns and heparin columns to remove LPS and bacterial genomic DNA, respectively. GST tags were removed by a PreScission protease.

For recombinant expression of caspase-11 p10 and p22, cDNAs encoding proteins aforementioned were cloned into pET-15b with a C-terminal 6xHis tag. Plasmids were transformed into *E. coli* strain BL21, and the bacteria grew in LB medium supplemented with 100 μg/ml ampicillin for 8 hr at 37°C. Protein expression was induced by 0.1 mM IPTG through shaking at 120 rpm overnight at 16°C. The whole purification procedure was performed at 4°C. Cells were collected and resuspended in a binding buffer containing 0.5 M NaCl, 20 mM Tris, 5 mM imidazole, and 1% Triton X-100, pH 7.5, and lysed by sonication. The lysate was centrifuged at 16,200 × *g* for 30 min. Cleared lysate was filtered through sterile syringe filters before passing through a column loaded with Ni-NTA agarose beads. The column was washed with a wash buffer containing 0.5 M NaCl, 20 mM Tris, and 60 mM imidazole, pH 7.5, and eluted by an elution buffer containing 1 M NaCl, 40 mM Tris, and 2 M imidazole, pH 7.5.

### In vitro cleavage assay

In vitro caspase cleavage was performed as previously described (Wang et al., 2020). Hydrolysis of VgrG2b substrates by recombinant caspases was performed in a buffer containing 50 mM HEPES (pH 7.5), 150 mM NaCl, 3 mM EDTA, and 0.005% (vol/vol) CHAPS, and 10 mM DTT. 1 μg recombinant VgrG2b protein with or without inhibitor were incubated with 0.5 mM caspase-11 p22/p10 complex in a 40-μl reaction buffer at 37°C for 4 hr. The mixture was added with SDS loading buffer and boiled for 10 min, followed by standard immunoblotting analysis.

### Immunoprecipitation

HEK293T cells were seeded on 6-well culture plates at a confluency of 70% and were transfected with plasmids 16 hr later. Twenty-four hours post transfection, cells were washed twice with PBS and lysed in a pre-chilled buffer containing 150 mM NaCl, 50 mM Tris (pH 7.5), 1 mM EDTA, 0.5% digitonin, 1% protease inhibitor cocktail and 10% glycerol on ice for 30 min. For immunoprecipitation using primary cells, cells post stimulation or treatments were lysed in a pre-chilled PBS buffer containing 0.5% digitonin, 1% protease inhibitor cocktail on ice for 30 min. Cell lysates were centrifuged at 13,000 rpm at 4°C for 10 min. Primary antibodies and their isotype control IgG were immobilized to AminoLink Coupling Resin (ThermoFisher Scientific) following the manufacturer’s instructions. Supernatants were incubated with immobilized antibodies at 4°C for 2 hr. Resins were washed three times with PBS containing 0.5% digitonin, followed by immunoprecipitant detachment in 0.1 M glycine-HCl, pH 2.7. Supernatants were neutralized by adding 1/10 the volume of 2 M Tris-HCl, pH 8.0, followed by immunoblotting with the indicated antibodies.

### Yeast two-hybrid screening

Yeast strain AH109 was inoculated into 5 ml YPDA medium and shook at 250 rpm for 16 hr at 30°C. The overnight culture was transferred to a flask containing 50 ml YPDA and incubated for 3 hr at 30°C. Yeast cells were collected by centrifugation at 3000 × *g* for 10 min and resuspended in a 1× TE/LiAc buffer (pH7.5) containing 0.1 M lithium acetate, 0.1 M Tris-HCl, and 10 mM EDTA. A mixture of plasmid DNA and carrier DNA was added to the yeast cell suspension and vortexed to mix well. A PEG/LiAc buffer containing 40% polyethylene glycol 3350, 0.1 M lithium acetate, 0.1 M Tris-HCl, and 10 mM EDTA was then added to the suspension and vortexed at a high speed. The suspension was shaken at 200 rpm for 30 min at 30°C, followed by addition of DMSO and heat shock for 15 min in a 42°C water bath. Cells were chilled in an ice bath for 2 min and resuspended to plate on appropriate SD agar plates. A mouse bone marrow cDNA library was used for Y2H screening with BD-tagged VgrG2b-C as bait. BD-tagged VgrG2b-C truncation and AD-tagged NLRP3, NLRP6, and NLRC4 were co-transfected into yeast strain AH109, and double-positive clones were grown on plates containing X-α-gal. The intensity of blue color was monitored on a scanner.

### Immunofluorescence

Cells adhered to covers pre-coated with poly-L-lysine were stimulated with inflammasome activators, followed by fixation with 4% paraformaldehyde for 10 min at room temperature. Cells were then washed twice with PBS and permeabilized with PBS containing 0.1% digitonin for 10 min, followed by washing with PBS for twice and blockage in 10% normal goat serum at 37°C for 30 min. Cells were incubated with primary antibody for 2 hr, followed by washing with PBS for three times and further incubation with fluorescence-conjugated secondary antibody for 1 hr. Nuclei were stained with DAPI. Cells were visualized through an UltraVIEW VoX imaging system (PerkinElmer).

### Statistical analysis

No statistical methods were used to predetermine sample size. Experiments were independently repeated at least three times to achieve statistical significance. No randomization or blinding procedures was used in this study. No samples were excluded from the analysis. Data were shown as means ± SD of three technical replicates. Data with normal distribution determined by Shapiro–Wilk normality test were statistically analyzed by two tailed Student’s *t*-tests if not specified. The Gehan–Breslow–Wilcoxon test was used for the analysis of survival data. Data were analyzed by GraphPad Prism 9.0. p-values ≤0.05 were termed as significant; NS means non-significant where p > 0.05.

## Data Availability

All data generated or analyzed during this study are included in the manuscript and supporting files.

## Additional files

MDAR checklist

## Appendix 1

**Appendix 1—key resources table:**

Reagent type (species) or resource	Designation	Source or reference	Identifiers	Additional information
Strain, strain background (*Mus musculus*)	*Casp11*^–/–^ mice	Cyagen Biosciences (Jiangsu, China)	S-KO-01332	
Strain, strain background (*Mus musculus*)	*Gsdmd* ^–/–^	Cyagen Biosciences (Jiangsu, China)	S-KO-12963	
Strain, strain background (*Mus musculus*)	*Aim2* ^–/–^	Cyagen Biosciences (Jiangsu, China)	S-KO-09889	
Strain, strain background (*Escherichia coli*)	*E. coli*	China General Microbiological Culture Collection Center	ATCC11775	
Strain, strain background (*Pseudomonas aeruginosa*)	PAO1	Institute of Microbiology, Chinese Academy of Sciences	CGMCC1.12483	
Antibody	anti-NLRP3 (Rabbit monoclonal)	Cell Signaling Technology	Cat# 15101	(1:1000)
Antibody	anti-IL-1β (Rabbit monoclonal)	Cell Signaling Technology	Cat# 12703	(1:1000)
Antibody	anti-IL-1β (Rabbit monoclonal)	Cell Signaling Technology	Cat# 12426	(1:1000)
Antibody	anti-cleaved IL-1β (Rabbit monoclonal)	Cell Signaling Technology	Cat# 83186	(1:1000)
Antibody	anti-cleaved IL-1β (Rabbit monoclonal)	Cell Signaling Technology	Cat# 63124	(1:1000)
Antibody	anti-GSDMD (Rabbit polyclonal)	Cell Signaling Technology	Cat# 93709	(1:1000)
Antibody	anti- caspase-1 (Rabbit monoclonal)	Cell Signaling Technology	Cat# 3866	(1:1000)
Antibody	anti-caspase-1 (Rabbit monoclonal)	Cell Signaling Technology	Cat# 24232	(1:1000)
Antibody	anti-caspase-1 (Rabbit polyclonal)	Cell Signaling Technology	Cat# 2225	(1:1000)
Antibody	anti-ASC (Rabbit monoclonal)	Cell Signaling Technology	Cat# 13833	(1:1000)
Antibody	anti-ASC (Rabbit monoclonal)	Cell Signaling Technology	Cat# 67824	(1:1000)
Antibody	anti-caspase-1 (Rabbit monoclonal)	Abcam	Cat# ab207802	(1:1000)
Antibody	anti-NEK7 (Rabbit monoclonal)	Abcam	Cat# ab133514	(1:10,000)
Antibody	anti-Nur77 (Rabbit polyclonal)	Abcam	Cat# ab153914	(1:1000)
Antibody	anti-GSDMD (Rabbit monoclonal)	Abcam	Cat# ab215203	(1:1000)
Antibody	anti-caspase-11 (Rabbit monoclonal)	Abcam	Cat# ab180673	(1:1000)
Antibody	anti-caspase-11 (Rat monoclonal)	Cell Signaling Technology	Cat# 14340	(1:1000)
Antibody	anti-caspase-11 (Rat monoclonal)	Invitrogen	Cat# 14-9935-82	(1:1000)
Antibody	anti-caspase-11 (Mouse monoclonal)	MBL Beijing Biotech	Cat# M029-3	(1:1000)
Antibody	anti-6xHis tag (Mouse monoclonal)	Invitrogen	Cat# MA121315	(1:1000)
Antibody	anti-FLAG tag (Mouse monoclonal)	Sigma-Aldrich	Cat# F3165	(1:1000)
Antibody	anti-β-actin (Mouse monoclonal)	Sigma-Aldrich	Cat# A1978	(1:2000)
Antibody	anti-c-Myc (Mouse monoclonal)	Santa Cruz Biotechnology	Cat# sc-40	(1:1000)
Antibody	anti-Tom20 (Rabbit polyclonal)	Santa Cruz Biotechnology	Cat# sc-11415	(1:500)
Antibody	anti-GAPDH (Mouse monoclonal)	Santa Cruz Biotechnology	Cat# sc-32233	(1:1000)
Antibody	anti-HA tag (Rabbit polyclonal)	Huaxingbio (Beijing)	Cat# HX1820	(1:5000)
Antibody	HRP-conjugated anti-mouse IgG (Goat polyclonal)	Proteintech	Cat# SA00001-1	(1:5000)
Antibody	HRP-conjugated goat anti-rabbit IgG (Goat polyclonal)	Proteintech	Cat# SA00001-2	(1:5000)
Sequence-based reagent	RetS-up-F	This paper	PCR primers	ctcggtacccggggatccccgc cgtgcgcgacatgctcgccggcaa
Sequence-based reagent	RetS-up-R	This paper	PCR primers	agcacgtcgctgccc ggcgaagtcccttcg
Sequence-based reagent	RetS-down-F	This paper	PCR primers	ccttcgaagggacttcgccg ggcagcgacgtgctccgg
Sequence-based reagent	RetS-down-R	This paper	PCR primers	cgttgtaaaacgacggccagtgcca agcttcgagggtcaggcaggcgag
Sequence-based reagent	KO-VgrG2b-up-F	This paper	PCR primers	attcgagctcggtacccggggatcc cttgatggaaaagagtttcaagacc
Sequence-based reagent	KO-VgrG2b-up-R	This paper	PCR primers	tctcgaggaaataatctcgaacg ataggctcgcagagcgcttcttccagt
Sequence-based reagent	KO-VgrG2b-down-F	This paper	PCR primers	actggaagaagcgctctgcgag cctatcgttcgagattatttcctcga
Sequence-based reagent	KO-VgrG2b-down-R	This paper	PCR primers	taaaacgacggccagtgccaagc ttgacgacgtcggggttctctgcctt
Sequence-based reagent	KI-Myc-VgrG2b-up-F	This paper	PCR primers	tcgagctcggtacccggggatccgag cacatcaccctgatgtgcggcggcgcct
Sequence-based reagent	KI-Myc-VgrG2b-up-R1	This paper	PCR primers	ttcgaaccgcgggccctctagactcga gcggtatcccgttgggaagtttttcagt
Sequence-based reagent	KI-Myc-VgrG2b-up-R2	This paper	PCR primers	atcctcttctgagatgagtttttgttc gaaccgcgggccctctagactc
Sequence-based reagent	KI-Myc-VgrG2b-down-F	This paper	PCR primers	aaaaactcatctcagaagaggatctgt gaccaatgaaatgcaagaccttgctca
Sequence-based reagent	KI-Myc-VgrG2b-down-R	This paper	PCR primers	taaaacgacggccagtgccaagcttc ggccccgccggcgccactggcgaa
Sequence-based reagent	VgrG2b-D883A-up-F	This paper	PCR primers	aattcgagctcggtacccggggatcc gagcaccagggcgtggggcacgacg
Sequence-based reagent	VgrG2b-D883A-up-R	This paper	PCR primers	aggtcttgcatttcattggtcac agatcctcttctgagatgag
Sequence-based reagent	VgrG2b-D883A-down-F	This paper	PCR primers	actcatctcagaagaggatctgtgac caatgaaatgcaagaccttgctca
Sequence-based reagent	VgrG2b-D883A-down-R	This paper	PCR primers	taaaacgacggccagtgccaagct tttcgccggccaggcagaattcgacg
Commercial assay or kit	RNA extraction kit	Dakewe (Beijing)	Cat# 8034111	
Commercial assay or kit	CellTiter-Glo luminescent cell viability assay kit	Promega	Cat# G7570	
Commercial assay or kit	CytoTox 96 non-radioactive cytotoxicity assay kit	Promega	Cat# G1780	
Chemical compound, drug	LPS	Innochem (Beijing)	Cat# B46894	
Chemical compound, drug	DOTAP	Psaitong (Beijing)	Cat# D10530	
Chemical compound, drug	DOTAP	Roche	Cat# 11202375001	
Chemical compound, drug	Nigericin	Merck Millipore	Cat# 481990	
Chemical compound, drug	Gramicidin	Merck Millipore	Cat# 368020	
Chemical compound, drug	ATP	Merck Millipore	Cat# A6559	
Chemical compound, drug	Poly(I:C)	InvivoGen	Cat# tlrl-picw	
Chemical compound, drug	Pam3CSK4	InvivoGen	Cat# tlrl-pms	
Chemical compound, drug	Cell-penetrating peptides	Abcam	Cat# ab142343	
Chemical compound, drug	Cell-penetrating peptides	Santa Cruz	Cat# sc-396807	
Chemical compound, drug	Human M-CSF	PeproTech	Cat# 300-25	
Chemical compound, drug	Murine M-CSF	PeproTech	Cat# 315-02	
Software, algorithm	GraphPad Prism	GraphPad Software, https://www.graphpad.com/	RRID:SCR_002798	
