# Supplementary figures and images for: A VgrG2b fragment cleaved by caspase-11/4 promotes Pseudomonas aeruginosa infection through suppressing the NLRP3 inflammasome (part 1 of 4)
Source: eLife. 2025 Feb 25;13:RP99939. doi: 10.7554/eLife.99939 (PMC11856931; doi:10.7554/eLife.99939)

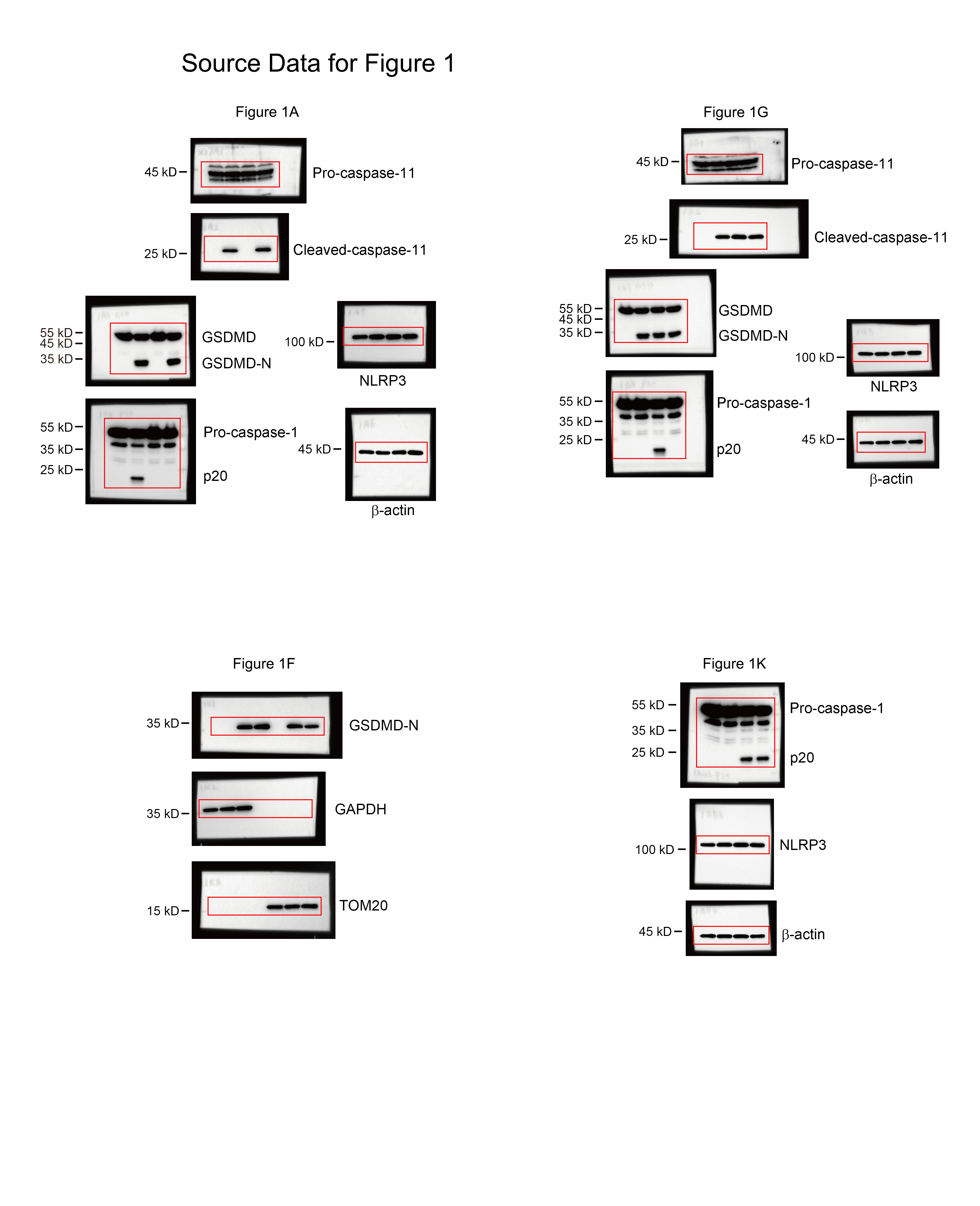

Supplement: Figure 1—source data 1. [file elife-99939-fig1-data1.zip › Figure 1-source data 1.tif]

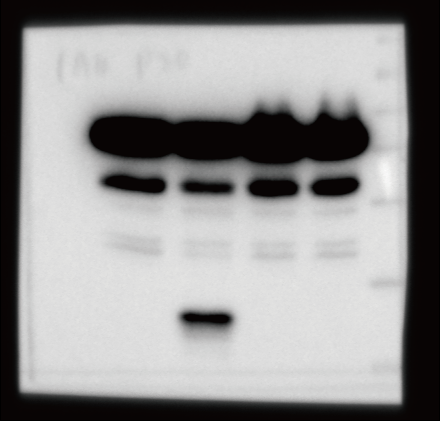

Supplement: Figure 1—source data 2. [file elife-99939-fig1-data2.zip › Figure 1-source data 2/Figure 1A caspase-1.tif]

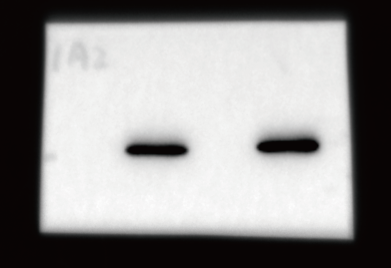

Supplement: Figure 1—source data 2. [file elife-99939-fig1-data2.zip › Figure 1-source data 2/Figure 1A cleaved-caspase-11.tif]

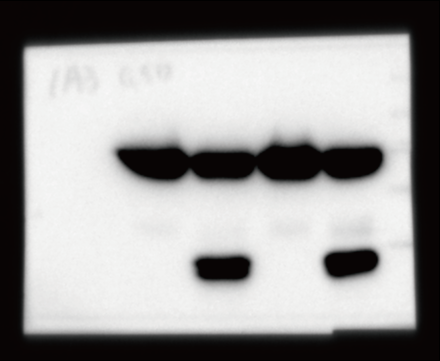

Supplement: Figure 1—source data 2. [file elife-99939-fig1-data2.zip › Figure 1-source data 2/Figure 1A GSDMD.tif]

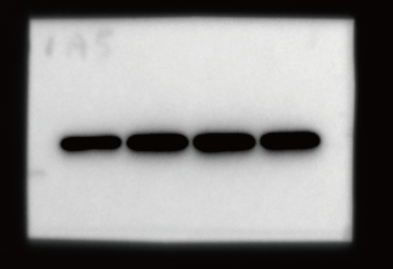

Supplement: Figure 1—source data 2. [file elife-99939-fig1-data2.zip › Figure 1-source data 2/Figure 1A NLRP3.tif]

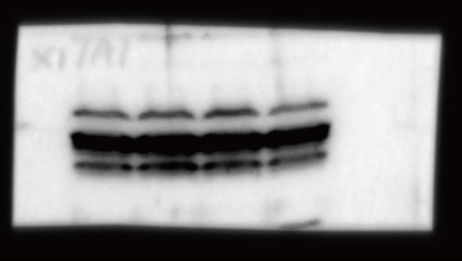

Supplement: Figure 1—source data 2. [file elife-99939-fig1-data2.zip › Figure 1-source data 2/Figure 1A pro-caspase-11.tif]

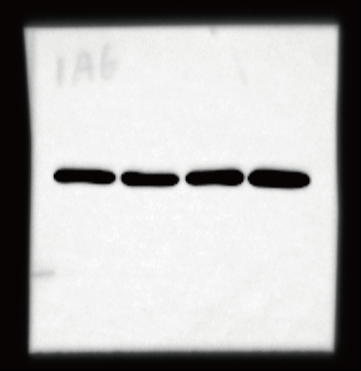

Supplement: Figure 1—source data 2. [file elife-99939-fig1-data2.zip › Figure 1-source data 2/Figure 1A β-actin.tif]

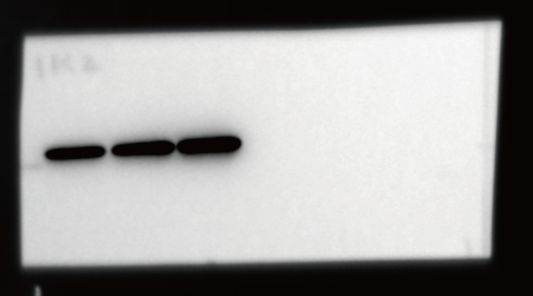

Supplement: Figure 1—source data 2. [file elife-99939-fig1-data2.zip › Figure 1-source data 2/Figure 1F GAPDH.tif]

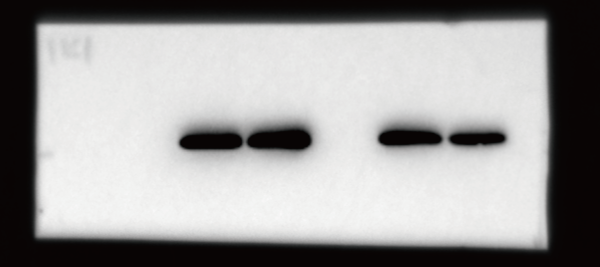

Supplement: Figure 1—source data 2. [file elife-99939-fig1-data2.zip › Figure 1-source data 2/Figure 1F GSDMD-N.tif]

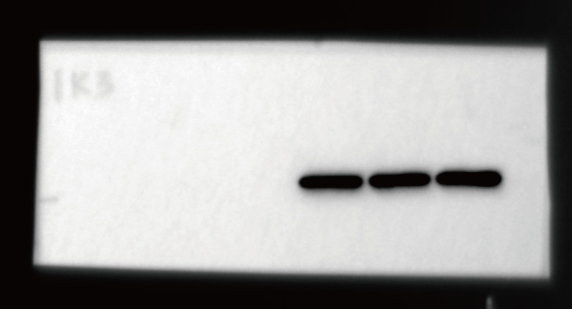

Supplement: Figure 1—source data 2. [file elife-99939-fig1-data2.zip › Figure 1-source data 2/Figure 1F TOM20.tif]

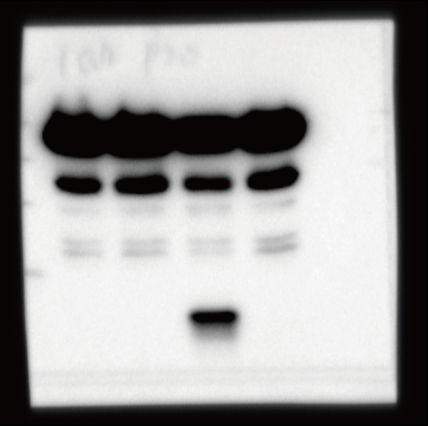

Supplement: Figure 1—source data 2. [file elife-99939-fig1-data2.zip › Figure 1-source data 2/Figure 1G caspase-1.tif]

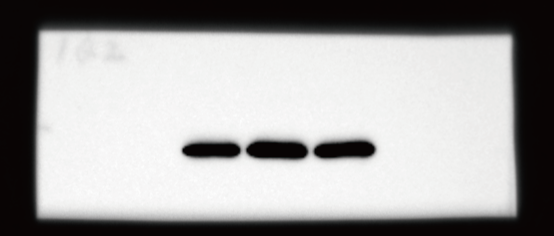

Supplement: Figure 1—source data 2. [file elife-99939-fig1-data2.zip › Figure 1-source data 2/Figure 1G cleaved-caspase-11.tif]

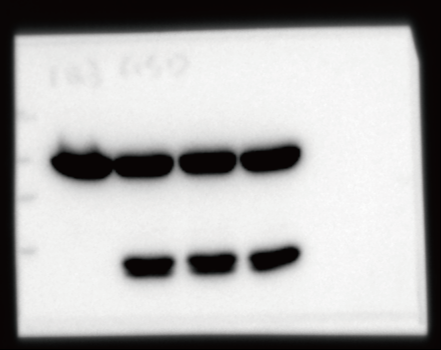

Supplement: Figure 1—source data 2. [file elife-99939-fig1-data2.zip › Figure 1-source data 2/Figure 1G GSDMD.tif]

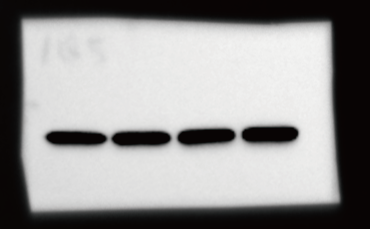

Supplement: Figure 1—source data 2. [file elife-99939-fig1-data2.zip › Figure 1-source data 2/Figure 1G NLRP3.tif]

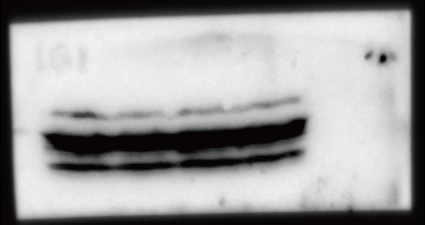

Supplement: Figure 1—source data 2. [file elife-99939-fig1-data2.zip › Figure 1-source data 2/Figure 1G pro-caspase-11.tif]

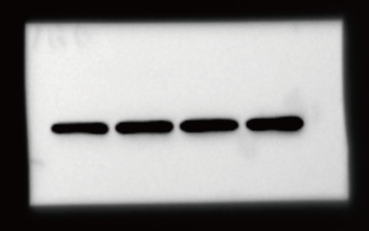

Supplement: Figure 1—source data 2. [file elife-99939-fig1-data2.zip › Figure 1-source data 2/Figure 1G β-actin.tif]

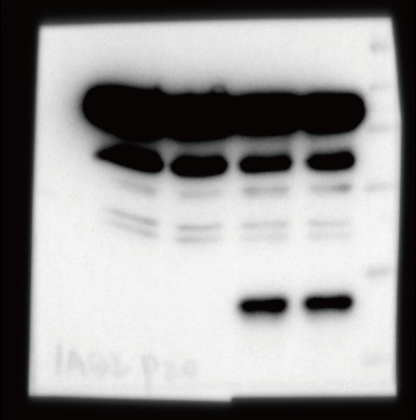

Supplement: Figure 1—source data 2. [file elife-99939-fig1-data2.zip › Figure 1-source data 2/Figure 1K caspase-1.tif]

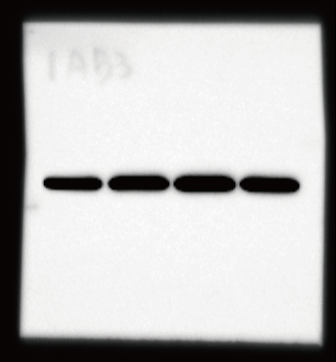

Supplement: Figure 1—source data 2. [file elife-99939-fig1-data2.zip › Figure 1-source data 2/Figure 1K NLRP3.tif]

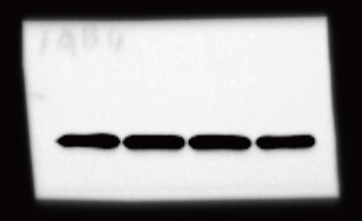

Supplement: Figure 1—source data 2. [file elife-99939-fig1-data2.zip › Figure 1-source data 2/Figure 1K β-actin.tif]

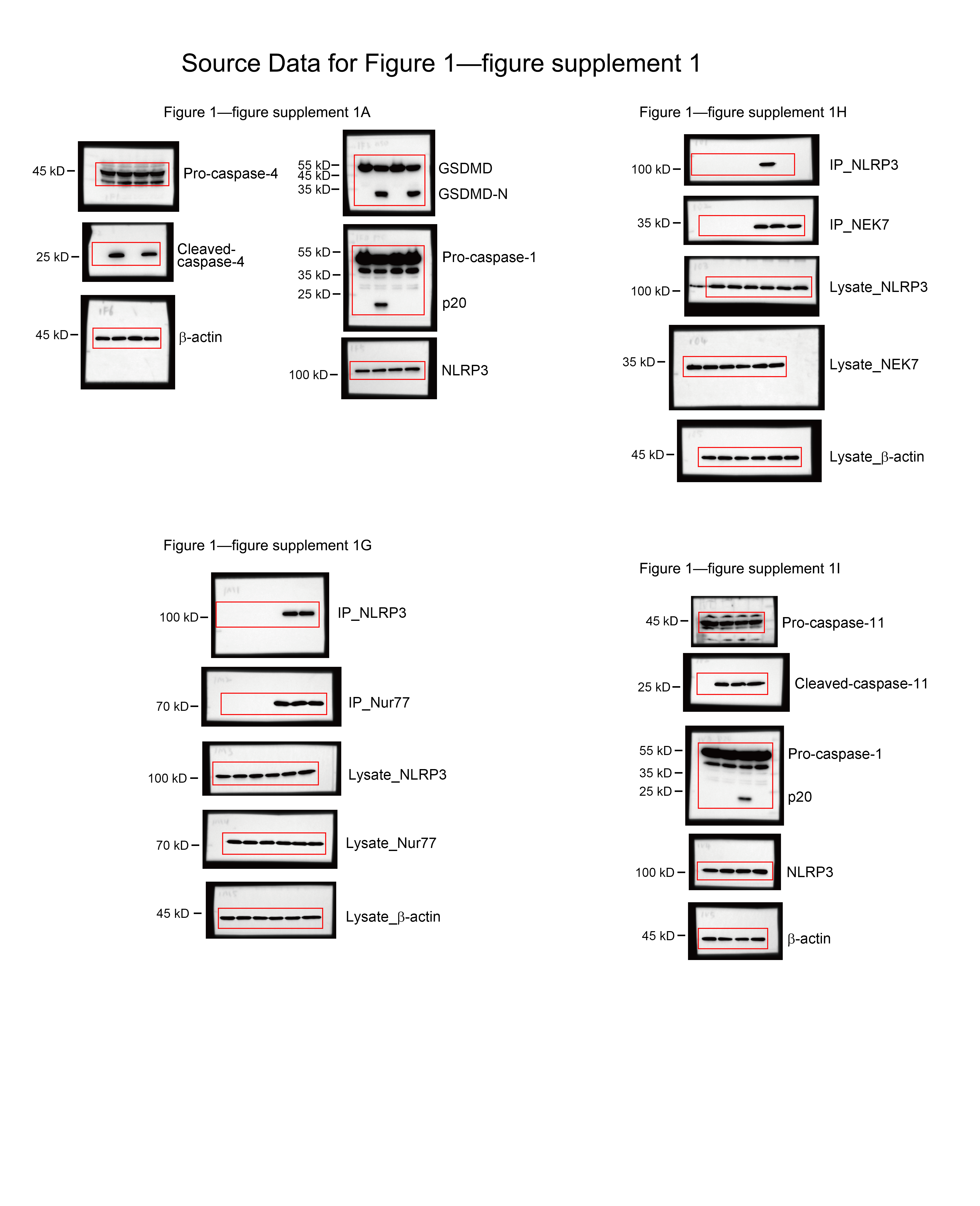

Supplement: Figure 1—figure supplement 1—source data 1. [file elife-99939-fig1-figsupp1-data1.zip › Figure 1-figure supplement 1.tif]

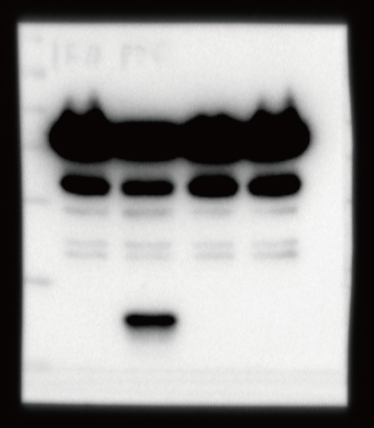

Supplement: Figure 1—figure supplement 1—source data 2. [file elife-99939-fig1-figsupp1-data2.zip › Figure 1-figure supplement 1-source data 2/Figure 1—figure supplement 1A caspase-1.tif]

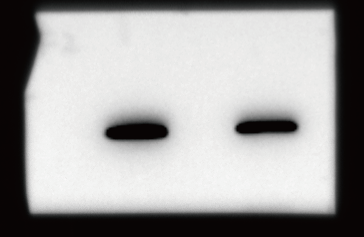

Supplement: Figure 1—figure supplement 1—source data 2. [file elife-99939-fig1-figsupp1-data2.zip › Figure 1-figure supplement 1-source data 2/Figure 1—figure supplement 1A cleaved-caspase-4.tif]

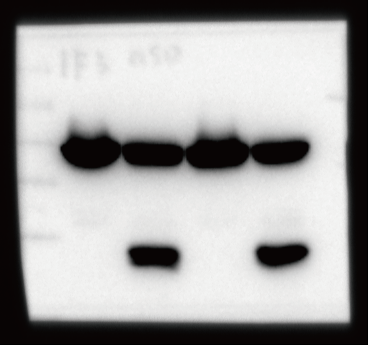

Supplement: Figure 1—figure supplement 1—source data 2. [file elife-99939-fig1-figsupp1-data2.zip › Figure 1-figure supplement 1-source data 2/Figure 1—figure supplement 1A GSDMD.tif]

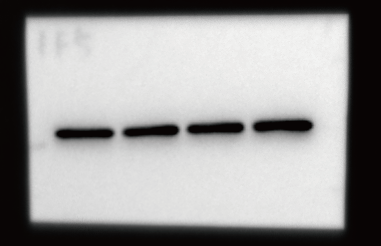

Supplement: Figure 1—figure supplement 1—source data 2. [file elife-99939-fig1-figsupp1-data2.zip › Figure 1-figure supplement 1-source data 2/Figure 1—figure supplement 1A NLRP3.tif]

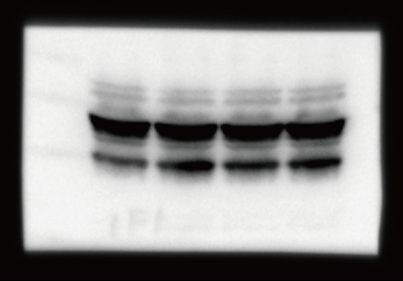

Supplement: Figure 1—figure supplement 1—source data 2. [file elife-99939-fig1-figsupp1-data2.zip › Figure 1-figure supplement 1-source data 2/Figure 1—figure supplement 1A pro-caspase-4.tif]

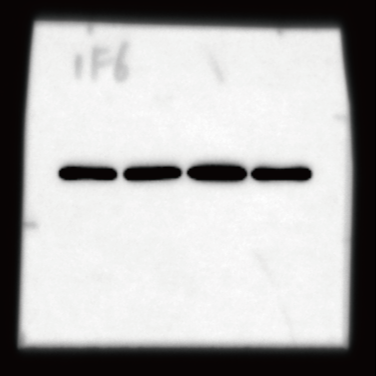

Supplement: Figure 1—figure supplement 1—source data 2. [file elife-99939-fig1-figsupp1-data2.zip › Figure 1-figure supplement 1-source data 2/Figure 1—figure supplement 1A β-actin.tif]

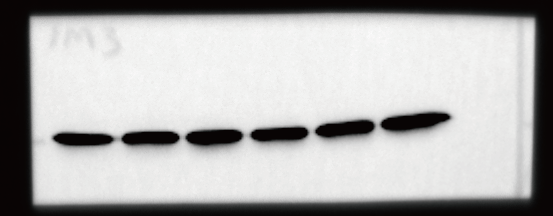

Supplement: Figure 1—figure supplement 1—source data 2. [file elife-99939-fig1-figsupp1-data2.zip › Figure 1-figure supplement 1-source data 2/Figure 1—figure supplement 1G input_NLRP3.tif]

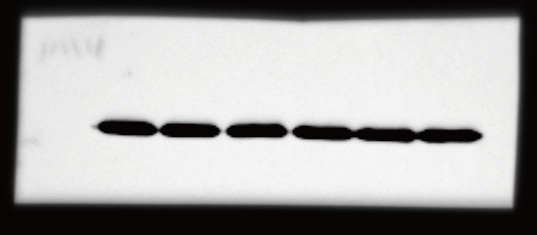

Supplement: Figure 1—figure supplement 1—source data 2. [file elife-99939-fig1-figsupp1-data2.zip › Figure 1-figure supplement 1-source data 2/Figure 1—figure supplement 1G input_Nur77.tif]

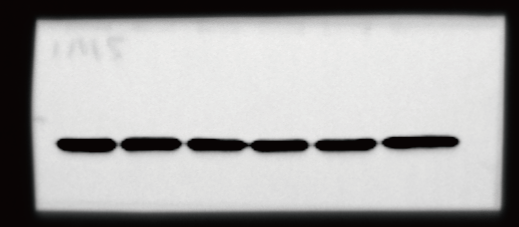

Supplement: Figure 1—figure supplement 1—source data 2. [file elife-99939-fig1-figsupp1-data2.zip › Figure 1-figure supplement 1-source data 2/Figure 1—figure supplement 1G input_β-actin.tif]

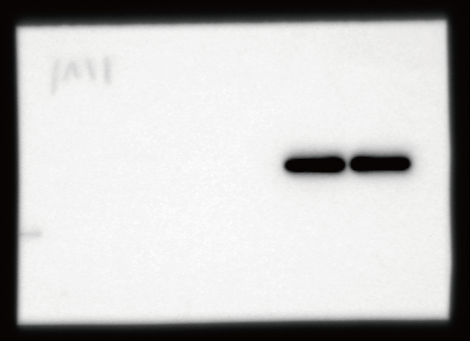

Supplement: Figure 1—figure supplement 1—source data 2. [file elife-99939-fig1-figsupp1-data2.zip › Figure 1-figure supplement 1-source data 2/Figure 1—figure supplement 1G IP_NLRP3.tif]

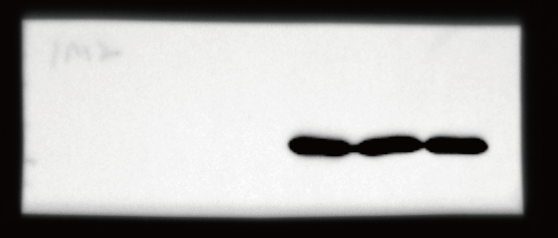

Supplement: Figure 1—figure supplement 1—source data 2. [file elife-99939-fig1-figsupp1-data2.zip › Figure 1-figure supplement 1-source data 2/Figure 1—figure supplement 1G IP_Nur77.tif]

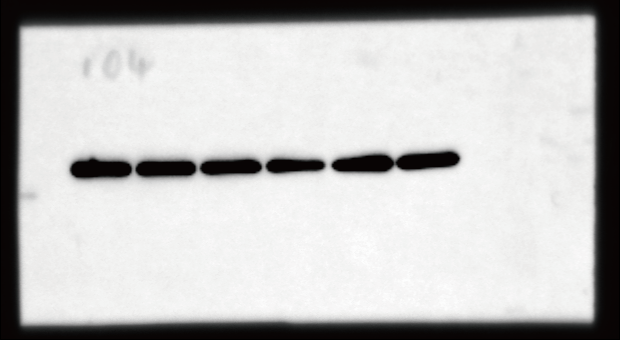

Supplement: Figure 1—figure supplement 1—source data 2. [file elife-99939-fig1-figsupp1-data2.zip › Figure 1-figure supplement 1-source data 2/Figure 1—figure supplement 1H input_NEK7.tif]

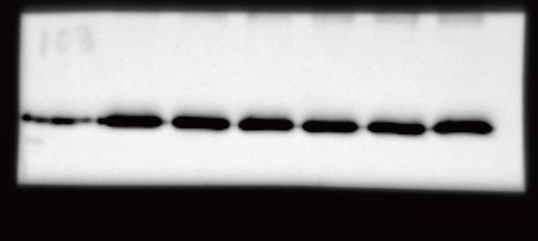

Supplement: Figure 1—figure supplement 1—source data 2. [file elife-99939-fig1-figsupp1-data2.zip › Figure 1-figure supplement 1-source data 2/Figure 1—figure supplement 1H input_NLRP3.tif]

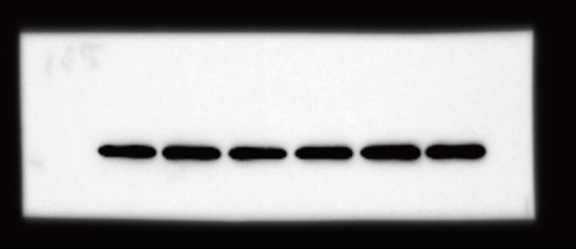

Supplement: Figure 1—figure supplement 1—source data 2. [file elife-99939-fig1-figsupp1-data2.zip › Figure 1-figure supplement 1-source data 2/Figure 1—figure supplement 1H input_β-actin.tif]

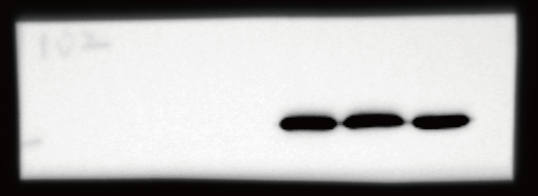

Supplement: Figure 1—figure supplement 1—source data 2. [file elife-99939-fig1-figsupp1-data2.zip › Figure 1-figure supplement 1-source data 2/Figure 1—figure supplement 1H IP_NEK7.tif]

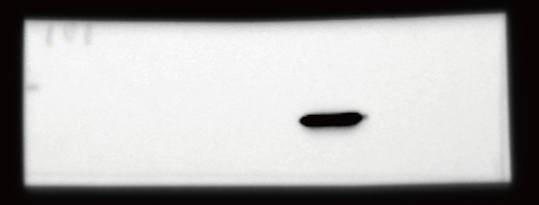

Supplement: Figure 1—figure supplement 1—source data 2. [file elife-99939-fig1-figsupp1-data2.zip › Figure 1-figure supplement 1-source data 2/Figure 1—figure supplement 1H IP_NLRP3.tif]

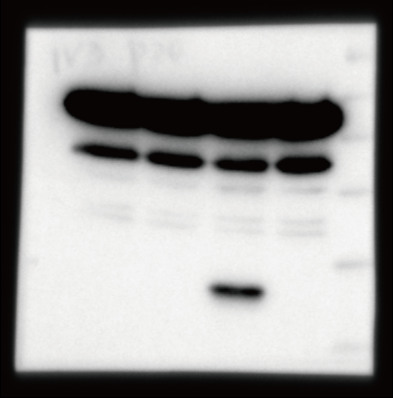

Supplement: Figure 1—figure supplement 1—source data 2. [file elife-99939-fig1-figsupp1-data2.zip › Figure 1-figure supplement 1-source data 2/Figure 1—figure supplement 1I caspase-1.tif]

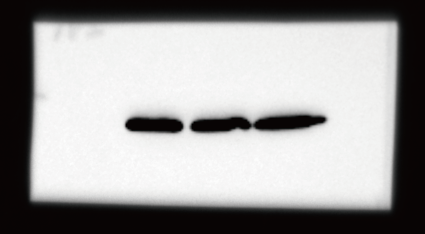

Supplement: Figure 1—figure supplement 1—source data 2. [file elife-99939-fig1-figsupp1-data2.zip › Figure 1-figure supplement 1-source data 2/Figure 1—figure supplement 1I cleaved-caspase-11.tif]

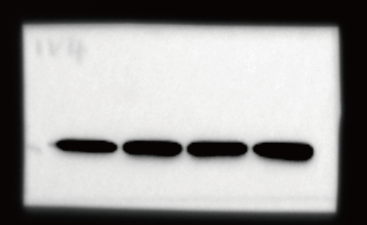

Supplement: Figure 1—figure supplement 1—source data 2. [file elife-99939-fig1-figsupp1-data2.zip › Figure 1-figure supplement 1-source data 2/Figure 1—figure supplement 1I NLRP3.tif]

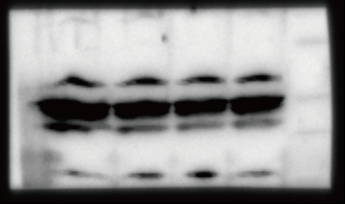

Supplement: Figure 1—figure supplement 1—source data 2. [file elife-99939-fig1-figsupp1-data2.zip › Figure 1-figure supplement 1-source data 2/Figure 1—figure supplement 1I pro-caspase-11.tif]

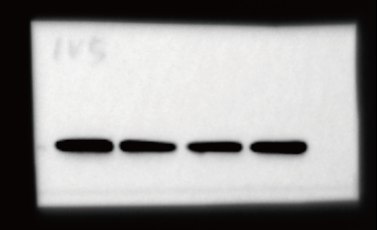

Supplement: Figure 1—figure supplement 1—source data 2. [file elife-99939-fig1-figsupp1-data2.zip › Figure 1-figure supplement 1-source data 2/Figure 1—figure supplement 1I β-actin.tif]

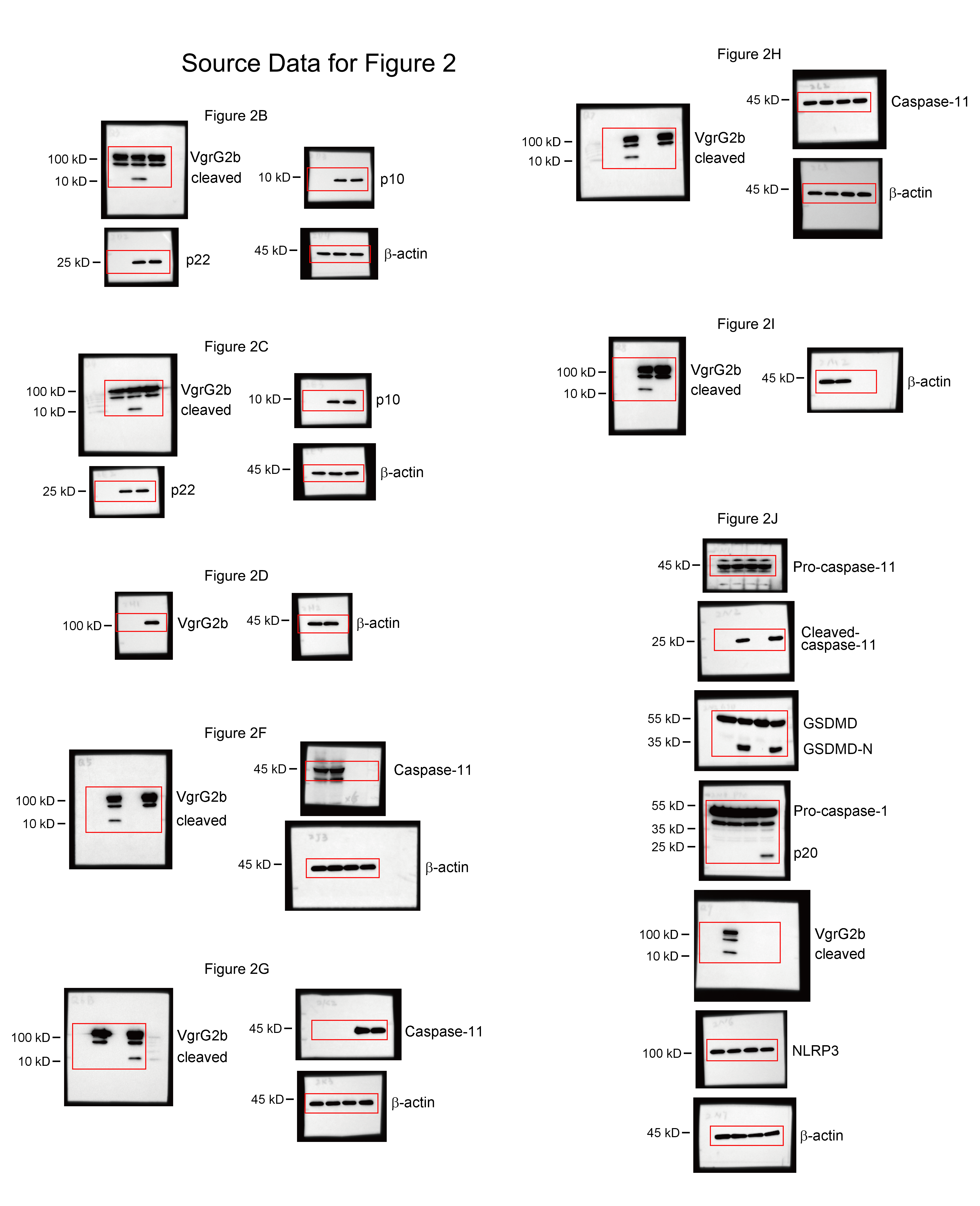

Supplement: Figure 2—source data 1. [file elife-99939-fig2-data1.zip › Figure 2-source data 1.tif]

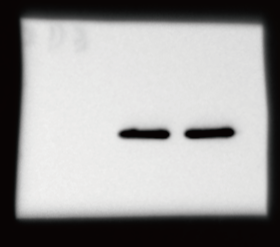

Supplement: Figure 2—source data 2. [file elife-99939-fig2-data2.zip › Figure 2-source data 2/Figure 2B p10.tif]

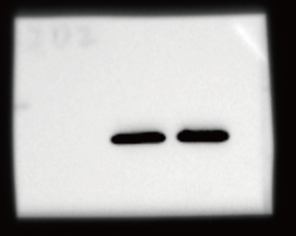

Supplement: Figure 2—source data 2. [file elife-99939-fig2-data2.zip › Figure 2-source data 2/Figure 2B p22.tif]

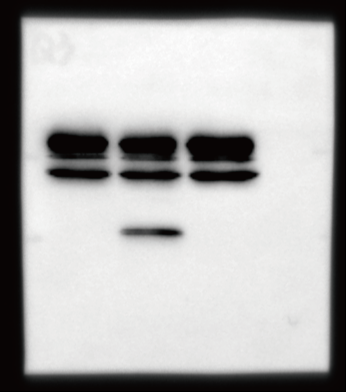

Supplement: Figure 2—source data 2. [file elife-99939-fig2-data2.zip › Figure 2-source data 2/Figure 2B VgrG2b.tif]

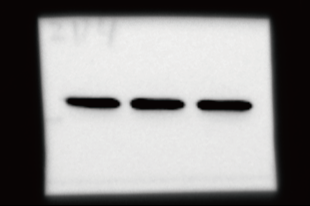

Supplement: Figure 2—source data 2. [file elife-99939-fig2-data2.zip › Figure 2-source data 2/Figure 2B β-actin.tif]

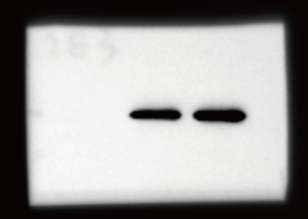

Supplement: Figure 2—source data 2. [file elife-99939-fig2-data2.zip › Figure 2-source data 2/Figure 2C p10.tif]

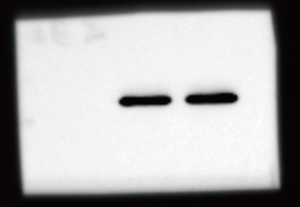

Supplement: Figure 2—source data 2. [file elife-99939-fig2-data2.zip › Figure 2-source data 2/Figure 2C p22.tif]

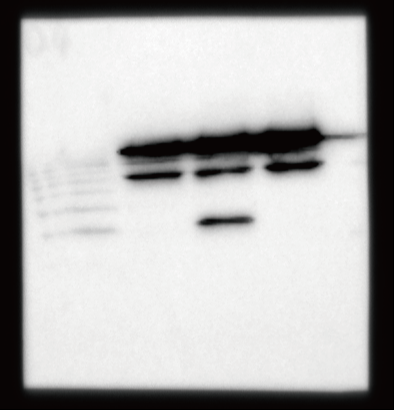

Supplement: Figure 2—source data 2. [file elife-99939-fig2-data2.zip › Figure 2-source data 2/Figure 2C VgrG2b.tif]

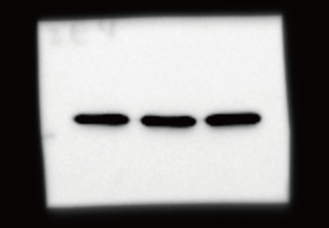

Supplement: Figure 2—source data 2. [file elife-99939-fig2-data2.zip › Figure 2-source data 2/Figure 2C β-actin.tif]

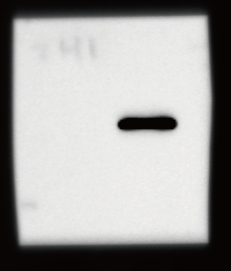

Supplement: Figure 2—source data 2. [file elife-99939-fig2-data2.zip › Figure 2-source data 2/Figure 2D VgrG2b.tif]

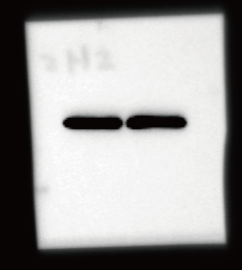

Supplement: Figure 2—source data 2. [file elife-99939-fig2-data2.zip › Figure 2-source data 2/Figure 2D β-actin.tif]

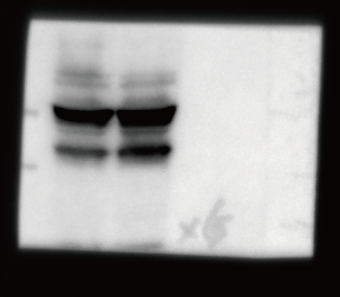

Supplement: Figure 2—source data 2. [file elife-99939-fig2-data2.zip › Figure 2-source data 2/Figure 2F caspase-11.tif]

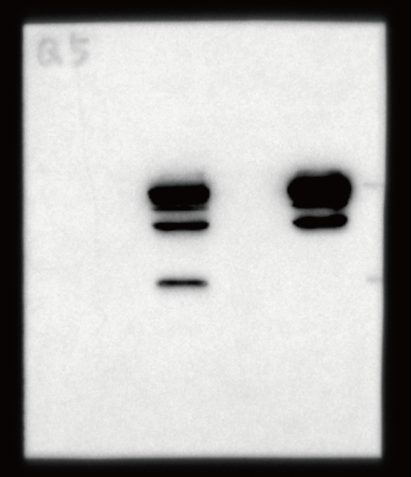

Supplement: Figure 2—source data 2. [file elife-99939-fig2-data2.zip › Figure 2-source data 2/Figure 2F VgrG2b.tif]

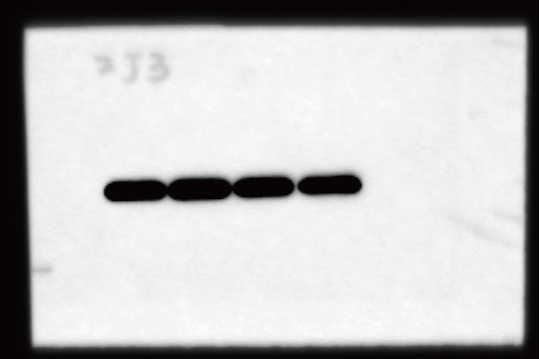

Supplement: Figure 2—source data 2. [file elife-99939-fig2-data2.zip › Figure 2-source data 2/Figure 2F β-actin.tif]

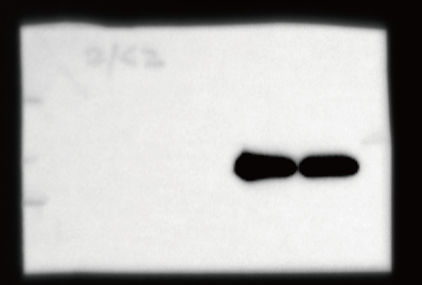

Supplement: Figure 2—source data 2. [file elife-99939-fig2-data2.zip › Figure 2-source data 2/Figure 2G caspase-11.tif]

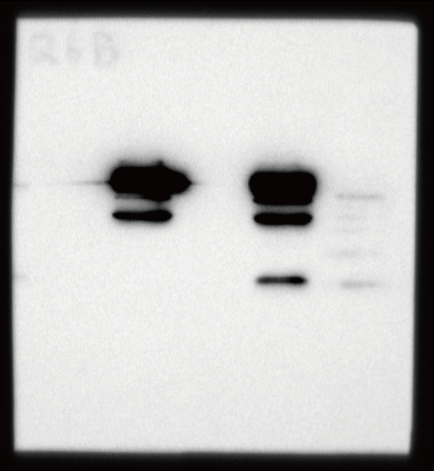

Supplement: Figure 2—source data 2. [file elife-99939-fig2-data2.zip › Figure 2-source data 2/Figure 2G VgrG2b.tif]

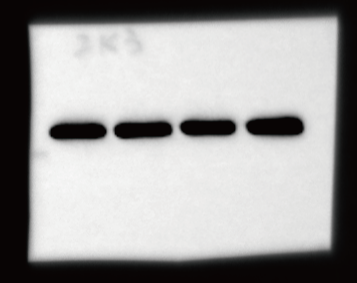

Supplement: Figure 2—source data 2. [file elife-99939-fig2-data2.zip › Figure 2-source data 2/Figure 2G β-actin.tif]

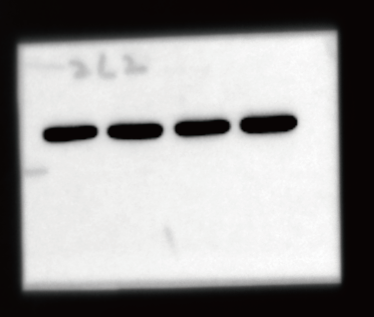

Supplement: Figure 2—source data 2. [file elife-99939-fig2-data2.zip › Figure 2-source data 2/Figure 2H caspase-11.tif]

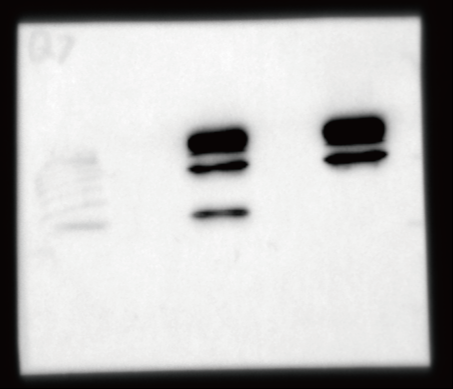

Supplement: Figure 2—source data 2. [file elife-99939-fig2-data2.zip › Figure 2-source data 2/Figure 2H VgrG2b.tif]

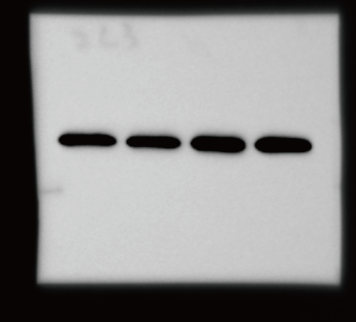

Supplement: Figure 2—source data 2. [file elife-99939-fig2-data2.zip › Figure 2-source data 2/Figure 2H β-actin.tif]

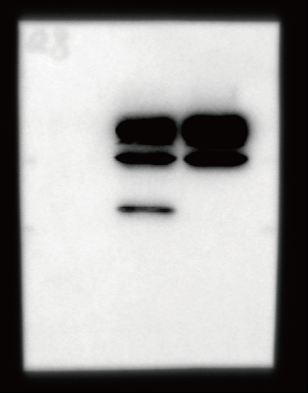

Supplement: Figure 2—source data 2. [file elife-99939-fig2-data2.zip › Figure 2-source data 2/Figure 2I VgrG2b.tif]

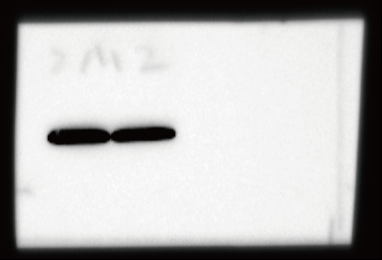

Supplement: Figure 2—source data 2. [file elife-99939-fig2-data2.zip › Figure 2-source data 2/Figure 2I β-actin.tif]

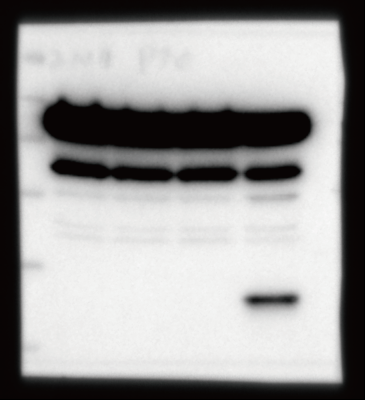

Supplement: Figure 2—source data 2. [file elife-99939-fig2-data2.zip › Figure 2-source data 2/Figure 2J caspase-1.tif]

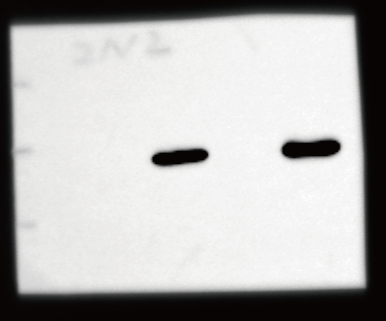

Supplement: Figure 2—source data 2. [file elife-99939-fig2-data2.zip › Figure 2-source data 2/Figure 2J cleaved-caspase-11.tif]

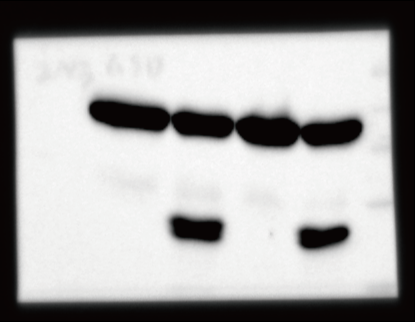

Supplement: Figure 2—source data 2. [file elife-99939-fig2-data2.zip › Figure 2-source data 2/Figure 2J GSDMD.tif]

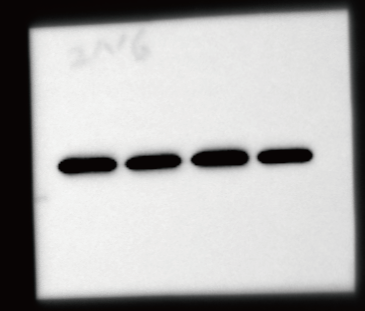

Supplement: Figure 2—source data 2. [file elife-99939-fig2-data2.zip › Figure 2-source data 2/Figure 2J NLRP3.tif]

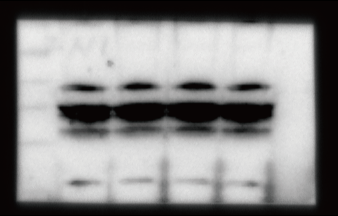

Supplement: Figure 2—source data 2. [file elife-99939-fig2-data2.zip › Figure 2-source data 2/Figure 2J pro-caspase-11.tif]

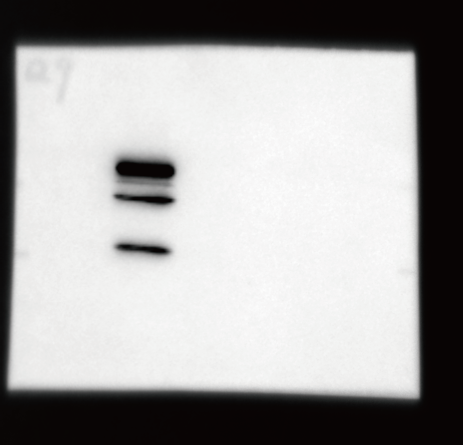

Supplement: Figure 2—source data 2. [file elife-99939-fig2-data2.zip › Figure 2-source data 2/Figure 2J VgrG2b.tif]

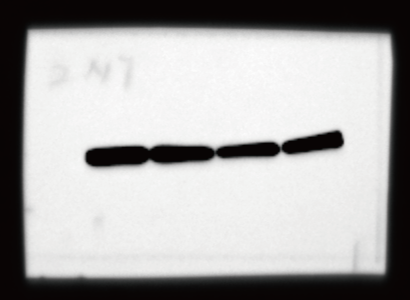

Supplement: Figure 2—source data 2. [file elife-99939-fig2-data2.zip › Figure 2-source data 2/Figure 2J β-actin.tif]

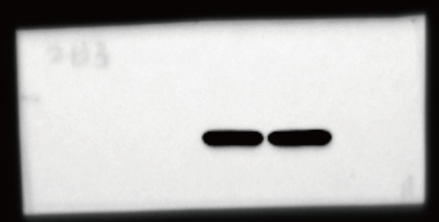

Supplement: Figure 2—figure supplement 1—source data 2. [file elife-99939-fig2-figsupp1-data2.zip › Figure 2-figure supplement 1-source data 2/Figure 2—figure supplement 1A p10.tif]

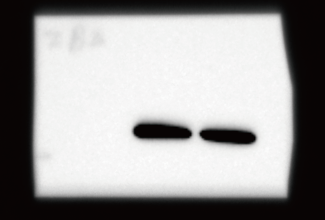

Supplement: Figure 2—figure supplement 1—source data 2. [file elife-99939-fig2-figsupp1-data2.zip › Figure 2-figure supplement 1-source data 2/Figure 2—figure supplement 1A p22.tif]

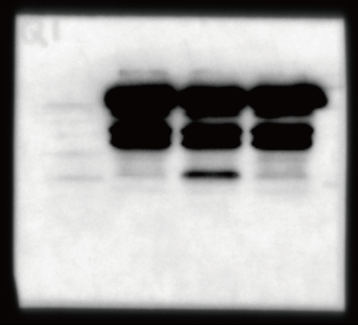

Supplement: Figure 2—figure supplement 1—source data 2. [file elife-99939-fig2-figsupp1-data2.zip › Figure 2-figure supplement 1-source data 2/Figure 2—figure supplement 1A VgrG1a.tif]

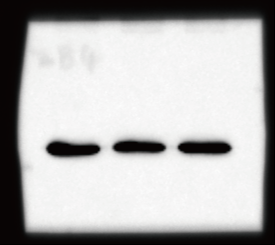

Supplement: Figure 2—figure supplement 1—source data 2. [file elife-99939-fig2-figsupp1-data2.zip › Figure 2-figure supplement 1-source data 2/Figure 2—figure supplement 1A β-actin.tif]

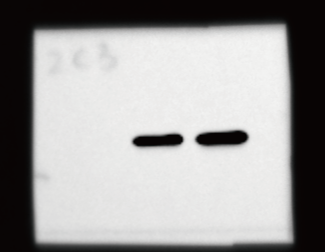

Supplement: Figure 2—figure supplement 1—source data 2. [file elife-99939-fig2-figsupp1-data2.zip › Figure 2-figure supplement 1-source data 2/Figure 2—figure supplement 1B p10.tif]

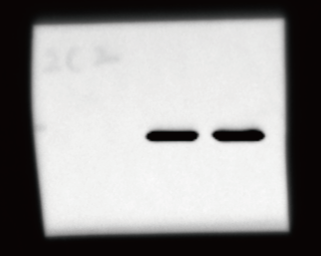

Supplement: Figure 2—figure supplement 1—source data 2. [file elife-99939-fig2-figsupp1-data2.zip › Figure 2-figure supplement 1-source data 2/Figure 2—figure supplement 1B p22.tif]

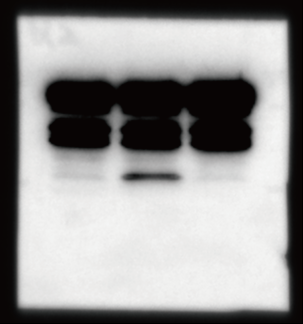

Supplement: Figure 2—figure supplement 1—source data 2. [file elife-99939-fig2-figsupp1-data2.zip › Figure 2-figure supplement 1-source data 2/Figure 2—figure supplement 1B VgrG1a.tif]

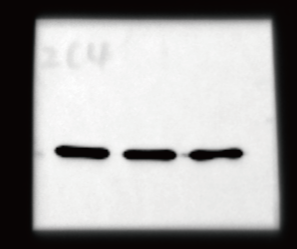

Supplement: Figure 2—figure supplement 1—source data 2. [file elife-99939-fig2-figsupp1-data2.zip › Figure 2-figure supplement 1-source data 2/Figure 2—figure supplement 1B β-actin.tif]

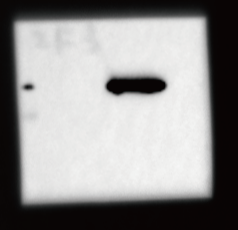

Supplement: Figure 2—figure supplement 1—source data 2. [file elife-99939-fig2-figsupp1-data2.zip › Figure 2-figure supplement 1-source data 2/Figure 2—figure supplement 1C p10.tif]

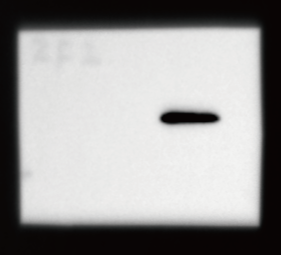

Supplement: Figure 2—figure supplement 1—source data 2. [file elife-99939-fig2-figsupp1-data2.zip › Figure 2-figure supplement 1-source data 2/Figure 2—figure supplement 1C p22.tif]

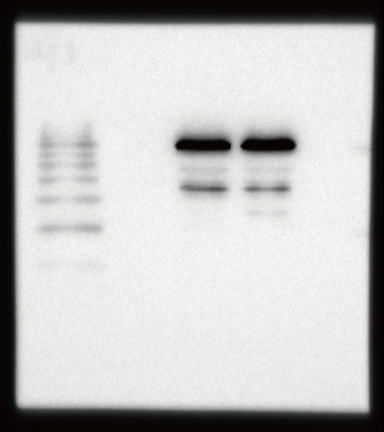

Supplement: Figure 2—figure supplement 1—source data 2. [file elife-99939-fig2-figsupp1-data2.zip › Figure 2-figure supplement 1-source data 2/Figure 2—figure supplement 1C VgrG2a.tif]

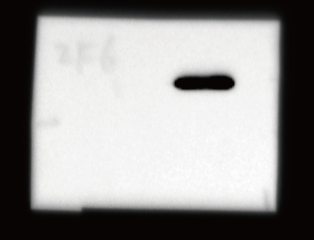

Supplement: Figure 2—figure supplement 1—source data 2. [file elife-99939-fig2-figsupp1-data2.zip › Figure 2-figure supplement 1-source data 2/Figure 2—figure supplement 1D p10.tif]

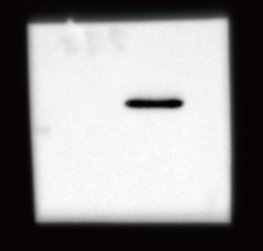

Supplement: Figure 2—figure supplement 1—source data 2. [file elife-99939-fig2-figsupp1-data2.zip › Figure 2-figure supplement 1-source data 2/Figure 2—figure supplement 1D p22.tif]

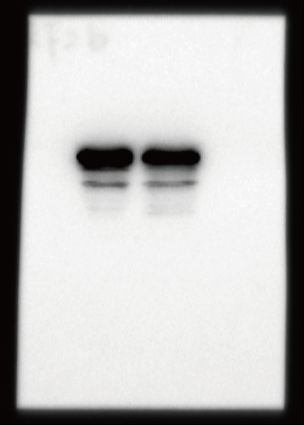

Supplement: Figure 2—figure supplement 1—source data 2. [file elife-99939-fig2-figsupp1-data2.zip › Figure 2-figure supplement 1-source data 2/Figure 2—figure supplement 1D VgrG3.tif]

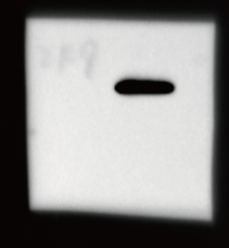

Supplement: Figure 2—figure supplement 1—source data 2. [file elife-99939-fig2-figsupp1-data2.zip › Figure 2-figure supplement 1-source data 2/Figure 2—figure supplement 1E p10.tif]

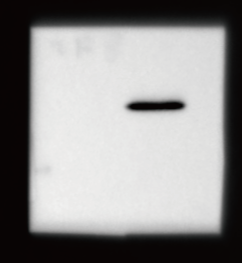

Supplement: Figure 2—figure supplement 1—source data 2. [file elife-99939-fig2-figsupp1-data2.zip › Figure 2-figure supplement 1-source data 2/Figure 2—figure supplement 1E p22.tif]

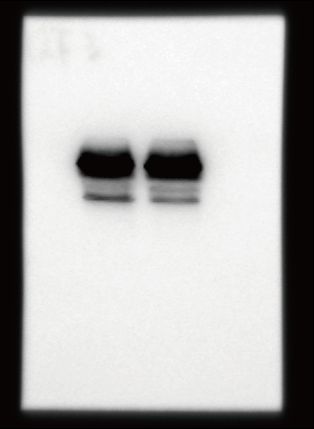

Supplement: Figure 2—figure supplement 1—source data 2. [file elife-99939-fig2-figsupp1-data2.zip › Figure 2-figure supplement 1-source data 2/Figure 2—figure supplement 1E VgrG4a.tif]

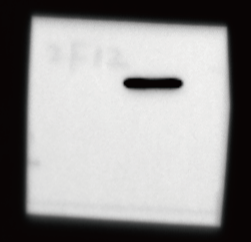

Supplement: Figure 2—figure supplement 1—source data 2. [file elife-99939-fig2-figsupp1-data2.zip › Figure 2-figure supplement 1-source data 2/Figure 2—figure supplement 1F p10.tif]

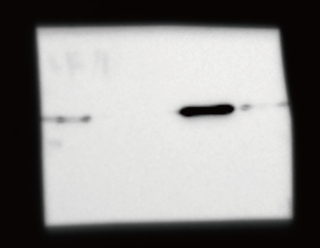

Supplement: Figure 2—figure supplement 1—source data 2. [file elife-99939-fig2-figsupp1-data2.zip › Figure 2-figure supplement 1-source data 2/Figure 2—figure supplement 1F p22.tif]

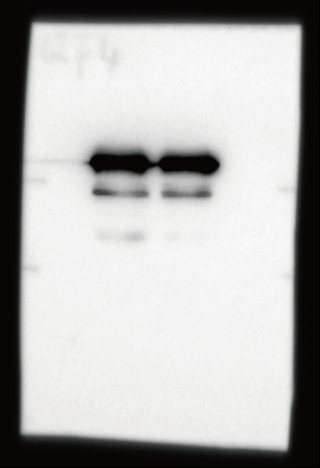

Supplement: Figure 2—figure supplement 1—source data 2. [file elife-99939-fig2-figsupp1-data2.zip › Figure 2-figure supplement 1-source data 2/Figure 2—figure supplement 1F VgrG5.tif]

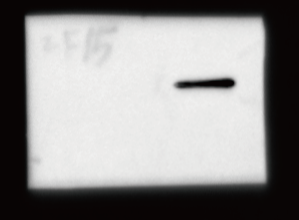

Supplement: Figure 2—figure supplement 1—source data 2. [file elife-99939-fig2-figsupp1-data2.zip › Figure 2-figure supplement 1-source data 2/Figure 2—figure supplement 1G p10.tif]

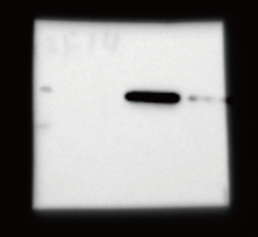

Supplement: Figure 2—figure supplement 1—source data 2. [file elife-99939-fig2-figsupp1-data2.zip › Figure 2-figure supplement 1-source data 2/Figure 2—figure supplement 1G p22.tif]

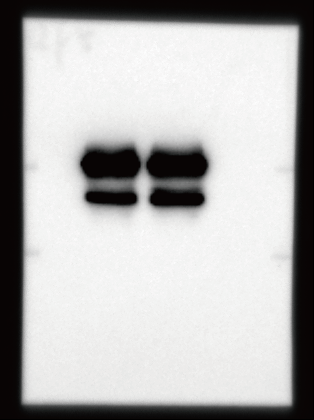

Supplement: Figure 2—figure supplement 1—source data 2. [file elife-99939-fig2-figsupp1-data2.zip › Figure 2-figure supplement 1-source data 2/Figure 2—figure supplement 1G VgrG6.tif]

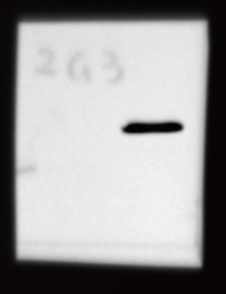

Supplement: Figure 2—figure supplement 1—source data 2. [file elife-99939-fig2-figsupp1-data2.zip › Figure 2-figure supplement 1-source data 2/Figure 2—figure supplement 1H p10.tif]

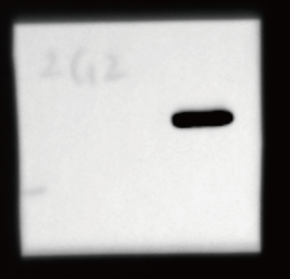

Supplement: Figure 2—figure supplement 1—source data 2. [file elife-99939-fig2-figsupp1-data2.zip › Figure 2-figure supplement 1-source data 2/Figure 2—figure supplement 1H p22.tif]

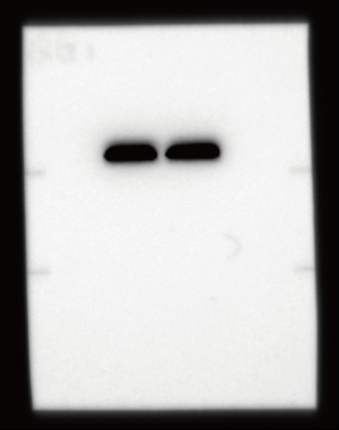

Supplement: Figure 2—figure supplement 1—source data 2. [file elife-99939-fig2-figsupp1-data2.zip › Figure 2-figure supplement 1-source data 2/Figure 2—figure supplement 1H VgrG2a.tif]

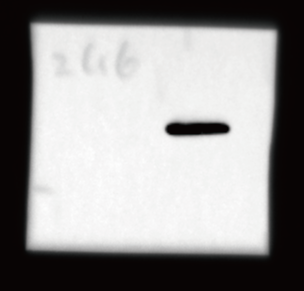

Supplement: Figure 2—figure supplement 1—source data 2. [file elife-99939-fig2-figsupp1-data2.zip › Figure 2-figure supplement 1-source data 2/Figure 2—figure supplement 1I p10.tif]

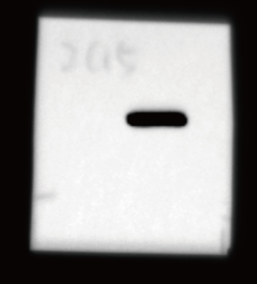

Supplement: Figure 2—figure supplement 1—source data 2. [file elife-99939-fig2-figsupp1-data2.zip › Figure 2-figure supplement 1-source data 2/Figure 2—figure supplement 1I p22.tif]

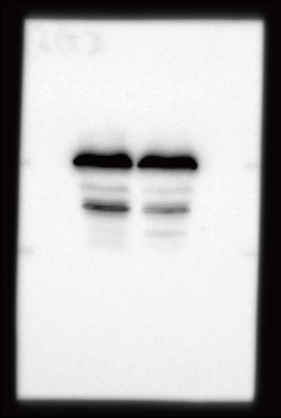

Supplement: Figure 2—figure supplement 1—source data 2. [file elife-99939-fig2-figsupp1-data2.zip › Figure 2-figure supplement 1-source data 2/Figure 2—figure supplement 1I VgrG3.tif]

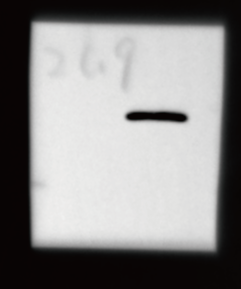

Supplement: Figure 2—figure supplement 1—source data 2. [file elife-99939-fig2-figsupp1-data2.zip › Figure 2-figure supplement 1-source data 2/Figure 2—figure supplement 1J p10.tif]
