# Supplementary figures and images for: A VgrG2b fragment cleaved by caspase-11/4 promotes Pseudomonas aeruginosa infection through suppressing the NLRP3 inflammasome (part 2 of 4)
Source: eLife. 2025 Feb 25;13:RP99939. doi: 10.7554/eLife.99939 (PMC11856931; doi:10.7554/eLife.99939)

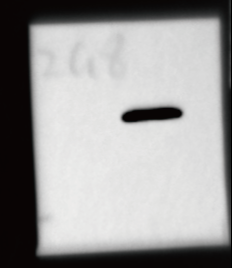

Supplement: Figure 2—figure supplement 1—source data 2. [file elife-99939-fig2-figsupp1-data2.zip › Figure 2-figure supplement 1-source data 2/Figure 2—figure supplement 1J p22.tif]

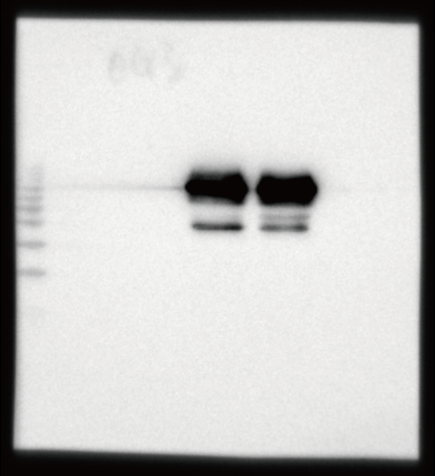

Supplement: Figure 2—figure supplement 1—source data 2. [file elife-99939-fig2-figsupp1-data2.zip › Figure 2-figure supplement 1-source data 2/Figure 2—figure supplement 1J VgrG4a.tif]

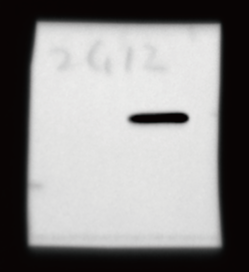

Supplement: Figure 2—figure supplement 1—source data 2. [file elife-99939-fig2-figsupp1-data2.zip › Figure 2-figure supplement 1-source data 2/Figure 2—figure supplement 1K p10.tif]

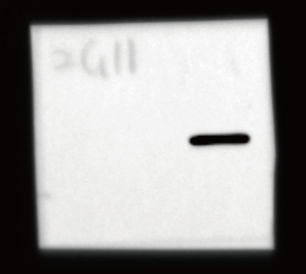

Supplement: Figure 2—figure supplement 1—source data 2. [file elife-99939-fig2-figsupp1-data2.zip › Figure 2-figure supplement 1-source data 2/Figure 2—figure supplement 1K p22.tif]

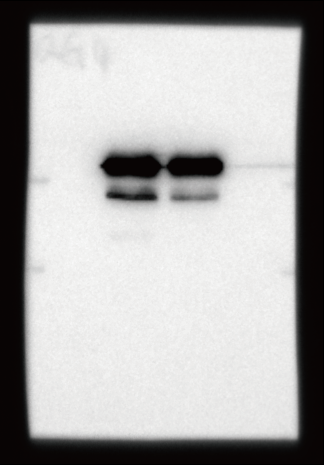

Supplement: Figure 2—figure supplement 1—source data 2. [file elife-99939-fig2-figsupp1-data2.zip › Figure 2-figure supplement 1-source data 2/Figure 2—figure supplement 1K VgrG5.tif]

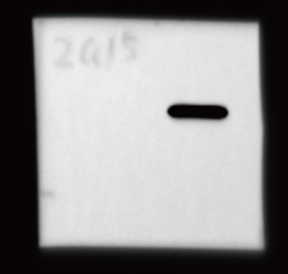

Supplement: Figure 2—figure supplement 1—source data 2. [file elife-99939-fig2-figsupp1-data2.zip › Figure 2-figure supplement 1-source data 2/Figure 2—figure supplement 1L p10.tif]

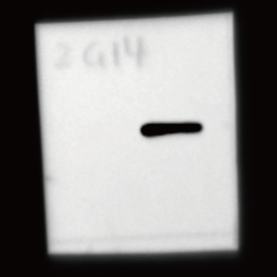

Supplement: Figure 2—figure supplement 1—source data 2. [file elife-99939-fig2-figsupp1-data2.zip › Figure 2-figure supplement 1-source data 2/Figure 2—figure supplement 1L p22.tif]

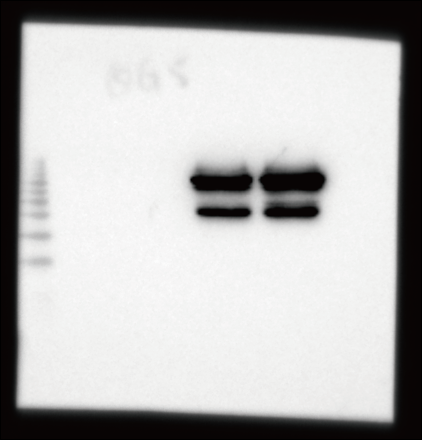

Supplement: Figure 2—figure supplement 1—source data 2. [file elife-99939-fig2-figsupp1-data2.zip › Figure 2-figure supplement 1-source data 2/Figure 2—figure supplement 1L VgrG6.tif]

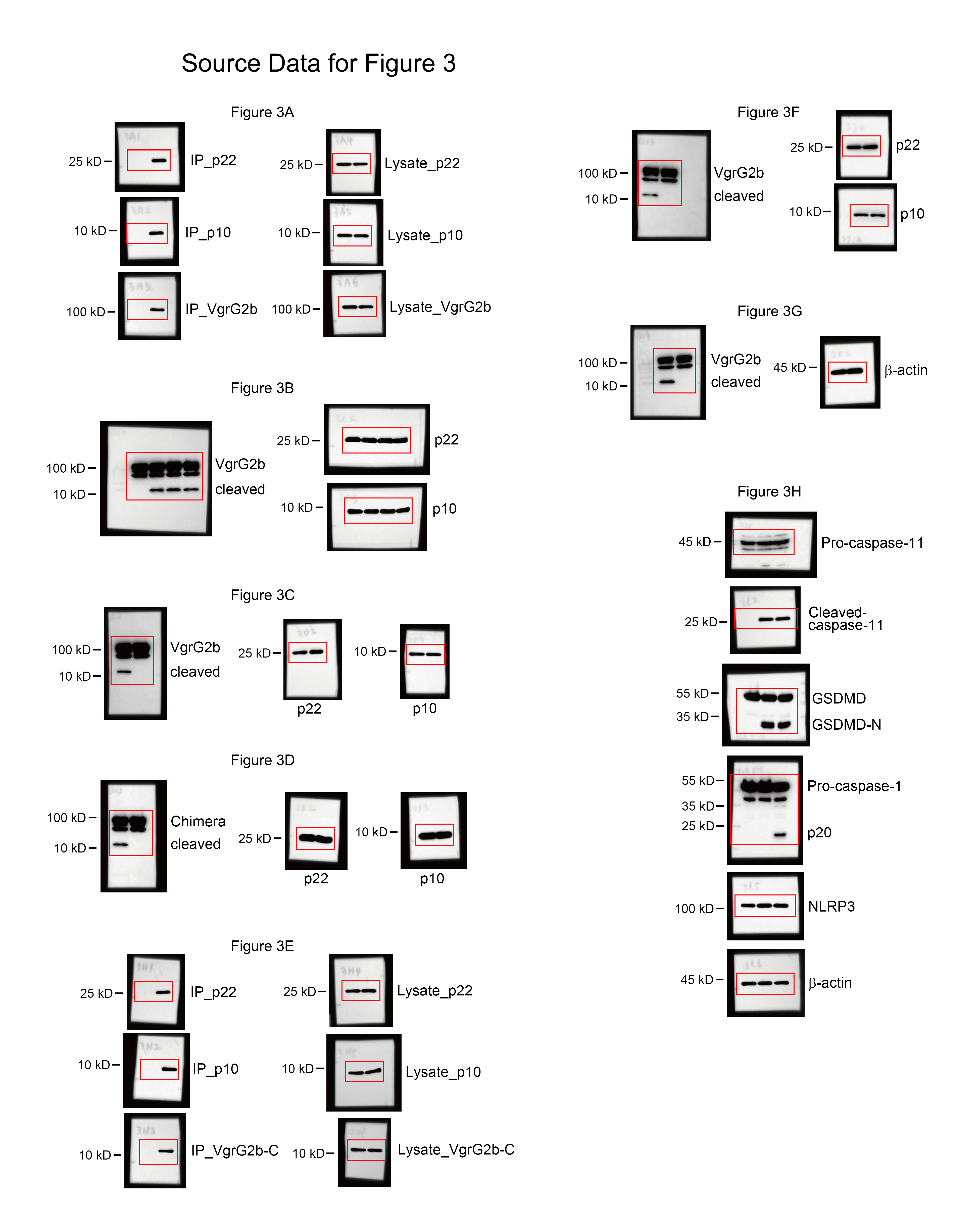

Supplement: Figure 3—source data 1. [file elife-99939-fig3-data1.zip › Figure 3-source data 1.tif]

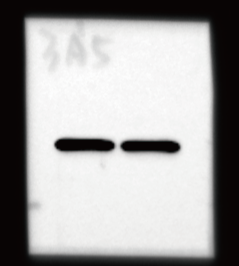

Supplement: Figure 3—source data 2. [file elife-99939-fig3-data2.zip › Figure 3-source data 2/Figure 3A input_p10.tif]

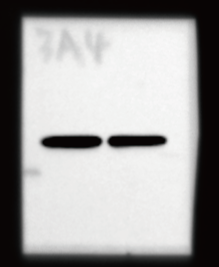

Supplement: Figure 3—source data 2. [file elife-99939-fig3-data2.zip › Figure 3-source data 2/Figure 3A input_p22.tif]

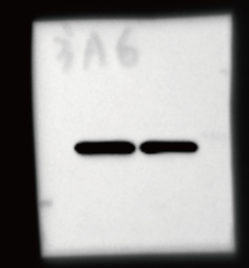

Supplement: Figure 3—source data 2. [file elife-99939-fig3-data2.zip › Figure 3-source data 2/Figure 3A input_VgrG2b.tif]

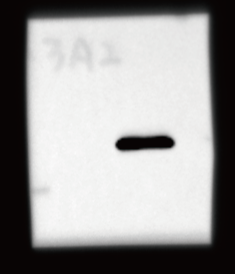

Supplement: Figure 3—source data 2. [file elife-99939-fig3-data2.zip › Figure 3-source data 2/Figure 3A IP_p10.tif]

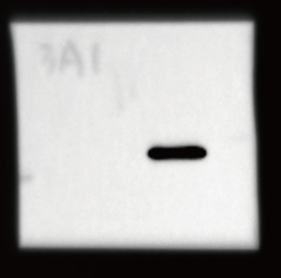

Supplement: Figure 3—source data 2. [file elife-99939-fig3-data2.zip › Figure 3-source data 2/Figure 3A IP_p22.tif]

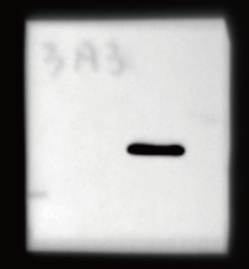

Supplement: Figure 3—source data 2. [file elife-99939-fig3-data2.zip › Figure 3-source data 2/Figure 3A IP_VgrG2b.tif]

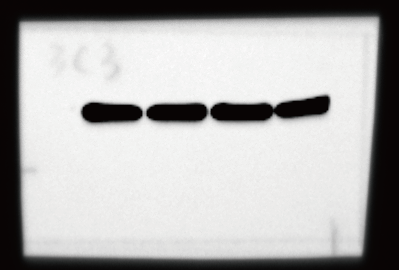

Supplement: Figure 3—source data 2. [file elife-99939-fig3-data2.zip › Figure 3-source data 2/Figure 3B p10.tif]

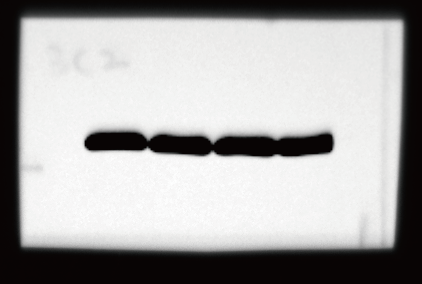

Supplement: Figure 3—source data 2. [file elife-99939-fig3-data2.zip › Figure 3-source data 2/Figure 3B p22.tif]

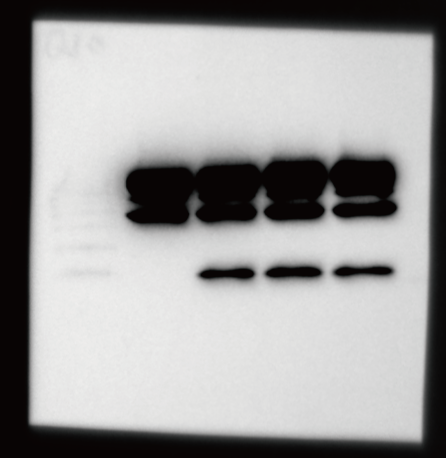

Supplement: Figure 3—source data 2. [file elife-99939-fig3-data2.zip › Figure 3-source data 2/Figure 3B VgrG2b.tif]

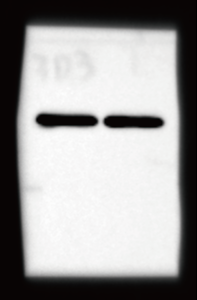

Supplement: Figure 3—source data 2. [file elife-99939-fig3-data2.zip › Figure 3-source data 2/Figure 3C p10.tif]

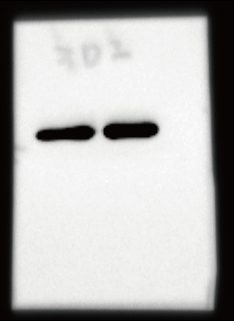

Supplement: Figure 3—source data 2. [file elife-99939-fig3-data2.zip › Figure 3-source data 2/Figure 3C p22.tif]

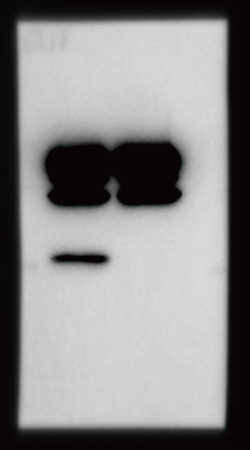

Supplement: Figure 3—source data 2. [file elife-99939-fig3-data2.zip › Figure 3-source data 2/Figure 3C VgrG2b.tif]

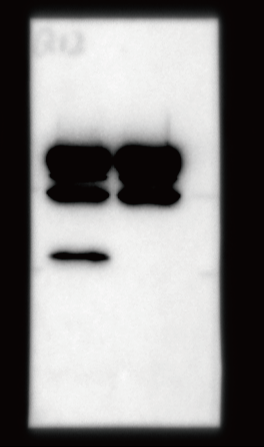

Supplement: Figure 3—source data 2. [file elife-99939-fig3-data2.zip › Figure 3-source data 2/Figure 3D chimera.tif]

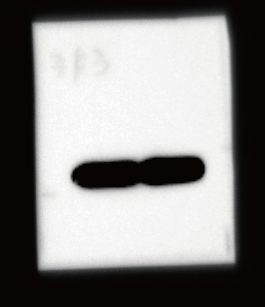

Supplement: Figure 3—source data 2. [file elife-99939-fig3-data2.zip › Figure 3-source data 2/Figure 3D p10.tif]

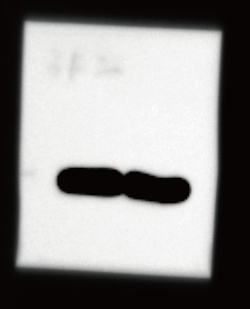

Supplement: Figure 3—source data 2. [file elife-99939-fig3-data2.zip › Figure 3-source data 2/Figure 3D p22.tif]

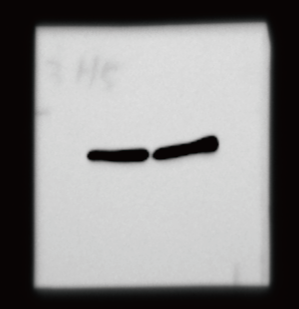

Supplement: Figure 3—source data 2. [file elife-99939-fig3-data2.zip › Figure 3-source data 2/Figure 3E input_p10.tif]

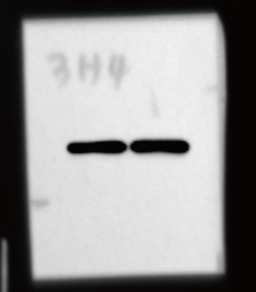

Supplement: Figure 3—source data 2. [file elife-99939-fig3-data2.zip › Figure 3-source data 2/Figure 3E input_p22.tif]

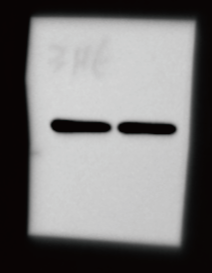

Supplement: Figure 3—source data 2. [file elife-99939-fig3-data2.zip › Figure 3-source data 2/Figure 3E input_VgrG2b-C.tif]

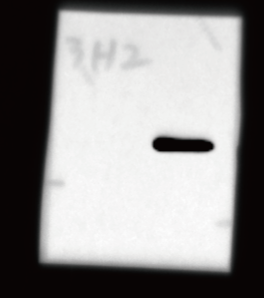

Supplement: Figure 3—source data 2. [file elife-99939-fig3-data2.zip › Figure 3-source data 2/Figure 3E IP_p10.tif]

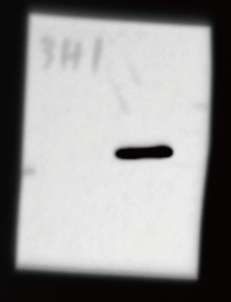

Supplement: Figure 3—source data 2. [file elife-99939-fig3-data2.zip › Figure 3-source data 2/Figure 3E IP_p22.tif]

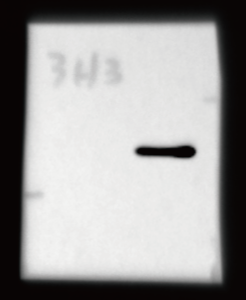

Supplement: Figure 3—source data 2. [file elife-99939-fig3-data2.zip › Figure 3-source data 2/Figure 3E IP_VgrG2b-C.tif]

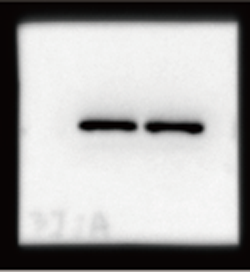

Supplement: Figure 3—source data 2. [file elife-99939-fig3-data2.zip › Figure 3-source data 2/Figure 3F p10.tif]

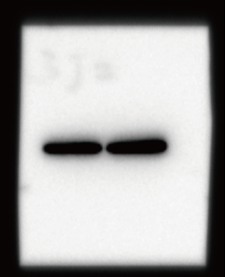

Supplement: Figure 3—source data 2. [file elife-99939-fig3-data2.zip › Figure 3-source data 2/Figure 3F p22.tif]

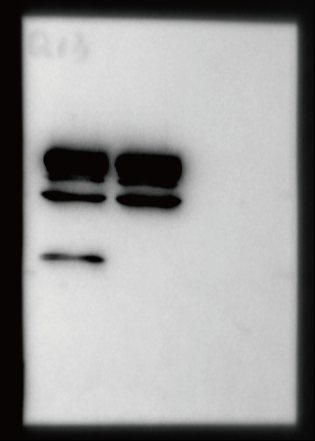

Supplement: Figure 3—source data 2. [file elife-99939-fig3-data2.zip › Figure 3-source data 2/Figure 3F VgrG2b.tif]

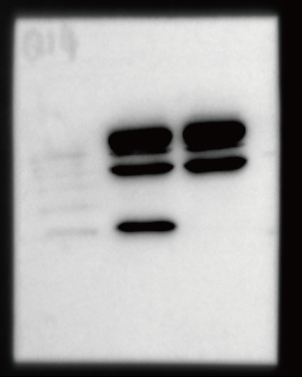

Supplement: Figure 3—source data 2. [file elife-99939-fig3-data2.zip › Figure 3-source data 2/Figure 3G VgrG2b.tif]

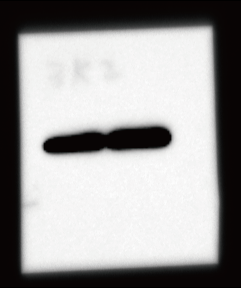

Supplement: Figure 3—source data 2. [file elife-99939-fig3-data2.zip › Figure 3-source data 2/Figure 3G β-actin.tif]

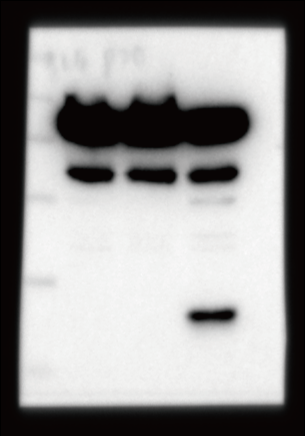

Supplement: Figure 3—source data 2. [file elife-99939-fig3-data2.zip › Figure 3-source data 2/Figure 3H caspase-1.tif]

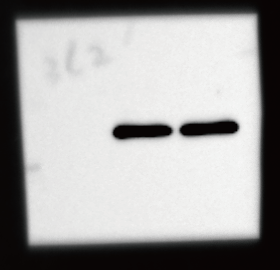

Supplement: Figure 3—source data 2. [file elife-99939-fig3-data2.zip › Figure 3-source data 2/Figure 3H cleaved-caspase-11.tif]

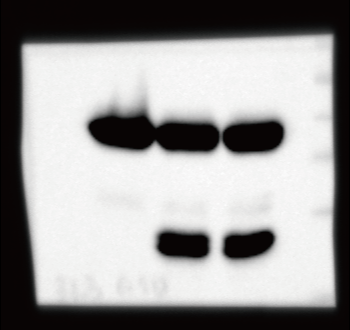

Supplement: Figure 3—source data 2. [file elife-99939-fig3-data2.zip › Figure 3-source data 2/Figure 3H GSDMD.tif]

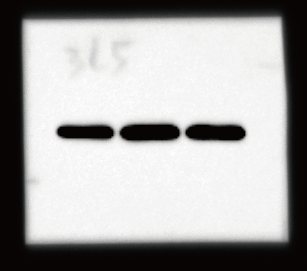

Supplement: Figure 3—source data 2. [file elife-99939-fig3-data2.zip › Figure 3-source data 2/Figure 3H NLRP3.tif]

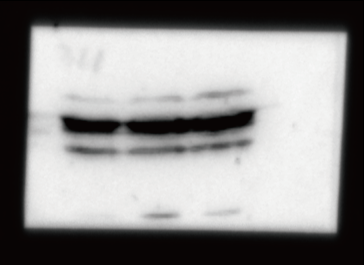

Supplement: Figure 3—source data 2. [file elife-99939-fig3-data2.zip › Figure 3-source data 2/Figure 3H pro-caspase-11.tif]

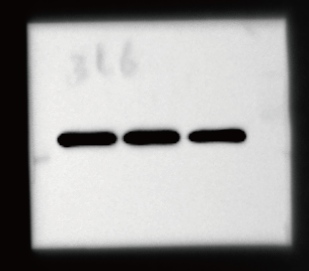

Supplement: Figure 3—source data 2. [file elife-99939-fig3-data2.zip › Figure 3-source data 2/Figure 3H β-actin.tif]

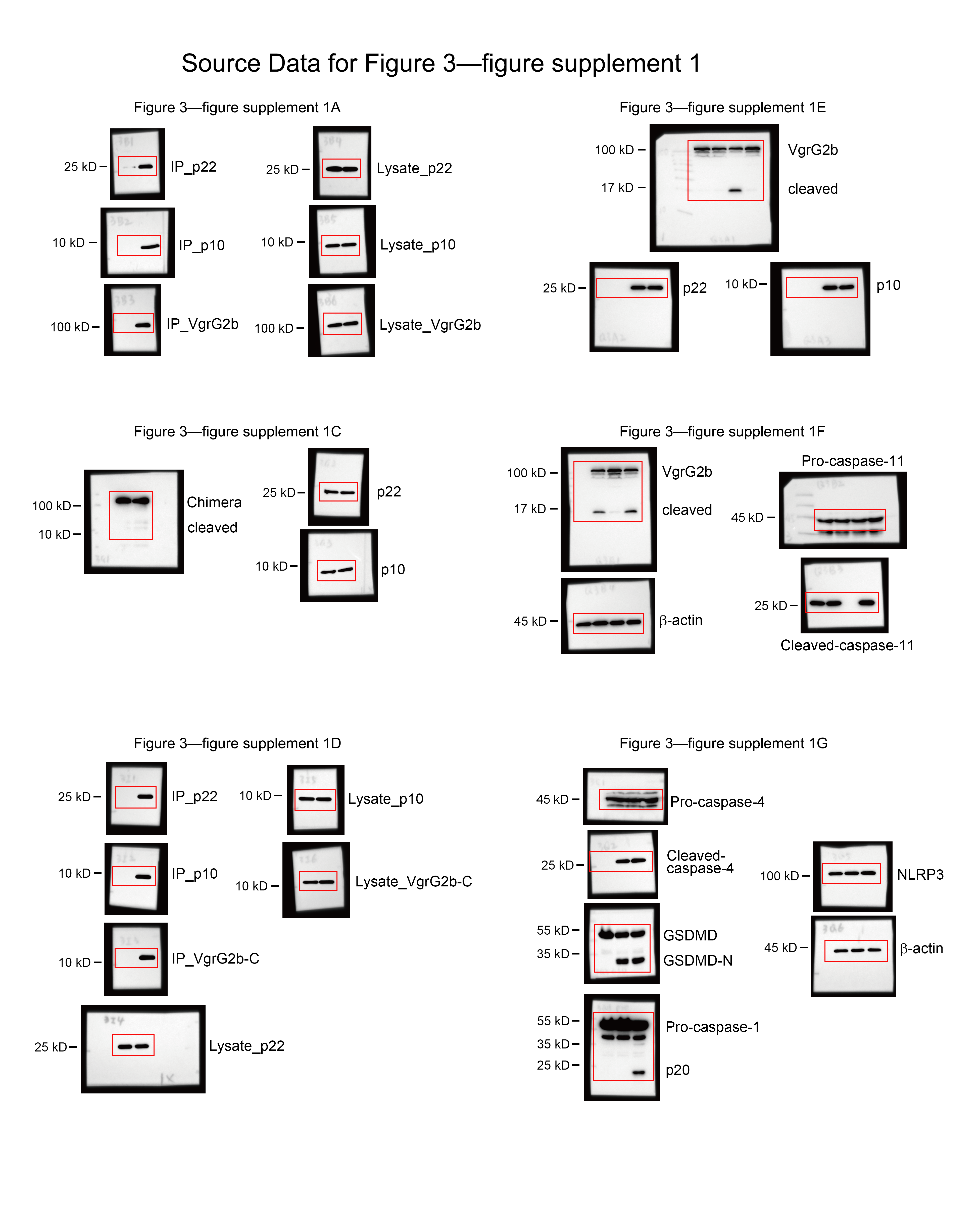

Supplement: Figure 3—figure supplement 1—source data 1. [file elife-99939-fig3-figsupp1-data1.zip › Figure 3-figure supplement 1.tif]

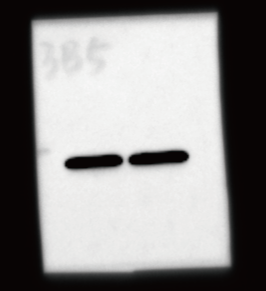

Supplement: Figure 3—figure supplement 1—source data 2. [file elife-99939-fig3-figsupp1-data2.zip › Figure 3-figure supplement 1-source data 2/Figure 3—figure supplement 1A input_p10.tif]

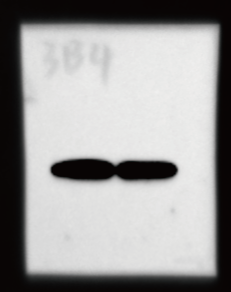

Supplement: Figure 3—figure supplement 1—source data 2. [file elife-99939-fig3-figsupp1-data2.zip › Figure 3-figure supplement 1-source data 2/Figure 3—figure supplement 1A input_p22.tif]

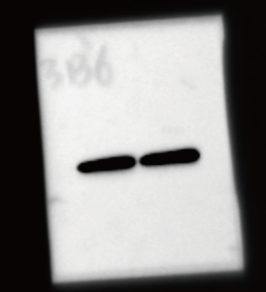

Supplement: Figure 3—figure supplement 1—source data 2. [file elife-99939-fig3-figsupp1-data2.zip › Figure 3-figure supplement 1-source data 2/Figure 3—figure supplement 1A input_VgrG2b.tif]

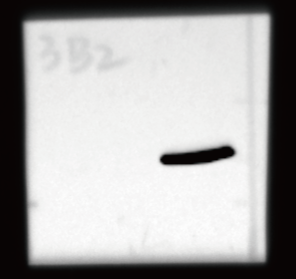

Supplement: Figure 3—figure supplement 1—source data 2. [file elife-99939-fig3-figsupp1-data2.zip › Figure 3-figure supplement 1-source data 2/Figure 3—figure supplement 1A IP_p10.tif]

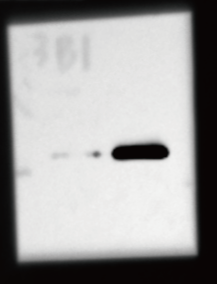

Supplement: Figure 3—figure supplement 1—source data 2. [file elife-99939-fig3-figsupp1-data2.zip › Figure 3-figure supplement 1-source data 2/Figure 3—figure supplement 1A IP_p22.tif]

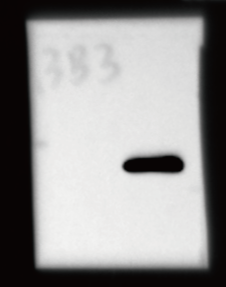

Supplement: Figure 3—figure supplement 1—source data 2. [file elife-99939-fig3-figsupp1-data2.zip › Figure 3-figure supplement 1-source data 2/Figure 3—figure supplement 1A IP_VgrG2b.tif]

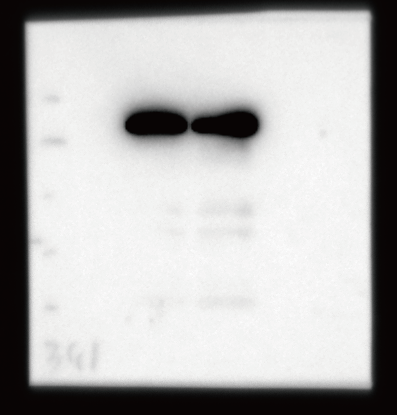

Supplement: Figure 3—figure supplement 1—source data 2. [file elife-99939-fig3-figsupp1-data2.zip › Figure 3-figure supplement 1-source data 2/Figure 3—figure supplement 1C chimera.tif]

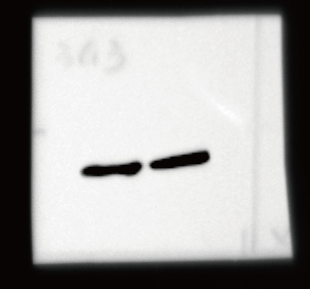

Supplement: Figure 3—figure supplement 1—source data 2. [file elife-99939-fig3-figsupp1-data2.zip › Figure 3-figure supplement 1-source data 2/Figure 3—figure supplement 1C p10.tif]

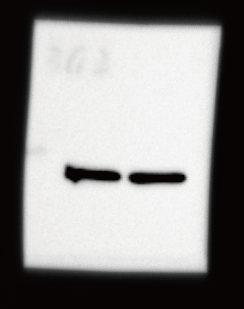

Supplement: Figure 3—figure supplement 1—source data 2. [file elife-99939-fig3-figsupp1-data2.zip › Figure 3-figure supplement 1-source data 2/Figure 3—figure supplement 1C p22.tif]

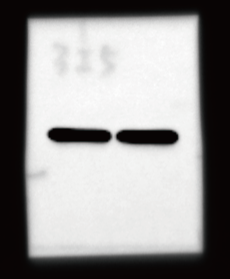

Supplement: Figure 3—figure supplement 1—source data 2. [file elife-99939-fig3-figsupp1-data2.zip › Figure 3-figure supplement 1-source data 2/Figure 3—figure supplement 1D input_p10.tif]

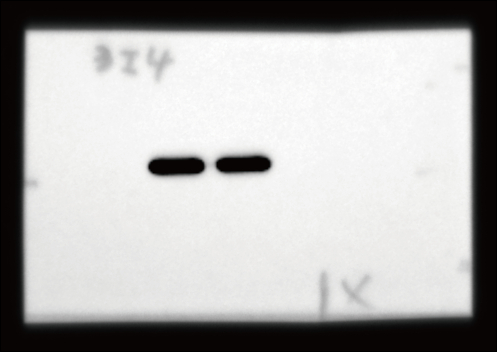

Supplement: Figure 3—figure supplement 1—source data 2. [file elife-99939-fig3-figsupp1-data2.zip › Figure 3-figure supplement 1-source data 2/Figure 3—figure supplement 1D input_p22.tif]

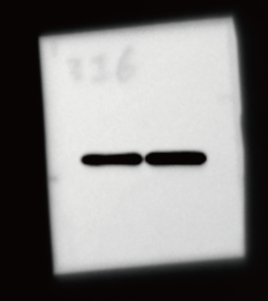

Supplement: Figure 3—figure supplement 1—source data 2. [file elife-99939-fig3-figsupp1-data2.zip › Figure 3-figure supplement 1-source data 2/Figure 3—figure supplement 1D input_VgrG2b-C.tif]

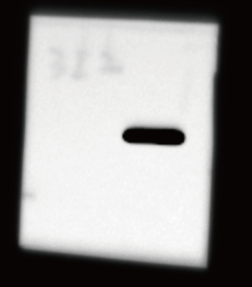

Supplement: Figure 3—figure supplement 1—source data 2. [file elife-99939-fig3-figsupp1-data2.zip › Figure 3-figure supplement 1-source data 2/Figure 3—figure supplement 1D IP_p10.tif]

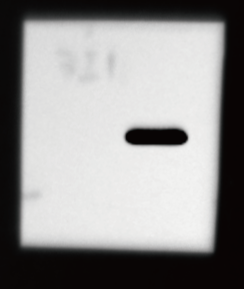

Supplement: Figure 3—figure supplement 1—source data 2. [file elife-99939-fig3-figsupp1-data2.zip › Figure 3-figure supplement 1-source data 2/Figure 3—figure supplement 1D IP_p22.tif]

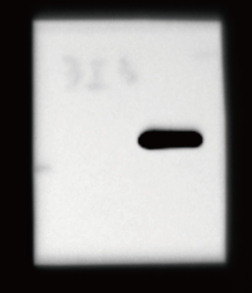

Supplement: Figure 3—figure supplement 1—source data 2. [file elife-99939-fig3-figsupp1-data2.zip › Figure 3-figure supplement 1-source data 2/Figure 3—figure supplement 1D IP_VgrG2b-C.tif]

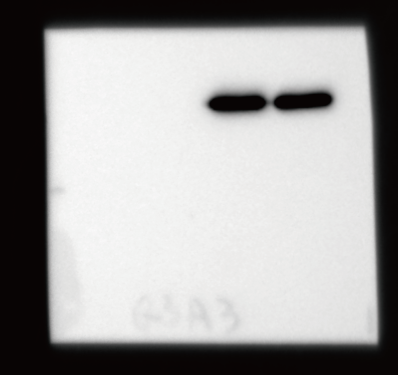

Supplement: Figure 3—figure supplement 1—source data 2. [file elife-99939-fig3-figsupp1-data2.zip › Figure 3-figure supplement 1-source data 2/Figure 3—figure supplement 1E p10.tif]

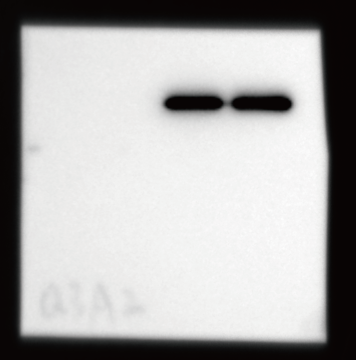

Supplement: Figure 3—figure supplement 1—source data 2. [file elife-99939-fig3-figsupp1-data2.zip › Figure 3-figure supplement 1-source data 2/Figure 3—figure supplement 1E p22.tif]

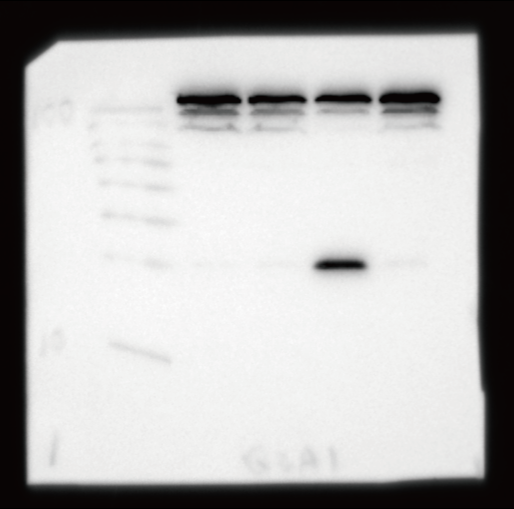

Supplement: Figure 3—figure supplement 1—source data 2. [file elife-99939-fig3-figsupp1-data2.zip › Figure 3-figure supplement 1-source data 2/Figure 3—figure supplement 1E VgrG2b.tif]

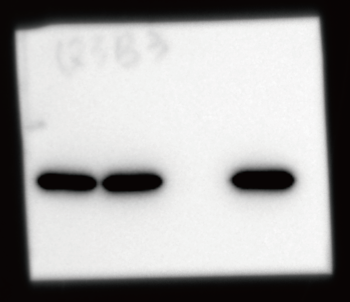

Supplement: Figure 3—figure supplement 1—source data 2. [file elife-99939-fig3-figsupp1-data2.zip › Figure 3-figure supplement 1-source data 2/Figure 3—figure supplement 1F cleaved-caspase-11.tif]

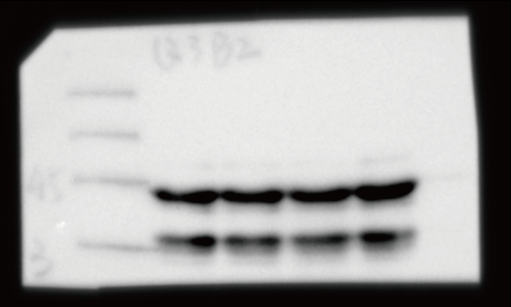

Supplement: Figure 3—figure supplement 1—source data 2. [file elife-99939-fig3-figsupp1-data2.zip › Figure 3-figure supplement 1-source data 2/Figure 3—figure supplement 1F pro-caspase-11.tif]

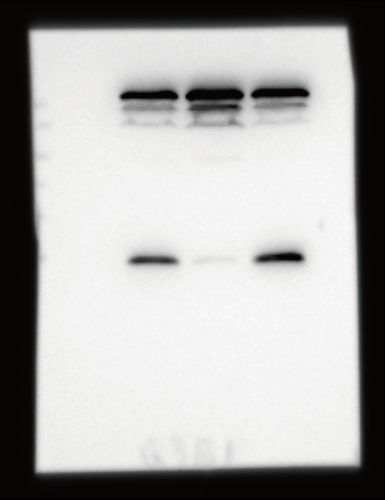

Supplement: Figure 3—figure supplement 1—source data 2. [file elife-99939-fig3-figsupp1-data2.zip › Figure 3-figure supplement 1-source data 2/Figure 3—figure supplement 1F VgrG2b.tif]

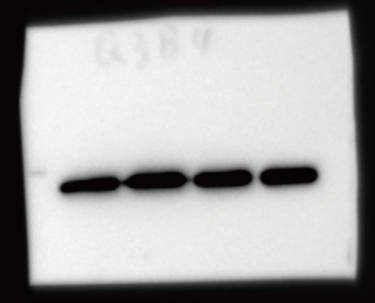

Supplement: Figure 3—figure supplement 1—source data 2. [file elife-99939-fig3-figsupp1-data2.zip › Figure 3-figure supplement 1-source data 2/Figure 3—figure supplement 1F β-actin.tif]

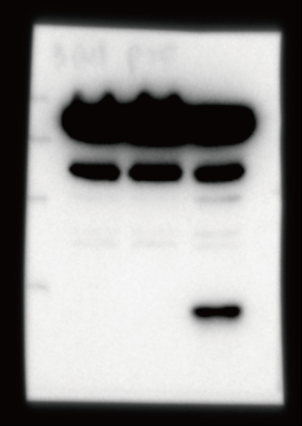

Supplement: Figure 3—figure supplement 1—source data 2. [file elife-99939-fig3-figsupp1-data2.zip › Figure 3-figure supplement 1-source data 2/Figure 3—figure supplement 1G caspase-1.tif]

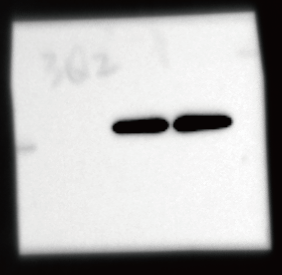

Supplement: Figure 3—figure supplement 1—source data 2. [file elife-99939-fig3-figsupp1-data2.zip › Figure 3-figure supplement 1-source data 2/Figure 3—figure supplement 1G cleaved-caspase-4.tif]

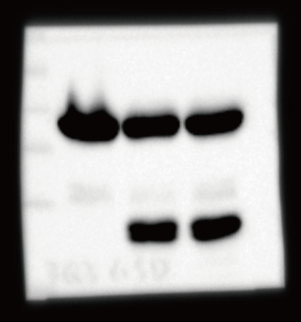

Supplement: Figure 3—figure supplement 1—source data 2. [file elife-99939-fig3-figsupp1-data2.zip › Figure 3-figure supplement 1-source data 2/Figure 3—figure supplement 1G GSDMD.tif]

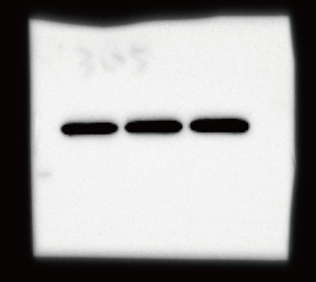

Supplement: Figure 3—figure supplement 1—source data 2. [file elife-99939-fig3-figsupp1-data2.zip › Figure 3-figure supplement 1-source data 2/Figure 3—figure supplement 1G NLRP3.tif]

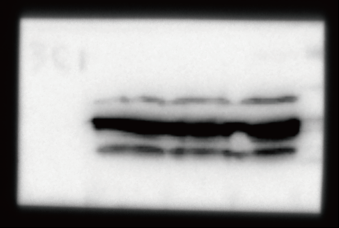

Supplement: Figure 3—figure supplement 1—source data 2. [file elife-99939-fig3-figsupp1-data2.zip › Figure 3-figure supplement 1-source data 2/Figure 3—figure supplement 1G pro-caspase-4.tif]

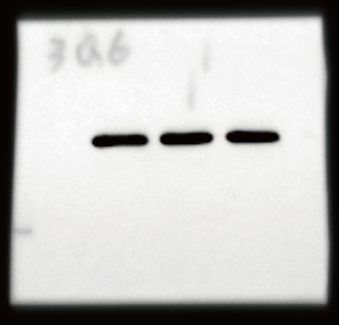

Supplement: Figure 3—figure supplement 1—source data 2. [file elife-99939-fig3-figsupp1-data2.zip › Figure 3-figure supplement 1-source data 2/Figure 3—figure supplement 1G β-actin.tif]

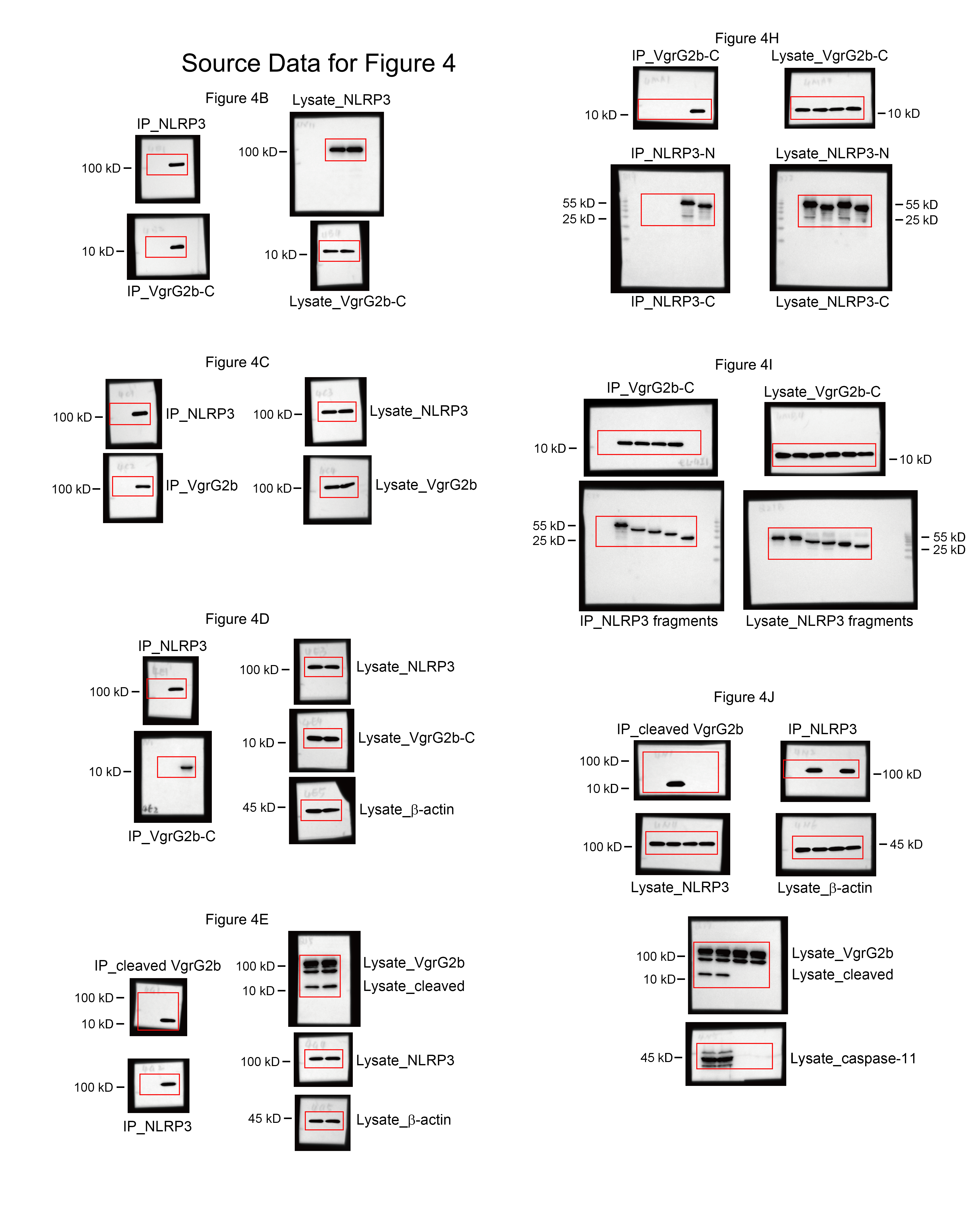

Supplement: Figure 4—source data 1. [file elife-99939-fig4-data1.zip › Figure 4-source data 1.tif]

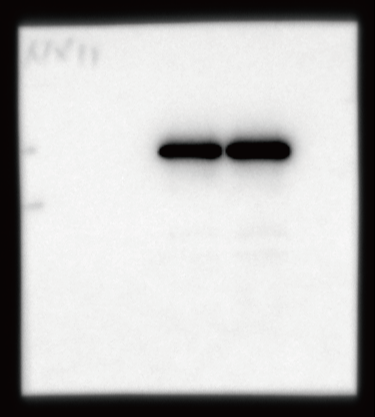

Supplement: Figure 4—source data 2. [file elife-99939-fig4-data2.zip › Figure 4-source data 2/Figure 4B input_NLRP3.tif]

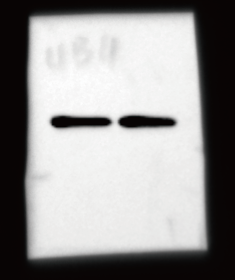

Supplement: Figure 4—source data 2. [file elife-99939-fig4-data2.zip › Figure 4-source data 2/Figure 4B input_VgrG2b-C.tif]

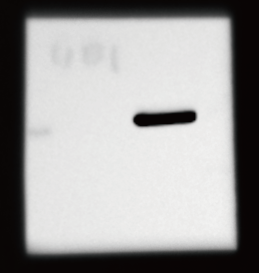

Supplement: Figure 4—source data 2. [file elife-99939-fig4-data2.zip › Figure 4-source data 2/Figure 4B IP_NLRP3.tif]

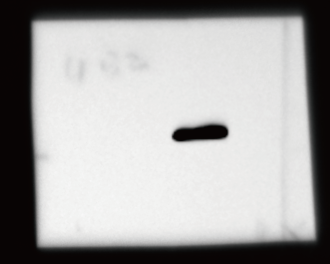

Supplement: Figure 4—source data 2. [file elife-99939-fig4-data2.zip › Figure 4-source data 2/Figure 4B IP_VgrG2b-C.tif]

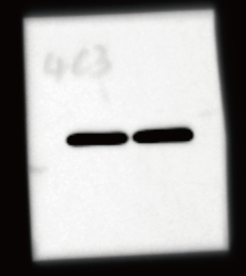

Supplement: Figure 4—source data 2. [file elife-99939-fig4-data2.zip › Figure 4-source data 2/Figure 4C input_NLRP3.tif]

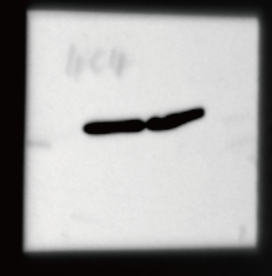

Supplement: Figure 4—source data 2. [file elife-99939-fig4-data2.zip › Figure 4-source data 2/Figure 4C input_VgrG2b.tif]

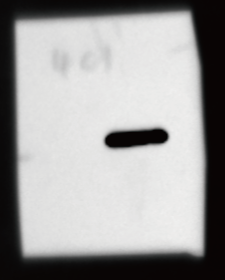

Supplement: Figure 4—source data 2. [file elife-99939-fig4-data2.zip › Figure 4-source data 2/Figure 4C IP_NLRP3.tif]

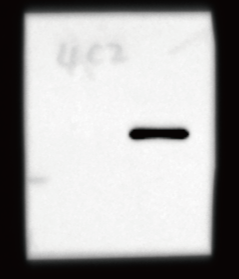

Supplement: Figure 4—source data 2. [file elife-99939-fig4-data2.zip › Figure 4-source data 2/Figure 4C IP_VgrG2b.tif]

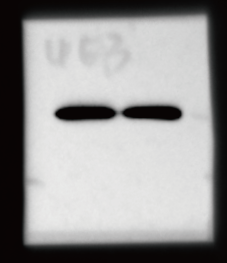

Supplement: Figure 4—source data 2. [file elife-99939-fig4-data2.zip › Figure 4-source data 2/Figure 4D input_NLRP3.tif]

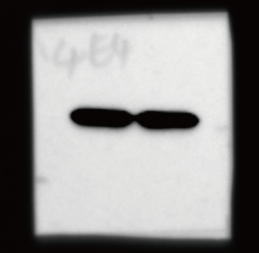

Supplement: Figure 4—source data 2. [file elife-99939-fig4-data2.zip › Figure 4-source data 2/Figure 4D input_VgrG2b-C.tif]

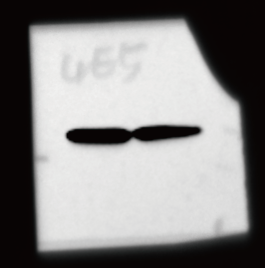

Supplement: Figure 4—source data 2. [file elife-99939-fig4-data2.zip › Figure 4-source data 2/Figure 4D input_β-actin.tif]

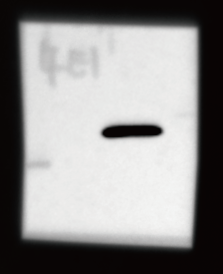

Supplement: Figure 4—source data 2. [file elife-99939-fig4-data2.zip › Figure 4-source data 2/Figure 4D IP_NLRP3.tif]

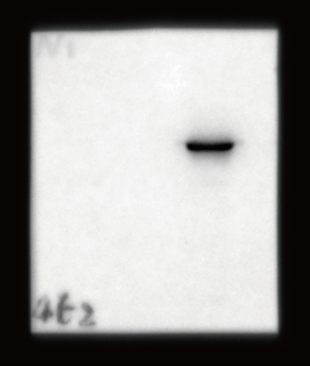

Supplement: Figure 4—source data 2. [file elife-99939-fig4-data2.zip › Figure 4-source data 2/Figure 4D IP_VgrG2b-C.tif]

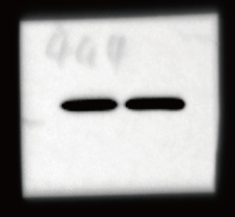

Supplement: Figure 4—source data 2. [file elife-99939-fig4-data2.zip › Figure 4-source data 2/Figure 4E input_NLRP3.tif]

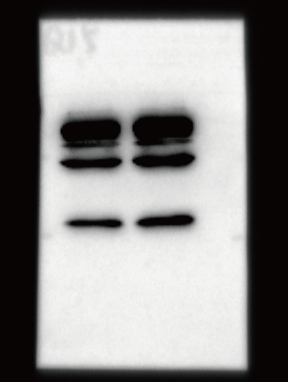

Supplement: Figure 4—source data 2. [file elife-99939-fig4-data2.zip › Figure 4-source data 2/Figure 4E input_VgrG2b.tif]

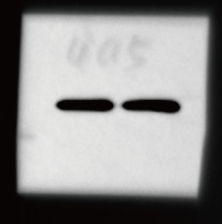

Supplement: Figure 4—source data 2. [file elife-99939-fig4-data2.zip › Figure 4-source data 2/Figure 4E input_β-actin.tif]

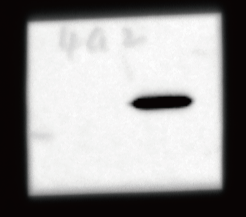

Supplement: Figure 4—source data 2. [file elife-99939-fig4-data2.zip › Figure 4-source data 2/Figure 4E IP_NLRP3.tif]

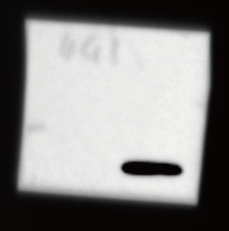

Supplement: Figure 4—source data 2. [file elife-99939-fig4-data2.zip › Figure 4-source data 2/Figure 4E IP_VgrG2b.tif]

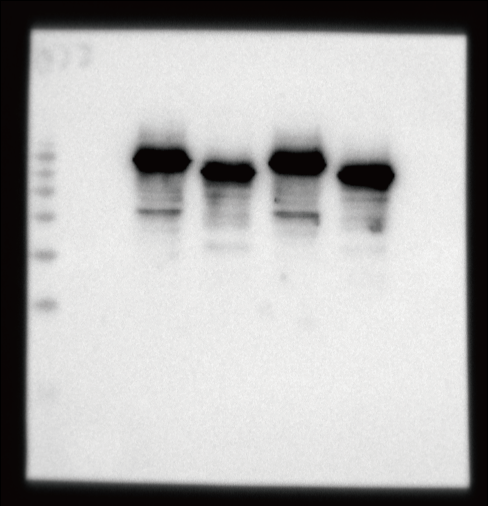

Supplement: Figure 4—source data 2. [file elife-99939-fig4-data2.zip › Figure 4-source data 2/Figure 4H input_NLRP3.tif]

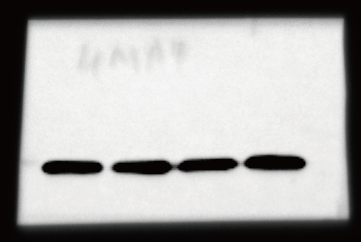

Supplement: Figure 4—source data 2. [file elife-99939-fig4-data2.zip › Figure 4-source data 2/Figure 4H input_VgrG2b-C.tif]

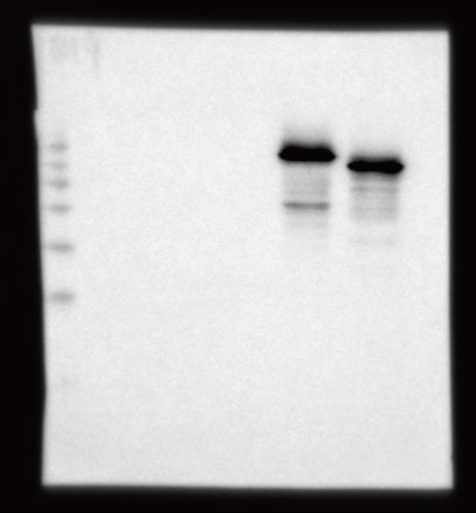

Supplement: Figure 4—source data 2. [file elife-99939-fig4-data2.zip › Figure 4-source data 2/Figure 4H IP_NLRP3.tif]

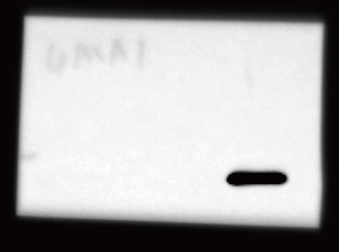

Supplement: Figure 4—source data 2. [file elife-99939-fig4-data2.zip › Figure 4-source data 2/Figure 4H IP_VgrG2b-C.tif]

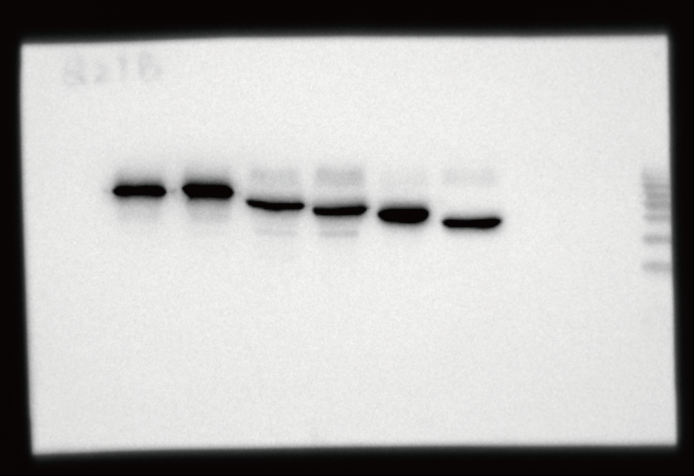

Supplement: Figure 4—source data 2. [file elife-99939-fig4-data2.zip › Figure 4-source data 2/Figure 4I input_NLRP3.tif]

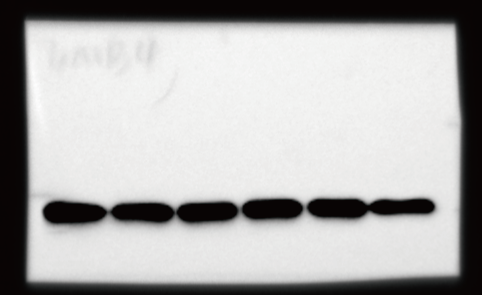

Supplement: Figure 4—source data 2. [file elife-99939-fig4-data2.zip › Figure 4-source data 2/Figure 4I input_VgrG2b-C.tif]

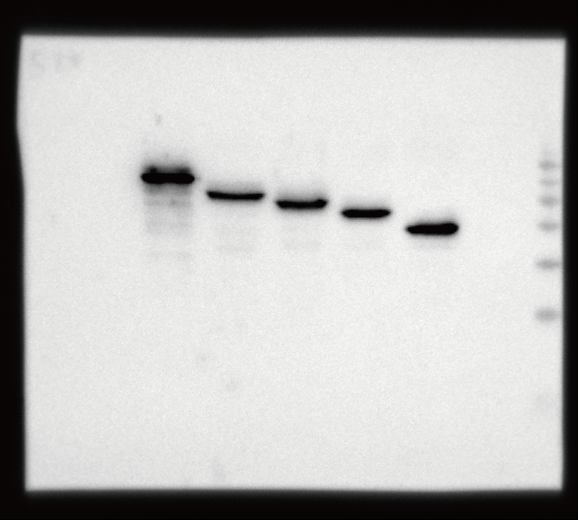

Supplement: Figure 4—source data 2. [file elife-99939-fig4-data2.zip › Figure 4-source data 2/Figure 4I IP_NLRP3.tif]

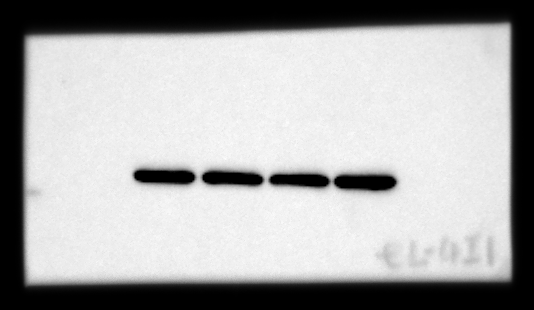

Supplement: Figure 4—source data 2. [file elife-99939-fig4-data2.zip › Figure 4-source data 2/Figure 4I IP_VgrG2b-C.tif]

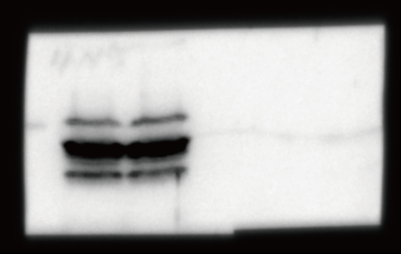

Supplement: Figure 4—source data 2. [file elife-99939-fig4-data2.zip › Figure 4-source data 2/Figure 4J input_caspase-11.tif]

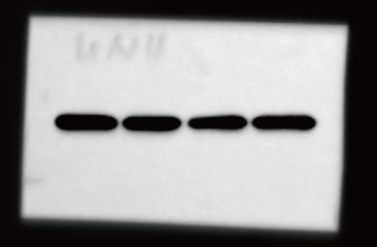

Supplement: Figure 4—source data 2. [file elife-99939-fig4-data2.zip › Figure 4-source data 2/Figure 4J input_NLRP3.tif]

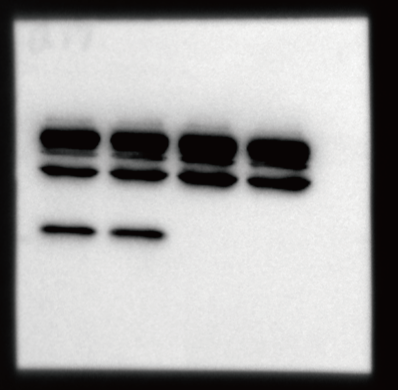

Supplement: Figure 4—source data 2. [file elife-99939-fig4-data2.zip › Figure 4-source data 2/Figure 4J input_VgrG2b.tif]
