# Supplementary figures and images for: A VgrG2b fragment cleaved by caspase-11/4 promotes Pseudomonas aeruginosa infection through suppressing the NLRP3 inflammasome (part 3 of 4)
Source: eLife. 2025 Feb 25;13:RP99939. doi: 10.7554/eLife.99939 (PMC11856931; doi:10.7554/eLife.99939)

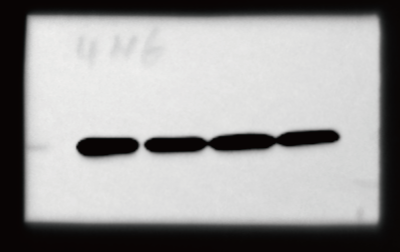

Supplement: Figure 4—source data 2. [file elife-99939-fig4-data2.zip › Figure 4-source data 2/Figure 4J input_β-actin.tif]

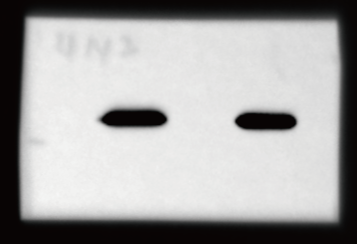

Supplement: Figure 4—source data 2. [file elife-99939-fig4-data2.zip › Figure 4-source data 2/Figure 4J IP_NLRP3.tif]

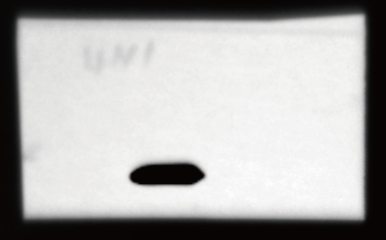

Supplement: Figure 4—source data 2. [file elife-99939-fig4-data2.zip › Figure 4-source data 2/Figure 4J IP_VgrG2b.tif]

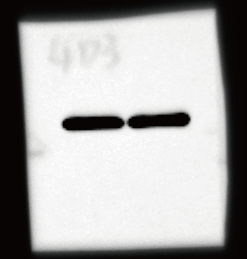

Supplement: Figure 4—figure supplement 1—source data 2. [file elife-99939-fig4-figsupp1-data2.zip › Figure 4-figure supplement 1-source data 2/Figure 4—figure supplement 1A input_NLRP3.tif]

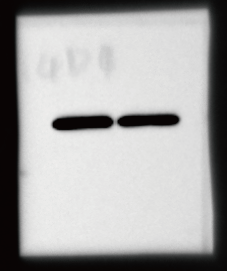

Supplement: Figure 4—figure supplement 1—source data 2. [file elife-99939-fig4-figsupp1-data2.zip › Figure 4-figure supplement 1-source data 2/Figure 4—figure supplement 1A input_VgrG2b-N.tif]

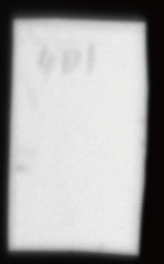

Supplement: Figure 4—figure supplement 1—source data 2. [file elife-99939-fig4-figsupp1-data2.zip › Figure 4-figure supplement 1-source data 2/Figure 4—figure supplement 1A IP_NLRP3.tif]

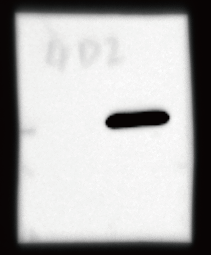

Supplement: Figure 4—figure supplement 1—source data 2. [file elife-99939-fig4-figsupp1-data2.zip › Figure 4-figure supplement 1-source data 2/Figure 4—figure supplement 1A IP_VgrG2b-N.tif]

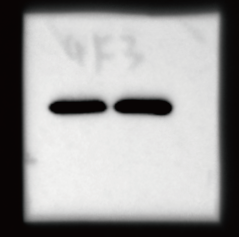

Supplement: Figure 4—figure supplement 1—source data 2. [file elife-99939-fig4-figsupp1-data2.zip › Figure 4-figure supplement 1-source data 2/Figure 4—figure supplement 1B input_NLRP3.tif]

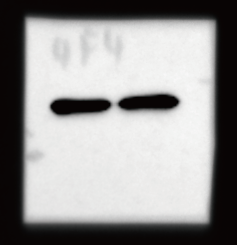

Supplement: Figure 4—figure supplement 1—source data 2. [file elife-99939-fig4-figsupp1-data2.zip › Figure 4-figure supplement 1-source data 2/Figure 4—figure supplement 1B input_VgrG2b-C.tif]

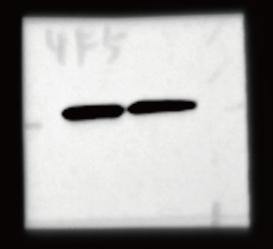

Supplement: Figure 4—figure supplement 1—source data 2. [file elife-99939-fig4-figsupp1-data2.zip › Figure 4-figure supplement 1-source data 2/Figure 4—figure supplement 1B input_β-actin.tif]

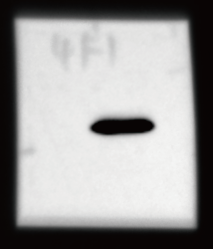

Supplement: Figure 4—figure supplement 1—source data 2. [file elife-99939-fig4-figsupp1-data2.zip › Figure 4-figure supplement 1-source data 2/Figure 4—figure supplement 1B IP_NLRP3.tif]

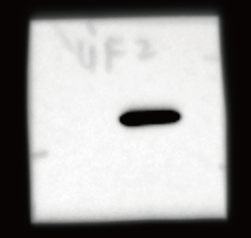

Supplement: Figure 4—figure supplement 1—source data 2. [file elife-99939-fig4-figsupp1-data2.zip › Figure 4-figure supplement 1-source data 2/Figure 4—figure supplement 1B IP_VgrG2b-C.tif]

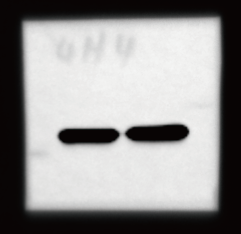

Supplement: Figure 4—figure supplement 1—source data 2. [file elife-99939-fig4-figsupp1-data2.zip › Figure 4-figure supplement 1-source data 2/Figure 4—figure supplement 1C input_NLRP3.tif]

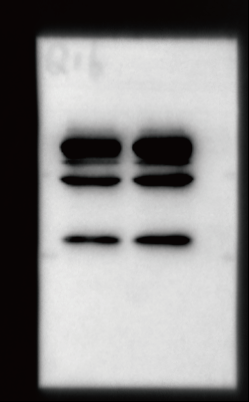

Supplement: Figure 4—figure supplement 1—source data 2. [file elife-99939-fig4-figsupp1-data2.zip › Figure 4-figure supplement 1-source data 2/Figure 4—figure supplement 1C input_VgrG2b.tif]

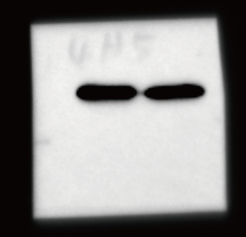

Supplement: Figure 4—figure supplement 1—source data 2. [file elife-99939-fig4-figsupp1-data2.zip › Figure 4-figure supplement 1-source data 2/Figure 4—figure supplement 1C input_β-actin.tif]

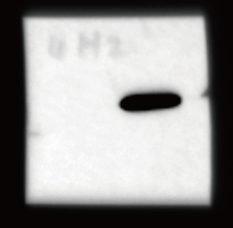

Supplement: Figure 4—figure supplement 1—source data 2. [file elife-99939-fig4-figsupp1-data2.zip › Figure 4-figure supplement 1-source data 2/Figure 4—figure supplement 1C IP_NLRP3.tif]

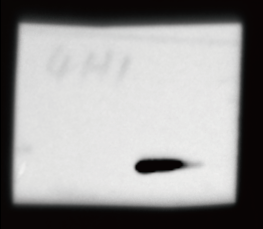

Supplement: Figure 4—figure supplement 1—source data 2. [file elife-99939-fig4-figsupp1-data2.zip › Figure 4-figure supplement 1-source data 2/Figure 4—figure supplement 1C IP_VgrG2b.tif]

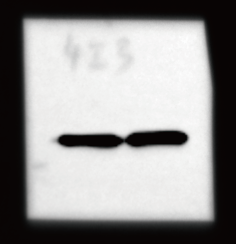

Supplement: Figure 4—figure supplement 1—source data 2. [file elife-99939-fig4-figsupp1-data2.zip › Figure 4-figure supplement 1-source data 2/Figure 4—figure supplement 1D input_GSDMD.tif]

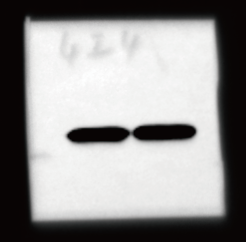

Supplement: Figure 4—figure supplement 1—source data 2. [file elife-99939-fig4-figsupp1-data2.zip › Figure 4-figure supplement 1-source data 2/Figure 4—figure supplement 1D input_VgrG2b-C.tif]

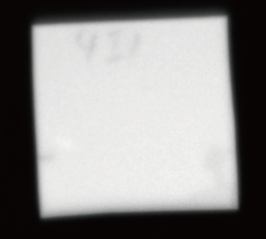

Supplement: Figure 4—figure supplement 1—source data 2. [file elife-99939-fig4-figsupp1-data2.zip › Figure 4-figure supplement 1-source data 2/Figure 4—figure supplement 1D IP_GSDMD.tif]

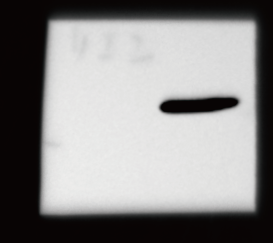

Supplement: Figure 4—figure supplement 1—source data 2. [file elife-99939-fig4-figsupp1-data2.zip › Figure 4-figure supplement 1-source data 2/Figure 4—figure supplement 1D IP_VgrG2b-C.tif]

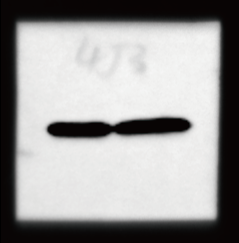

Supplement: Figure 4—figure supplement 1—source data 2. [file elife-99939-fig4-figsupp1-data2.zip › Figure 4-figure supplement 1-source data 2/Figure 4—figure supplement 1E input_ASC.tif]

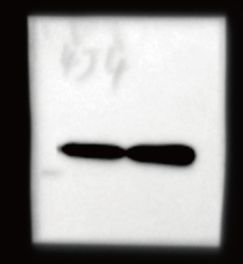

Supplement: Figure 4—figure supplement 1—source data 2. [file elife-99939-fig4-figsupp1-data2.zip › Figure 4-figure supplement 1-source data 2/Figure 4—figure supplement 1E input_VgrG2b-C.tif]

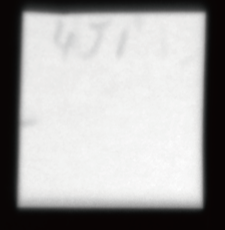

Supplement: Figure 4—figure supplement 1—source data 2. [file elife-99939-fig4-figsupp1-data2.zip › Figure 4-figure supplement 1-source data 2/Figure 4—figure supplement 1E IP_ASC.tif]

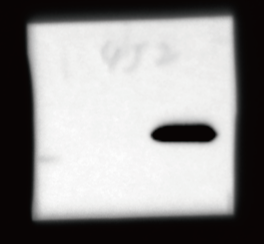

Supplement: Figure 4—figure supplement 1—source data 2. [file elife-99939-fig4-figsupp1-data2.zip › Figure 4-figure supplement 1-source data 2/Figure 4—figure supplement 1E IP_VgrG2b-C.tif]

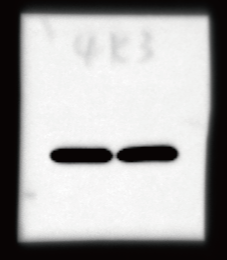

Supplement: Figure 4—figure supplement 1—source data 2. [file elife-99939-fig4-figsupp1-data2.zip › Figure 4-figure supplement 1-source data 2/Figure 4—figure supplement 1F input_caspase-1.tif]

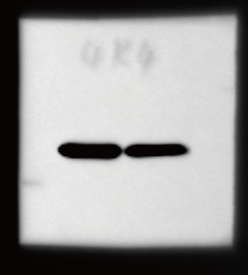

Supplement: Figure 4—figure supplement 1—source data 2. [file elife-99939-fig4-figsupp1-data2.zip › Figure 4-figure supplement 1-source data 2/Figure 4—figure supplement 1F input_VgrG2b-C.tif]

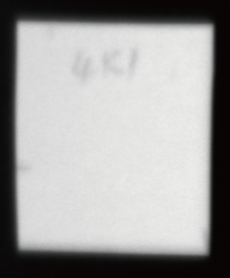

Supplement: Figure 4—figure supplement 1—source data 2. [file elife-99939-fig4-figsupp1-data2.zip › Figure 4-figure supplement 1-source data 2/Figure 4—figure supplement 1F IP_caspase-1.tif]

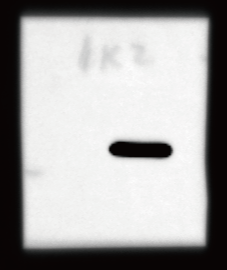

Supplement: Figure 4—figure supplement 1—source data 2. [file elife-99939-fig4-figsupp1-data2.zip › Figure 4-figure supplement 1-source data 2/Figure 4—figure supplement 1F IP_VgrG2b-C.tif]

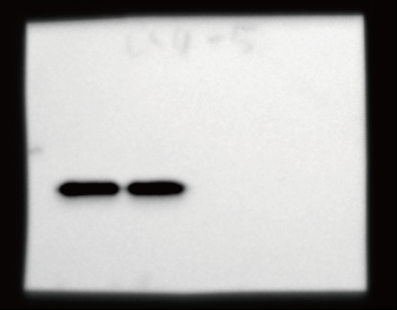

Supplement: Figure 4—figure supplement 1—source data 2. [file elife-99939-fig4-figsupp1-data2.zip › Figure 4-figure supplement 1-source data 2/Figure 4—figure supplement 1G input_p10.tif]

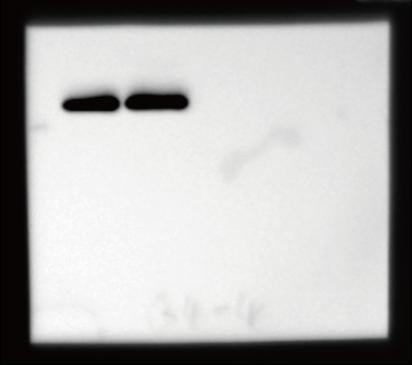

Supplement: Figure 4—figure supplement 1—source data 2. [file elife-99939-fig4-figsupp1-data2.zip › Figure 4-figure supplement 1-source data 2/Figure 4—figure supplement 1G input_p20.tif]

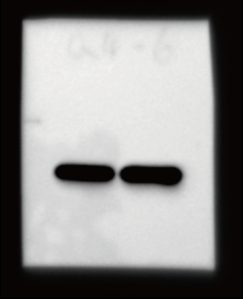

Supplement: Figure 4—figure supplement 1—source data 2. [file elife-99939-fig4-figsupp1-data2.zip › Figure 4-figure supplement 1-source data 2/Figure 4—figure supplement 1G input_VgrG2b-C.tif]

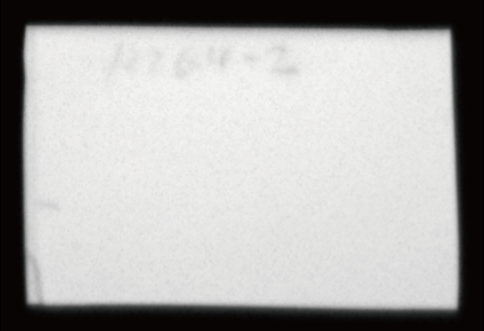

Supplement: Figure 4—figure supplement 1—source data 2. [file elife-99939-fig4-figsupp1-data2.zip › Figure 4-figure supplement 1-source data 2/Figure 4—figure supplement 1G IP_p10.tif]

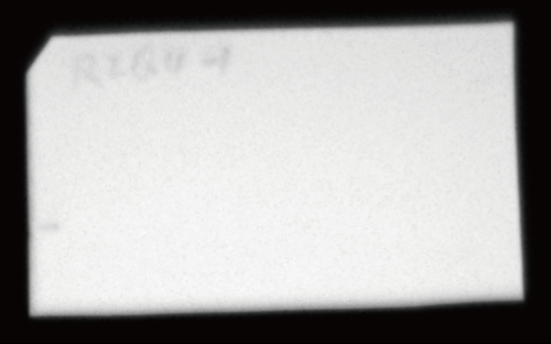

Supplement: Figure 4—figure supplement 1—source data 2. [file elife-99939-fig4-figsupp1-data2.zip › Figure 4-figure supplement 1-source data 2/Figure 4—figure supplement 1G IP_p20.tif]

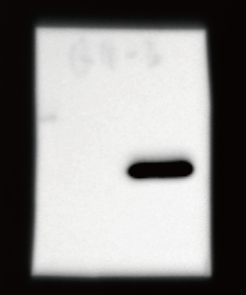

Supplement: Figure 4—figure supplement 1—source data 2. [file elife-99939-fig4-figsupp1-data2.zip › Figure 4-figure supplement 1-source data 2/Figure 4—figure supplement 1G IP_VgrG2b-C.tif]

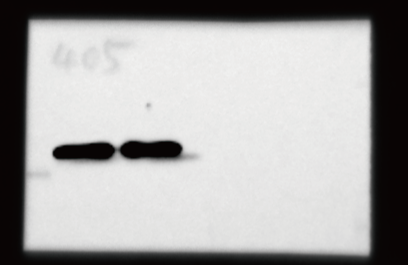

Supplement: Figure 4—figure supplement 1—source data 2. [file elife-99939-fig4-figsupp1-data2.zip › Figure 4-figure supplement 1-source data 2/Figure 4—figure supplement 1H input_GSDMD.tif]

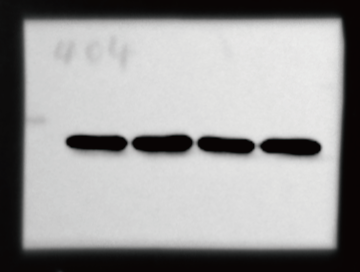

Supplement: Figure 4—figure supplement 1—source data 2. [file elife-99939-fig4-figsupp1-data2.zip › Figure 4-figure supplement 1-source data 2/Figure 4—figure supplement 1H input_NLRP3.tif]

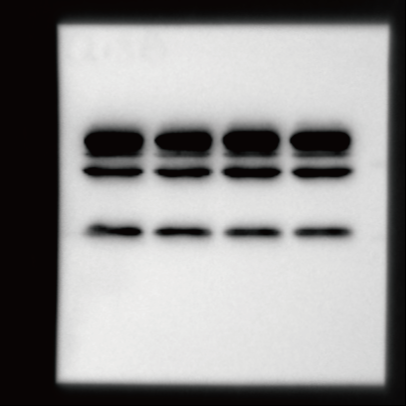

Supplement: Figure 4—figure supplement 1—source data 2. [file elife-99939-fig4-figsupp1-data2.zip › Figure 4-figure supplement 1-source data 2/Figure 4—figure supplement 1H input_VgrG2b.tif]

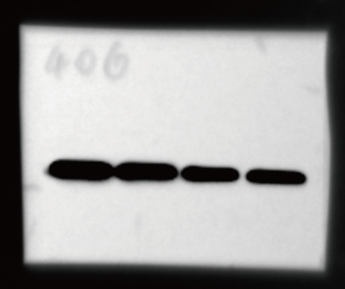

Supplement: Figure 4—figure supplement 1—source data 2. [file elife-99939-fig4-figsupp1-data2.zip › Figure 4-figure supplement 1-source data 2/Figure 4—figure supplement 1H input_β-actin.tif]

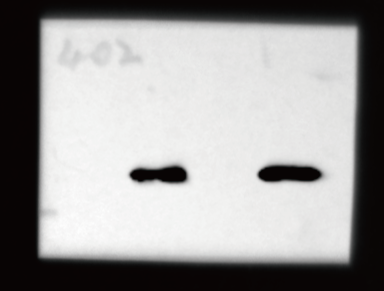

Supplement: Figure 4—figure supplement 1—source data 2. [file elife-99939-fig4-figsupp1-data2.zip › Figure 4-figure supplement 1-source data 2/Figure 4—figure supplement 1H IP_NLRP3.tif]

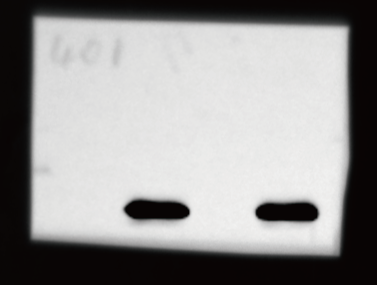

Supplement: Figure 4—figure supplement 1—source data 2. [file elife-99939-fig4-figsupp1-data2.zip › Figure 4-figure supplement 1-source data 2/Figure 4—figure supplement 1H IP_VgrG2b.tif]

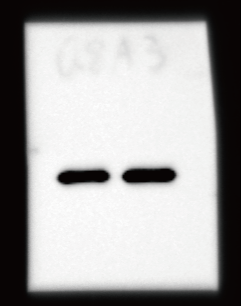

Supplement: Figure 4—figure supplement 1—source data 2. [file elife-99939-fig4-figsupp1-data2.zip › Figure 4-figure supplement 1-source data 2/Figure 4—figure supplement 1I p10.tif]

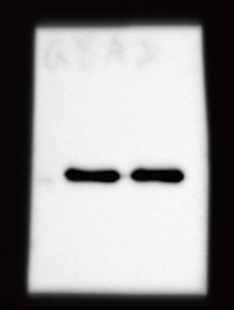

Supplement: Figure 4—figure supplement 1—source data 2. [file elife-99939-fig4-figsupp1-data2.zip › Figure 4-figure supplement 1-source data 2/Figure 4—figure supplement 1I p22.tif]

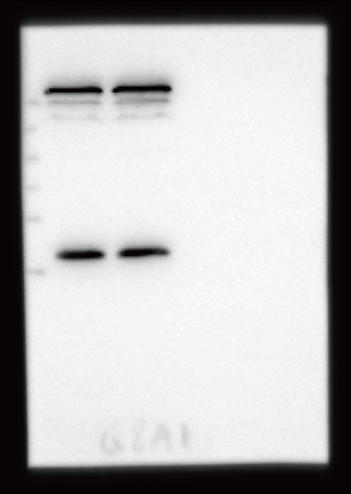

Supplement: Figure 4—figure supplement 1—source data 2. [file elife-99939-fig4-figsupp1-data2.zip › Figure 4-figure supplement 1-source data 2/Figure 4—figure supplement 1I VgrG2b.tif]

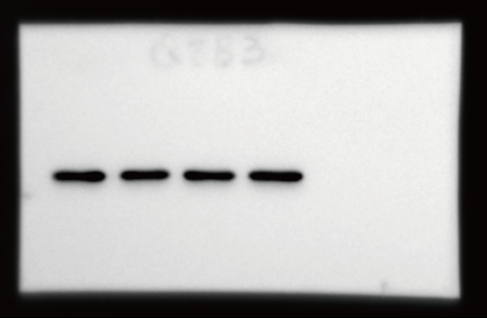

Supplement: Figure 4—figure supplement 1—source data 2. [file elife-99939-fig4-figsupp1-data2.zip › Figure 4-figure supplement 1-source data 2/Figure 4—figure supplement 1J input_NLRP3.tif]

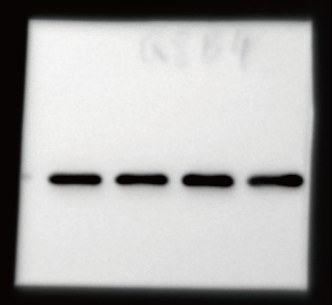

Supplement: Figure 4—figure supplement 1—source data 2. [file elife-99939-fig4-figsupp1-data2.zip › Figure 4-figure supplement 1-source data 2/Figure 4—figure supplement 1J input_VgrG2b-C.tif]

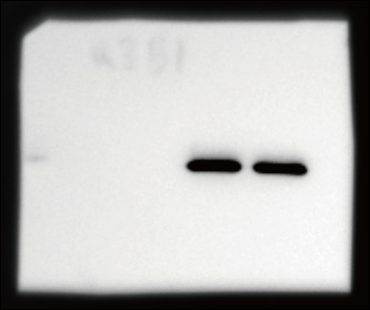

Supplement: Figure 4—figure supplement 1—source data 2. [file elife-99939-fig4-figsupp1-data2.zip › Figure 4-figure supplement 1-source data 2/Figure 4—figure supplement 1J IP_NLRP3.tif]

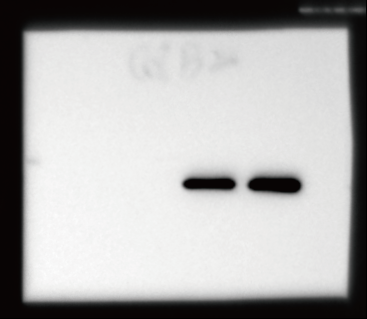

Supplement: Figure 4—figure supplement 1—source data 2. [file elife-99939-fig4-figsupp1-data2.zip › Figure 4-figure supplement 1-source data 2/Figure 4—figure supplement 1J IP_VgrG2b-C.tif]

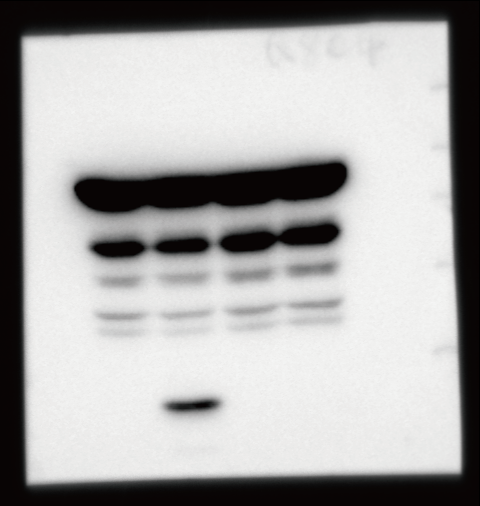

Supplement: Figure 4—figure supplement 1—source data 2. [file elife-99939-fig4-figsupp1-data2.zip › Figure 4-figure supplement 1-source data 2/Figure 4—figure supplement 1K caspase-1.tif]

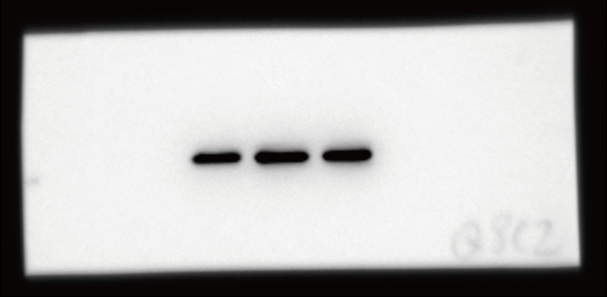

Supplement: Figure 4—figure supplement 1—source data 2. [file elife-99939-fig4-figsupp1-data2.zip › Figure 4-figure supplement 1-source data 2/Figure 4—figure supplement 1K cleaved-caspase-11.tif]

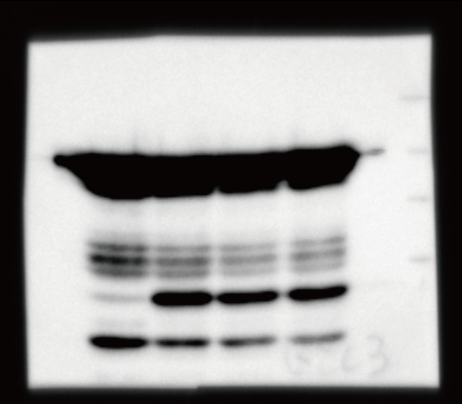

Supplement: Figure 4—figure supplement 1—source data 2. [file elife-99939-fig4-figsupp1-data2.zip › Figure 4-figure supplement 1-source data 2/Figure 4—figure supplement 1K GSDMD.tif]

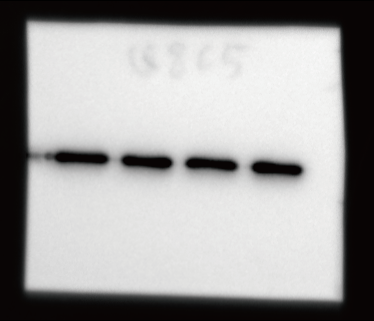

Supplement: Figure 4—figure supplement 1—source data 2. [file elife-99939-fig4-figsupp1-data2.zip › Figure 4-figure supplement 1-source data 2/Figure 4—figure supplement 1K NLRP3.tif]

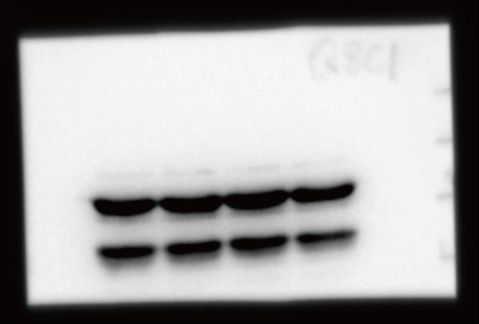

Supplement: Figure 4—figure supplement 1—source data 2. [file elife-99939-fig4-figsupp1-data2.zip › Figure 4-figure supplement 1-source data 2/Figure 4—figure supplement 1K pro-caspase-11.tif]

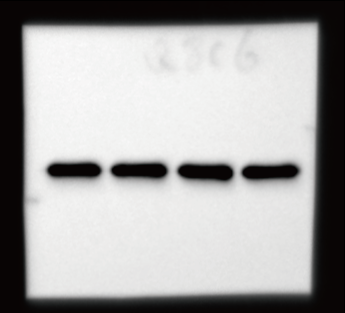

Supplement: Figure 4—figure supplement 1—source data 2. [file elife-99939-fig4-figsupp1-data2.zip › Figure 4-figure supplement 1-source data 2/Figure 4—figure supplement 1K β-actin.tif]

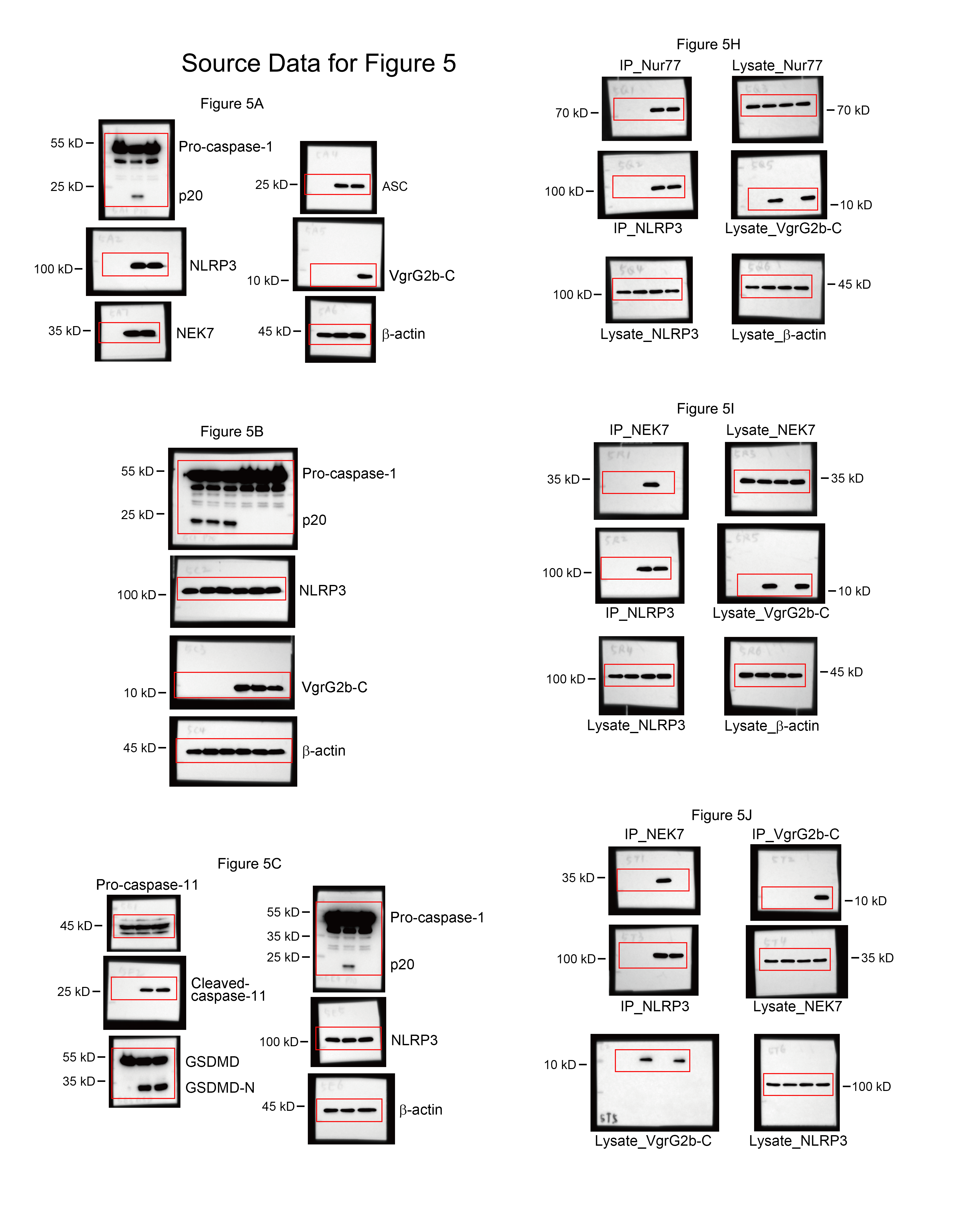

Supplement: Figure 5—source data 1. [file elife-99939-fig5-data1.zip › Figure 5-source data 1.tif]

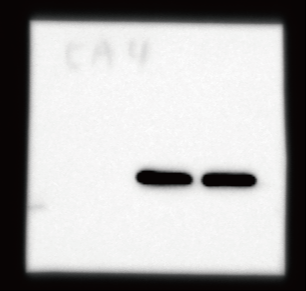

Supplement: Figure 5—source data 2. [file elife-99939-fig5-data2.zip › Figure 5-source data 2/Figure 5A ASC.tif]

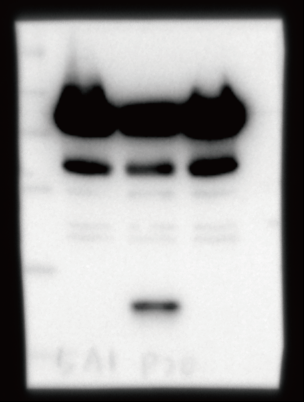

Supplement: Figure 5—source data 2. [file elife-99939-fig5-data2.zip › Figure 5-source data 2/Figure 5A caspase-1.tif]

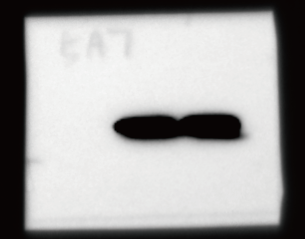

Supplement: Figure 5—source data 2. [file elife-99939-fig5-data2.zip › Figure 5-source data 2/Figure 5A NEK7.tif]

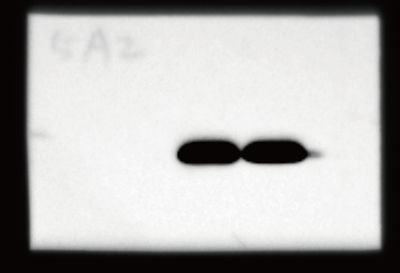

Supplement: Figure 5—source data 2. [file elife-99939-fig5-data2.zip › Figure 5-source data 2/Figure 5A NLRP3.tif]

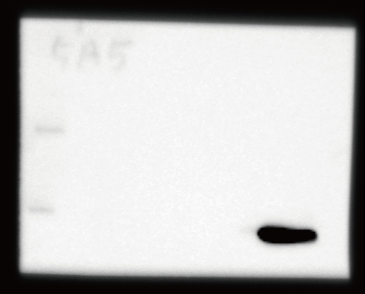

Supplement: Figure 5—source data 2. [file elife-99939-fig5-data2.zip › Figure 5-source data 2/Figure 5A VgrG2b-C.tif]

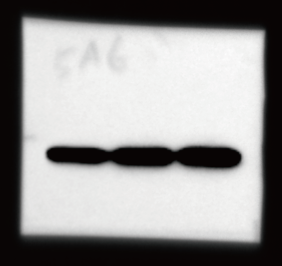

Supplement: Figure 5—source data 2. [file elife-99939-fig5-data2.zip › Figure 5-source data 2/Figure 5A β-actin.tif]

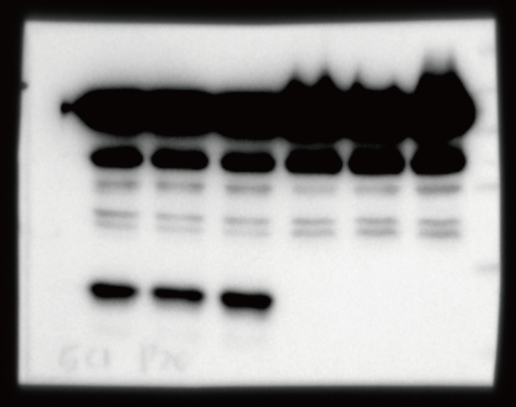

Supplement: Figure 5—source data 2. [file elife-99939-fig5-data2.zip › Figure 5-source data 2/Figure 5B caspase-1.tif]

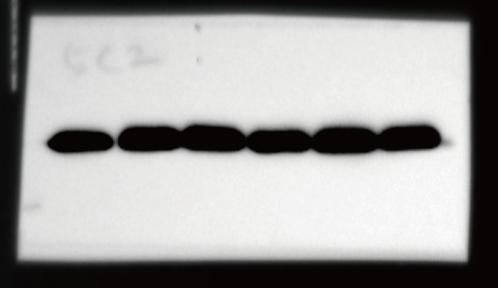

Supplement: Figure 5—source data 2. [file elife-99939-fig5-data2.zip › Figure 5-source data 2/Figure 5B NLRP3.tif]

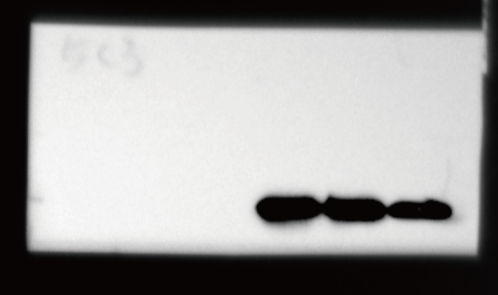

Supplement: Figure 5—source data 2. [file elife-99939-fig5-data2.zip › Figure 5-source data 2/Figure 5B VgrG2b-C.tif]

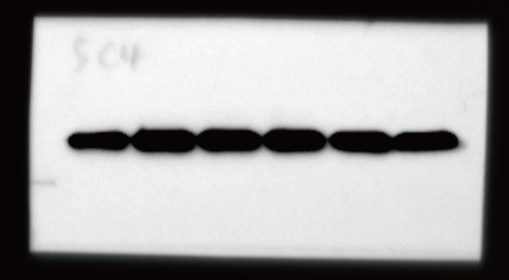

Supplement: Figure 5—source data 2. [file elife-99939-fig5-data2.zip › Figure 5-source data 2/Figure 5B β-actin.tif]

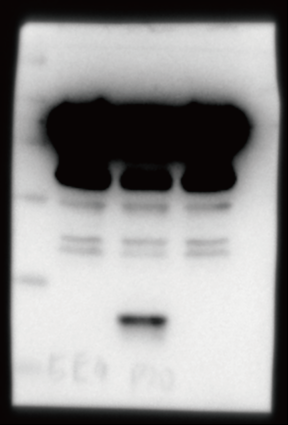

Supplement: Figure 5—source data 2. [file elife-99939-fig5-data2.zip › Figure 5-source data 2/Figure 5C caspase-1.tif]

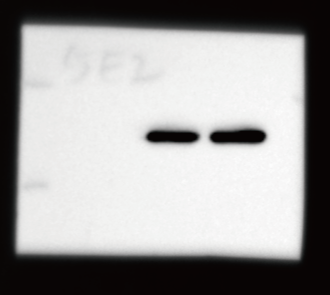

Supplement: Figure 5—source data 2. [file elife-99939-fig5-data2.zip › Figure 5-source data 2/Figure 5C cleaved-caspase-11.tif]

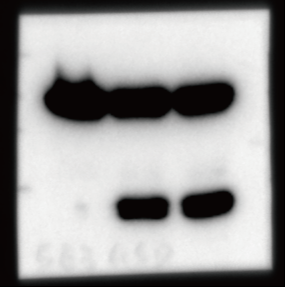

Supplement: Figure 5—source data 2. [file elife-99939-fig5-data2.zip › Figure 5-source data 2/Figure 5C GSDMD.tif]

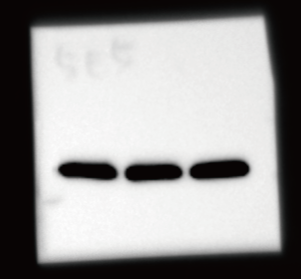

Supplement: Figure 5—source data 2. [file elife-99939-fig5-data2.zip › Figure 5-source data 2/Figure 5C NLRP3.tif]

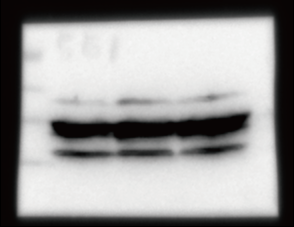

Supplement: Figure 5—source data 2. [file elife-99939-fig5-data2.zip › Figure 5-source data 2/Figure 5C pro-caspase-11.tif]

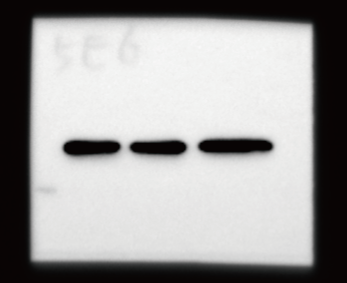

Supplement: Figure 5—source data 2. [file elife-99939-fig5-data2.zip › Figure 5-source data 2/Figure 5C β-actin.tif]

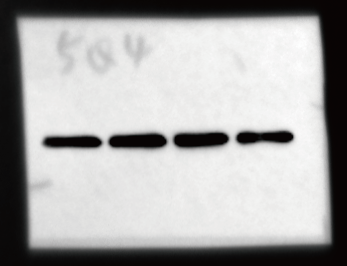

Supplement: Figure 5—source data 2. [file elife-99939-fig5-data2.zip › Figure 5-source data 2/Figure 5H input_NLRP3.tif]

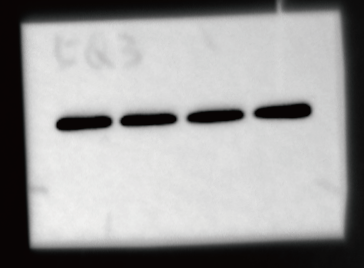

Supplement: Figure 5—source data 2. [file elife-99939-fig5-data2.zip › Figure 5-source data 2/Figure 5H input_Nur77.tif]

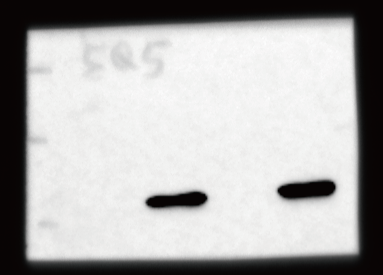

Supplement: Figure 5—source data 2. [file elife-99939-fig5-data2.zip › Figure 5-source data 2/Figure 5H input_VgrG2b-C.tif]

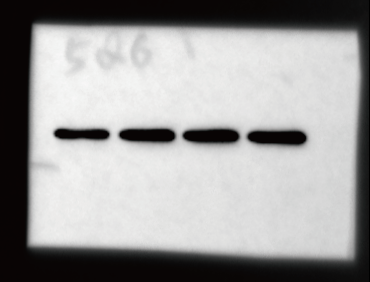

Supplement: Figure 5—source data 2. [file elife-99939-fig5-data2.zip › Figure 5-source data 2/Figure 5H input_β-actin.tif]

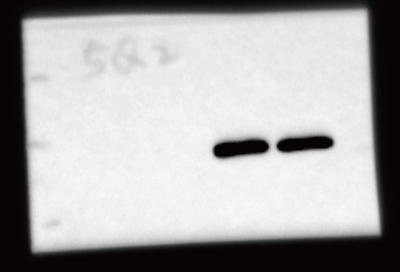

Supplement: Figure 5—source data 2. [file elife-99939-fig5-data2.zip › Figure 5-source data 2/Figure 5H IP_NLRP3.tif]

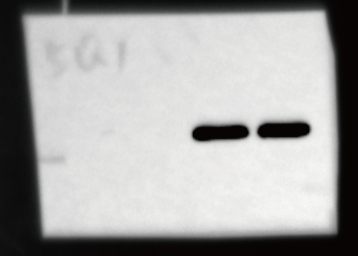

Supplement: Figure 5—source data 2. [file elife-99939-fig5-data2.zip › Figure 5-source data 2/Figure 5H IP_Nur77.tif]

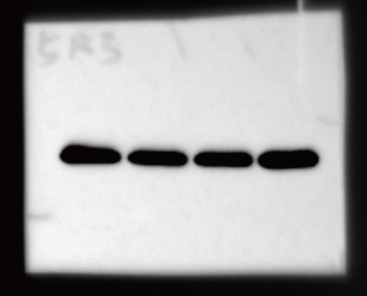

Supplement: Figure 5—source data 2. [file elife-99939-fig5-data2.zip › Figure 5-source data 2/Figure 5I input_NEK7.tif]

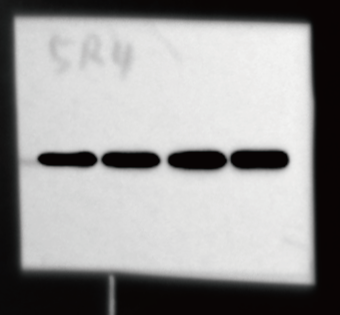

Supplement: Figure 5—source data 2. [file elife-99939-fig5-data2.zip › Figure 5-source data 2/Figure 5I input_NLRP3.tif]

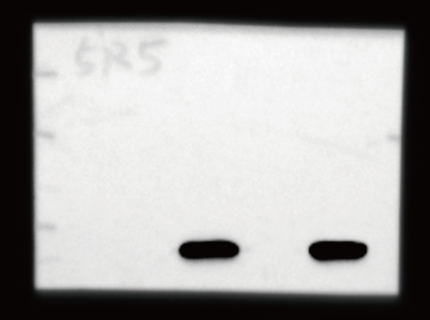

Supplement: Figure 5—source data 2. [file elife-99939-fig5-data2.zip › Figure 5-source data 2/Figure 5I input_VgrG2b-C.tif]

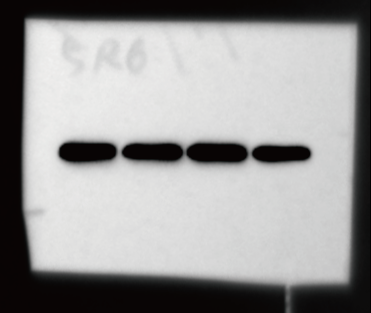

Supplement: Figure 5—source data 2. [file elife-99939-fig5-data2.zip › Figure 5-source data 2/Figure 5I input_β-actin.tif]

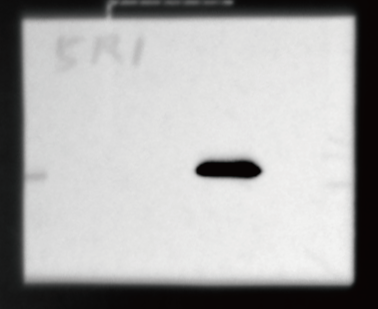

Supplement: Figure 5—source data 2. [file elife-99939-fig5-data2.zip › Figure 5-source data 2/Figure 5I IP_NEK7.tif]

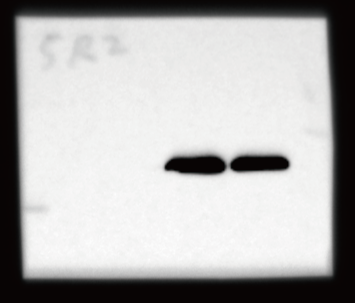

Supplement: Figure 5—source data 2. [file elife-99939-fig5-data2.zip › Figure 5-source data 2/Figure 5I IP_NLRP3.tif]

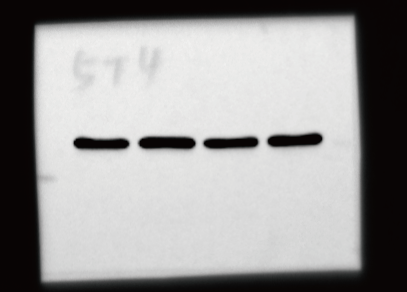

Supplement: Figure 5—source data 2. [file elife-99939-fig5-data2.zip › Figure 5-source data 2/Figure 5J input_NEK7.tif]

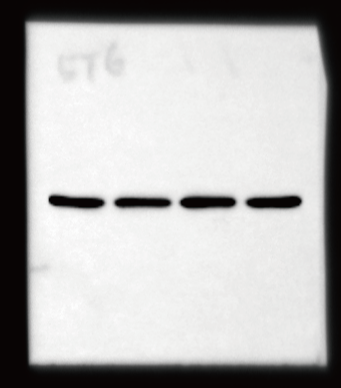

Supplement: Figure 5—source data 2. [file elife-99939-fig5-data2.zip › Figure 5-source data 2/Figure 5J input_NLRP3.tif]

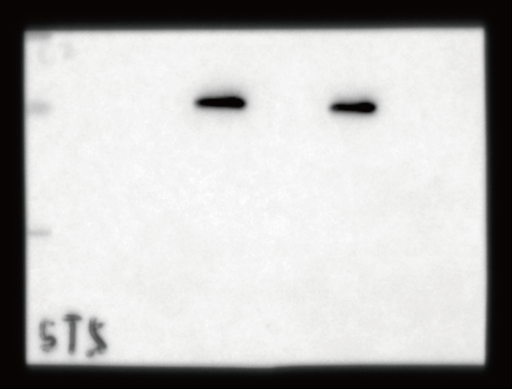

Supplement: Figure 5—source data 2. [file elife-99939-fig5-data2.zip › Figure 5-source data 2/Figure 5J input_VgrG2b-C.tif]

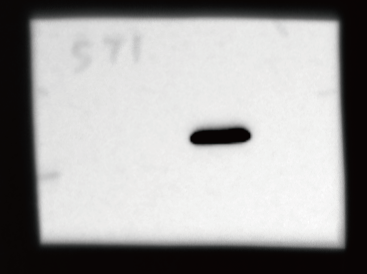

Supplement: Figure 5—source data 2. [file elife-99939-fig5-data2.zip › Figure 5-source data 2/Figure 5J IP_NEK7.tif]

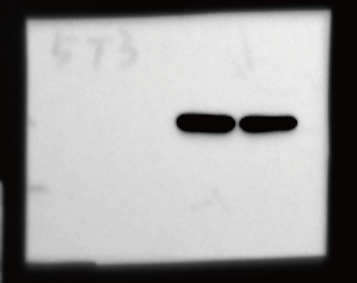

Supplement: Figure 5—source data 2. [file elife-99939-fig5-data2.zip › Figure 5-source data 2/Figure 5J IP_NLRP3.tif]

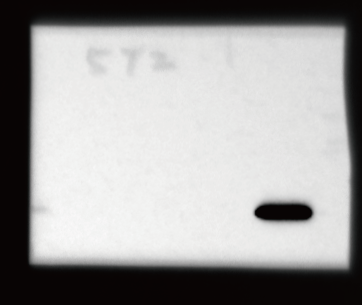

Supplement: Figure 5—source data 2. [file elife-99939-fig5-data2.zip › Figure 5-source data 2/Figure 5J IP_VgrG2b-C.tif]

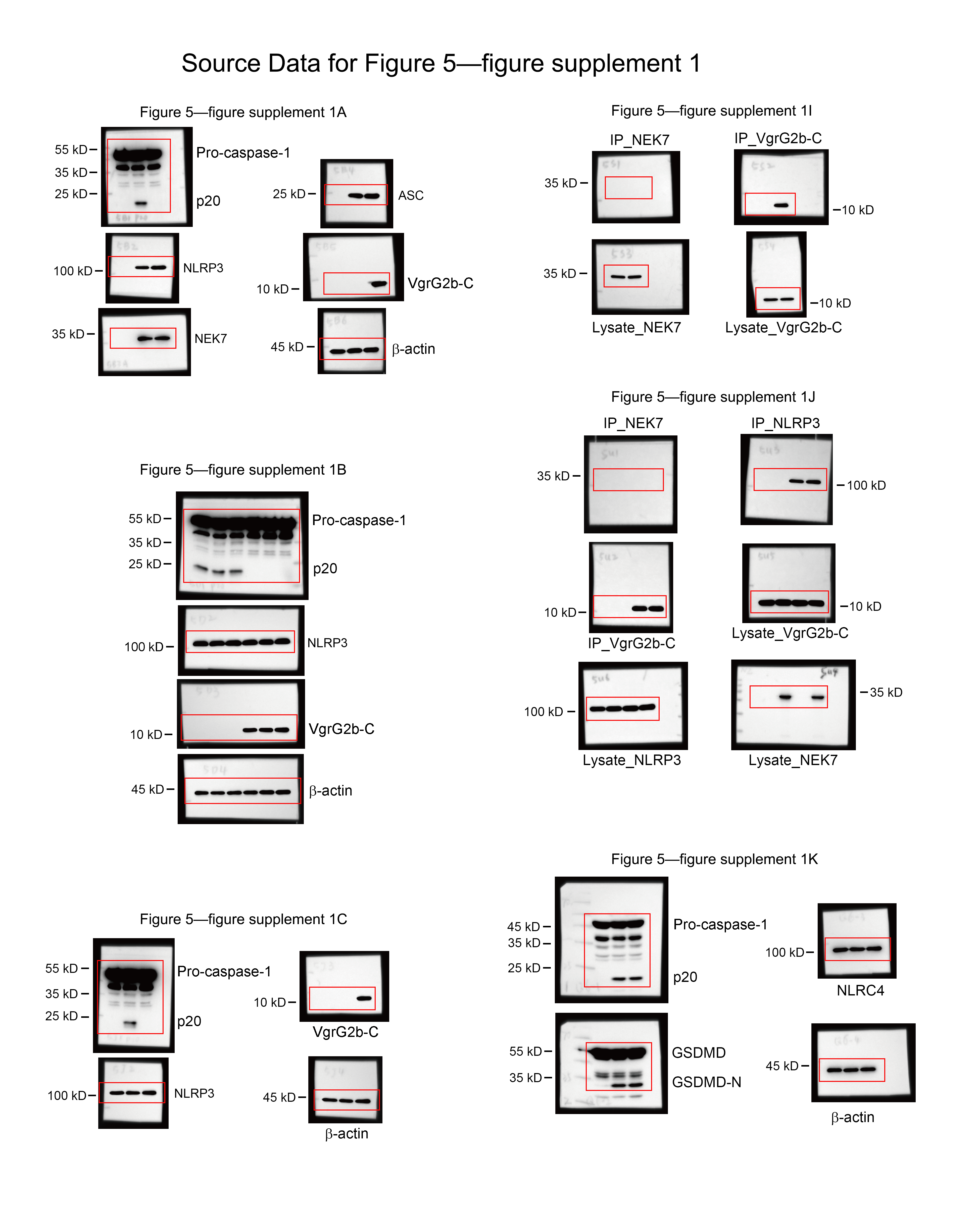

Supplement: Figure 5—figure supplement 1—source data 1. [file elife-99939-fig5-figsupp1-data1.zip › Figure 5-figure supplement 1.tif]

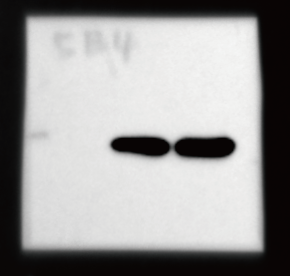

Supplement: Figure 5—figure supplement 1—source data 2. [file elife-99939-fig5-figsupp1-data2.zip › Figure 5-figure supplement 1-source data 2/Figure 5—figure supplement 1A ASC.tif]

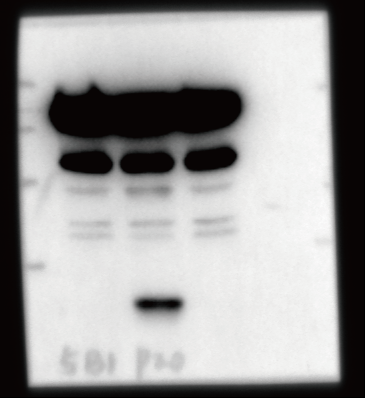

Supplement: Figure 5—figure supplement 1—source data 2. [file elife-99939-fig5-figsupp1-data2.zip › Figure 5-figure supplement 1-source data 2/Figure 5—figure supplement 1A caspase-1.tif]

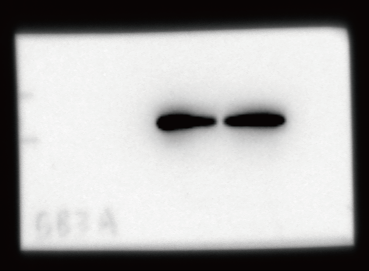

Supplement: Figure 5—figure supplement 1—source data 2. [file elife-99939-fig5-figsupp1-data2.zip › Figure 5-figure supplement 1-source data 2/Figure 5—figure supplement 1A NEK7.tif]

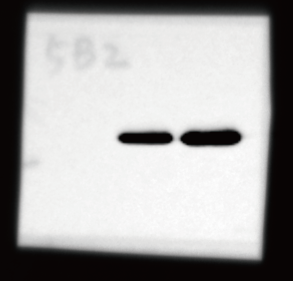

Supplement: Figure 5—figure supplement 1—source data 2. [file elife-99939-fig5-figsupp1-data2.zip › Figure 5-figure supplement 1-source data 2/Figure 5—figure supplement 1A NLRP3.tif]

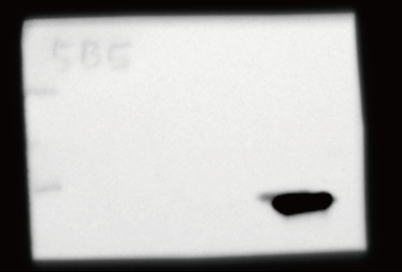

Supplement: Figure 5—figure supplement 1—source data 2. [file elife-99939-fig5-figsupp1-data2.zip › Figure 5-figure supplement 1-source data 2/Figure 5—figure supplement 1A VgrG2b-C.tif]

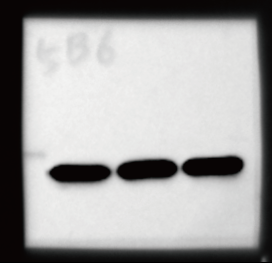

Supplement: Figure 5—figure supplement 1—source data 2. [file elife-99939-fig5-figsupp1-data2.zip › Figure 5-figure supplement 1-source data 2/Figure 5—figure supplement 1A β-actin.tif]

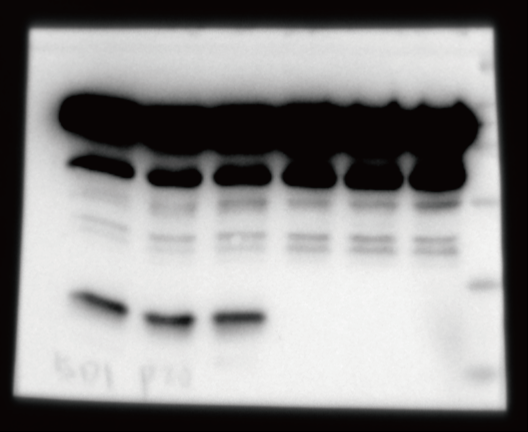

Supplement: Figure 5—figure supplement 1—source data 2. [file elife-99939-fig5-figsupp1-data2.zip › Figure 5-figure supplement 1-source data 2/Figure 5—figure supplement 1B caspase-1.tif]

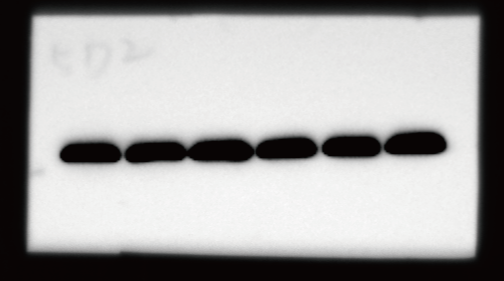

Supplement: Figure 5—figure supplement 1—source data 2. [file elife-99939-fig5-figsupp1-data2.zip › Figure 5-figure supplement 1-source data 2/Figure 5—figure supplement 1B NLRP3.tif]

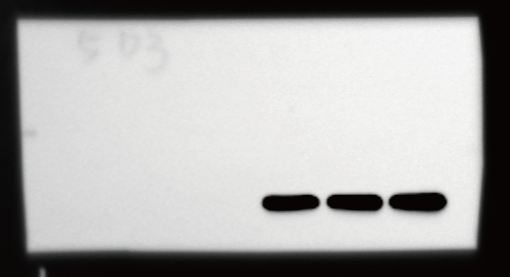

Supplement: Figure 5—figure supplement 1—source data 2. [file elife-99939-fig5-figsupp1-data2.zip › Figure 5-figure supplement 1-source data 2/Figure 5—figure supplement 1B VgrG2b-C.tif]

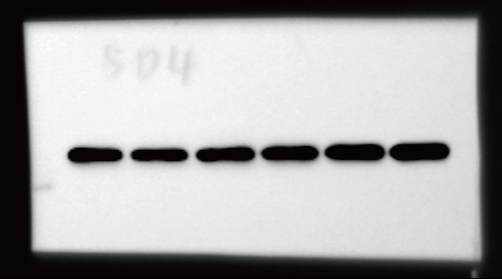

Supplement: Figure 5—figure supplement 1—source data 2. [file elife-99939-fig5-figsupp1-data2.zip › Figure 5-figure supplement 1-source data 2/Figure 5—figure supplement 1B β-actin.tif]
