# Supplementary figures and images for: A VgrG2b fragment cleaved by caspase-11/4 promotes Pseudomonas aeruginosa infection through suppressing the NLRP3 inflammasome (part 4 of 4)
Source: eLife. 2025 Feb 25;13:RP99939. doi: 10.7554/eLife.99939 (PMC11856931; doi:10.7554/eLife.99939)

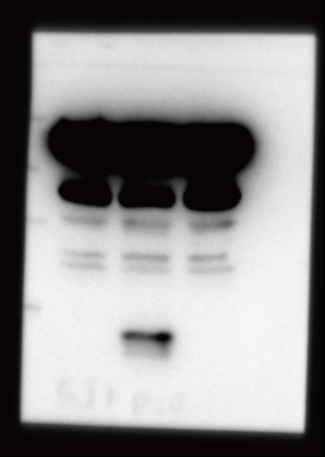

Supplement: Figure 5—figure supplement 1—source data 2. [file elife-99939-fig5-figsupp1-data2.zip › Figure 5-figure supplement 1-source data 2/Figure 5—figure supplement 1C caspase-1.tif]

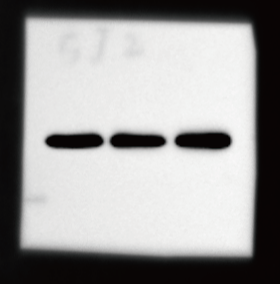

Supplement: Figure 5—figure supplement 1—source data 2. [file elife-99939-fig5-figsupp1-data2.zip › Figure 5-figure supplement 1-source data 2/Figure 5—figure supplement 1C NLRP3.tif]

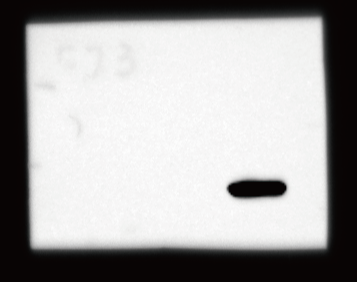

Supplement: Figure 5—figure supplement 1—source data 2. [file elife-99939-fig5-figsupp1-data2.zip › Figure 5-figure supplement 1-source data 2/Figure 5—figure supplement 1C VgrG2b-C.tif]

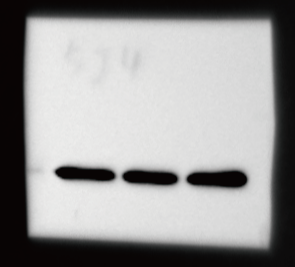

Supplement: Figure 5—figure supplement 1—source data 2. [file elife-99939-fig5-figsupp1-data2.zip › Figure 5-figure supplement 1-source data 2/Figure 5—figure supplement 1C β-actin.tif]

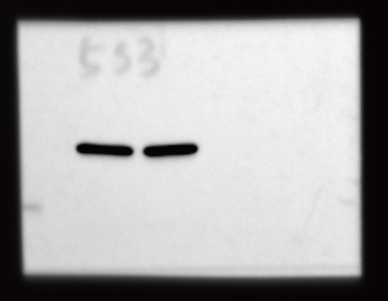

Supplement: Figure 5—figure supplement 1—source data 2. [file elife-99939-fig5-figsupp1-data2.zip › Figure 5-figure supplement 1-source data 2/Figure 5—figure supplement 1I input_NEK7.tif]

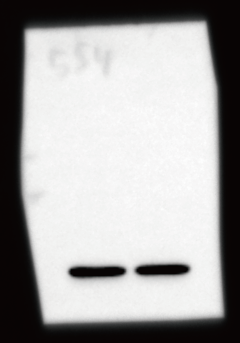

Supplement: Figure 5—figure supplement 1—source data 2. [file elife-99939-fig5-figsupp1-data2.zip › Figure 5-figure supplement 1-source data 2/Figure 5—figure supplement 1I input_VgrG2b-C.tif]

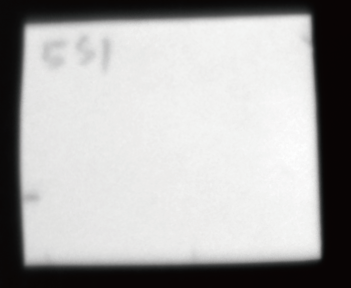

Supplement: Figure 5—figure supplement 1—source data 2. [file elife-99939-fig5-figsupp1-data2.zip › Figure 5-figure supplement 1-source data 2/Figure 5—figure supplement 1I IP_NEK7.tif]

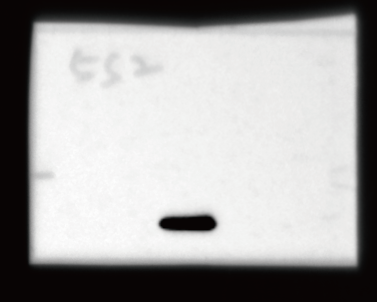

Supplement: Figure 5—figure supplement 1—source data 2. [file elife-99939-fig5-figsupp1-data2.zip › Figure 5-figure supplement 1-source data 2/Figure 5—figure supplement 1I IP_VgrG2b-C.tif]

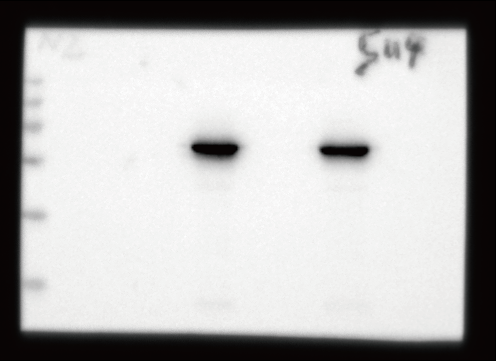

Supplement: Figure 5—figure supplement 1—source data 2. [file elife-99939-fig5-figsupp1-data2.zip › Figure 5-figure supplement 1-source data 2/Figure 5—figure supplement 1J input_NEK7.tif]

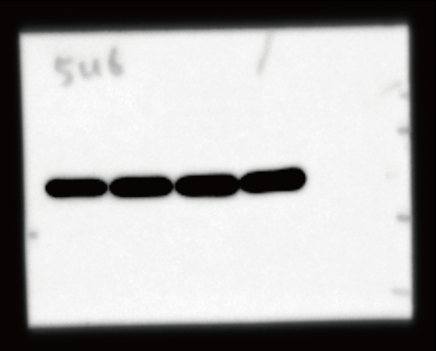

Supplement: Figure 5—figure supplement 1—source data 2. [file elife-99939-fig5-figsupp1-data2.zip › Figure 5-figure supplement 1-source data 2/Figure 5—figure supplement 1J input_NLRP3.tif]

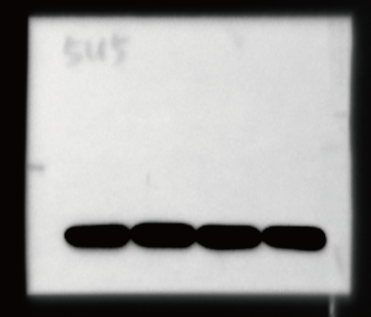

Supplement: Figure 5—figure supplement 1—source data 2. [file elife-99939-fig5-figsupp1-data2.zip › Figure 5-figure supplement 1-source data 2/Figure 5—figure supplement 1J input_VgrG2b-C.tif]

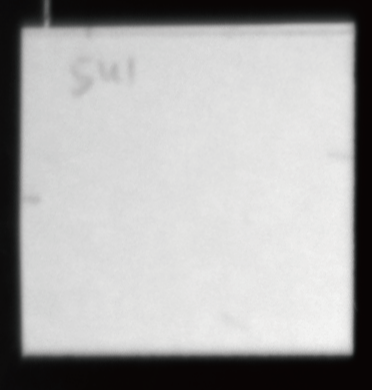

Supplement: Figure 5—figure supplement 1—source data 2. [file elife-99939-fig5-figsupp1-data2.zip › Figure 5-figure supplement 1-source data 2/Figure 5—figure supplement 1J IP_NEK7.tif]

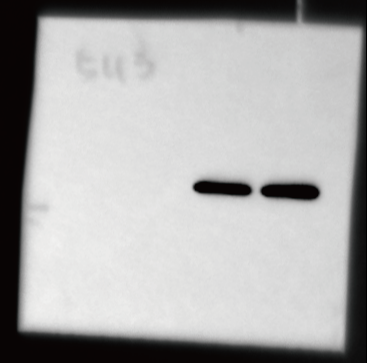

Supplement: Figure 5—figure supplement 1—source data 2. [file elife-99939-fig5-figsupp1-data2.zip › Figure 5-figure supplement 1-source data 2/Figure 5—figure supplement 1J IP_NLRP3.tif]

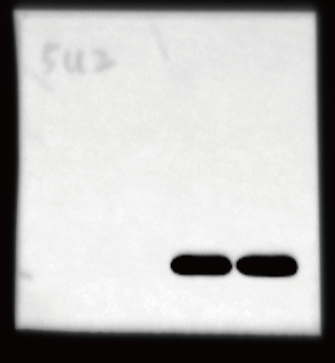

Supplement: Figure 5—figure supplement 1—source data 2. [file elife-99939-fig5-figsupp1-data2.zip › Figure 5-figure supplement 1-source data 2/Figure 5—figure supplement 1J IP_VgrG2b-C.tif]

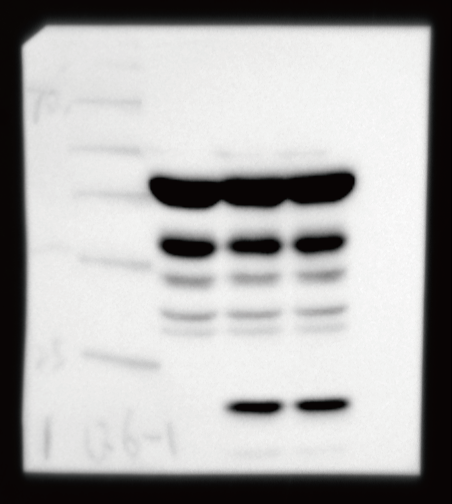

Supplement: Figure 5—figure supplement 1—source data 2. [file elife-99939-fig5-figsupp1-data2.zip › Figure 5-figure supplement 1-source data 2/Figure 5—figure supplement 1K caspase-1.tif]

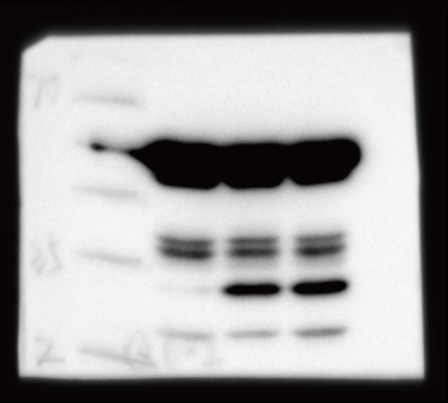

Supplement: Figure 5—figure supplement 1—source data 2. [file elife-99939-fig5-figsupp1-data2.zip › Figure 5-figure supplement 1-source data 2/Figure 5—figure supplement 1K GSDMD.tif]

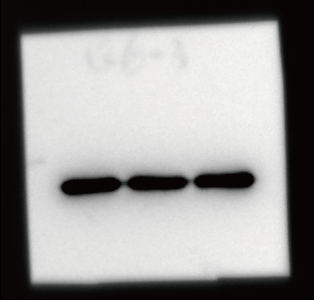

Supplement: Figure 5—figure supplement 1—source data 2. [file elife-99939-fig5-figsupp1-data2.zip › Figure 5-figure supplement 1-source data 2/Figure 5—figure supplement 1K NLRC4.tif]

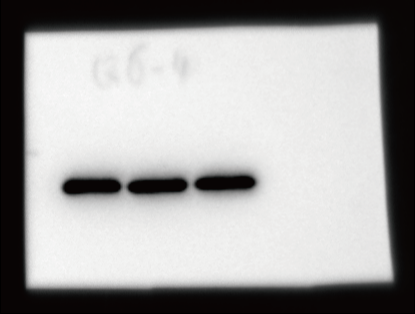

Supplement: Figure 5—figure supplement 1—source data 2. [file elife-99939-fig5-figsupp1-data2.zip › Figure 5-figure supplement 1-source data 2/Figure 5—figure supplement 1K β-actin.tif]

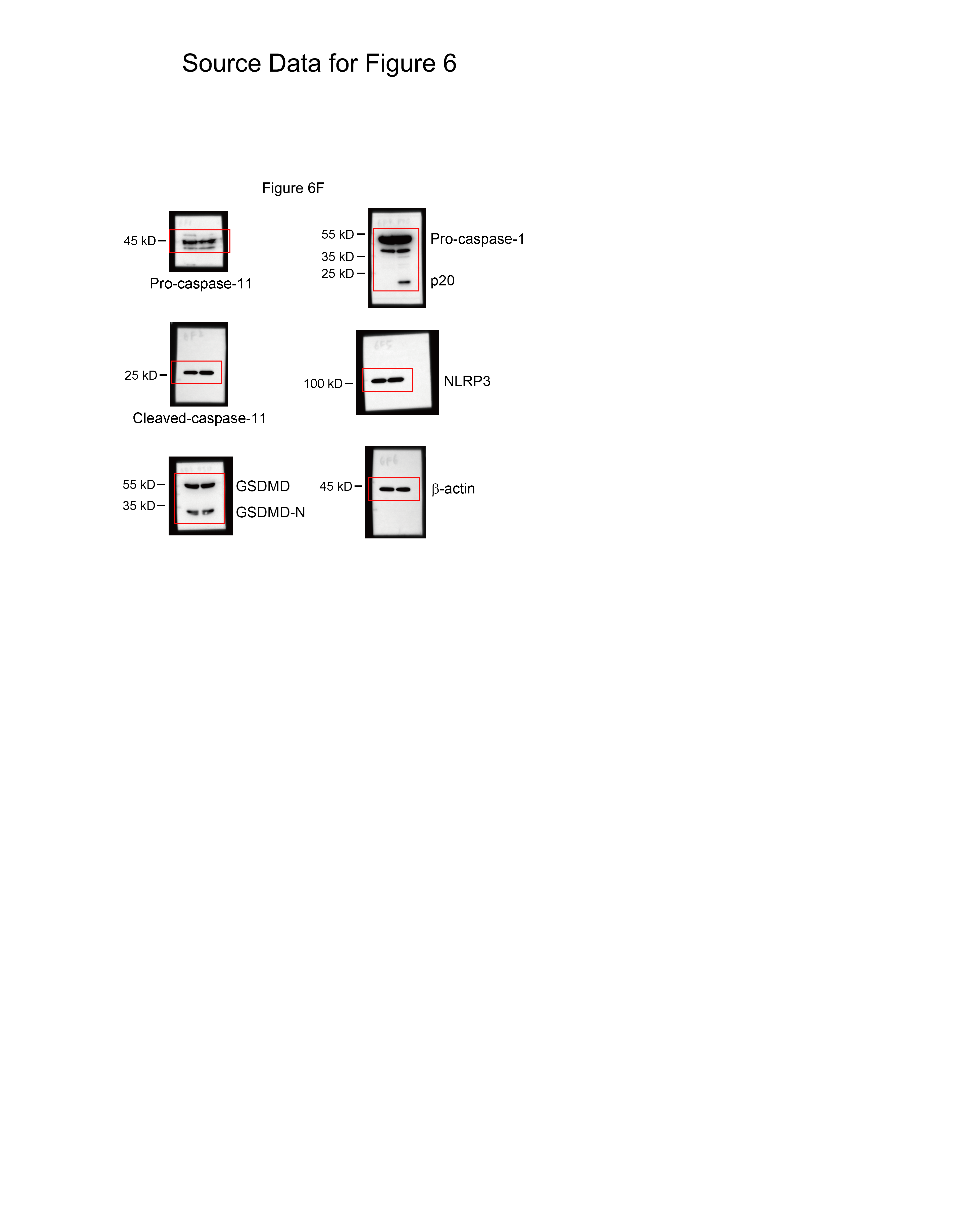

Supplement: Figure 6—source data 1. [file elife-99939-fig6-data1.zip › Figure 6-source data 1.tif]

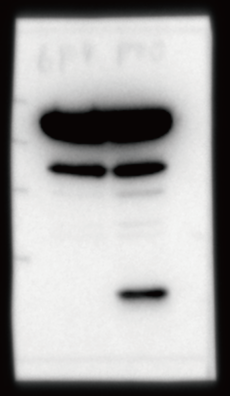

Supplement: Figure 6—source data 2. [file elife-99939-fig6-data2.zip › Figure 6-source data 2/Figure 6F caspase-1.tif]

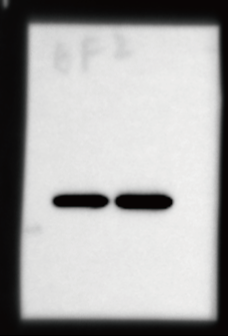

Supplement: Figure 6—source data 2. [file elife-99939-fig6-data2.zip › Figure 6-source data 2/Figure 6F cleaved-caspase-11.tif]

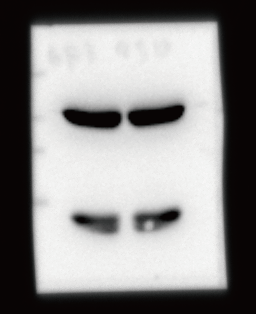

Supplement: Figure 6—source data 2. [file elife-99939-fig6-data2.zip › Figure 6-source data 2/Figure 6F GSDMD.tif]

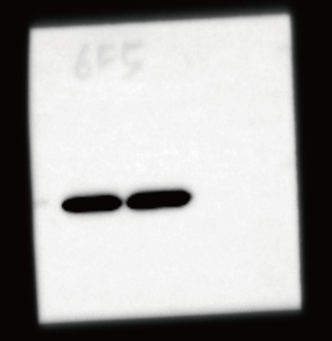

Supplement: Figure 6—source data 2. [file elife-99939-fig6-data2.zip › Figure 6-source data 2/Figure 6F NLRP3.tif]

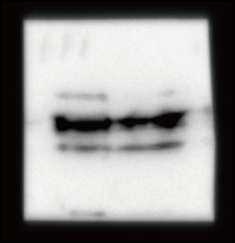

Supplement: Figure 6—source data 2. [file elife-99939-fig6-data2.zip › Figure 6-source data 2/Figure 6F pro-caspase-11.tif]

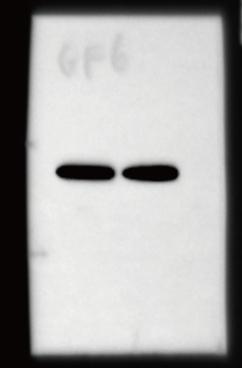

Supplement: Figure 6—source data 2. [file elife-99939-fig6-data2.zip › Figure 6-source data 2/Figure 6F β-actin.tif]

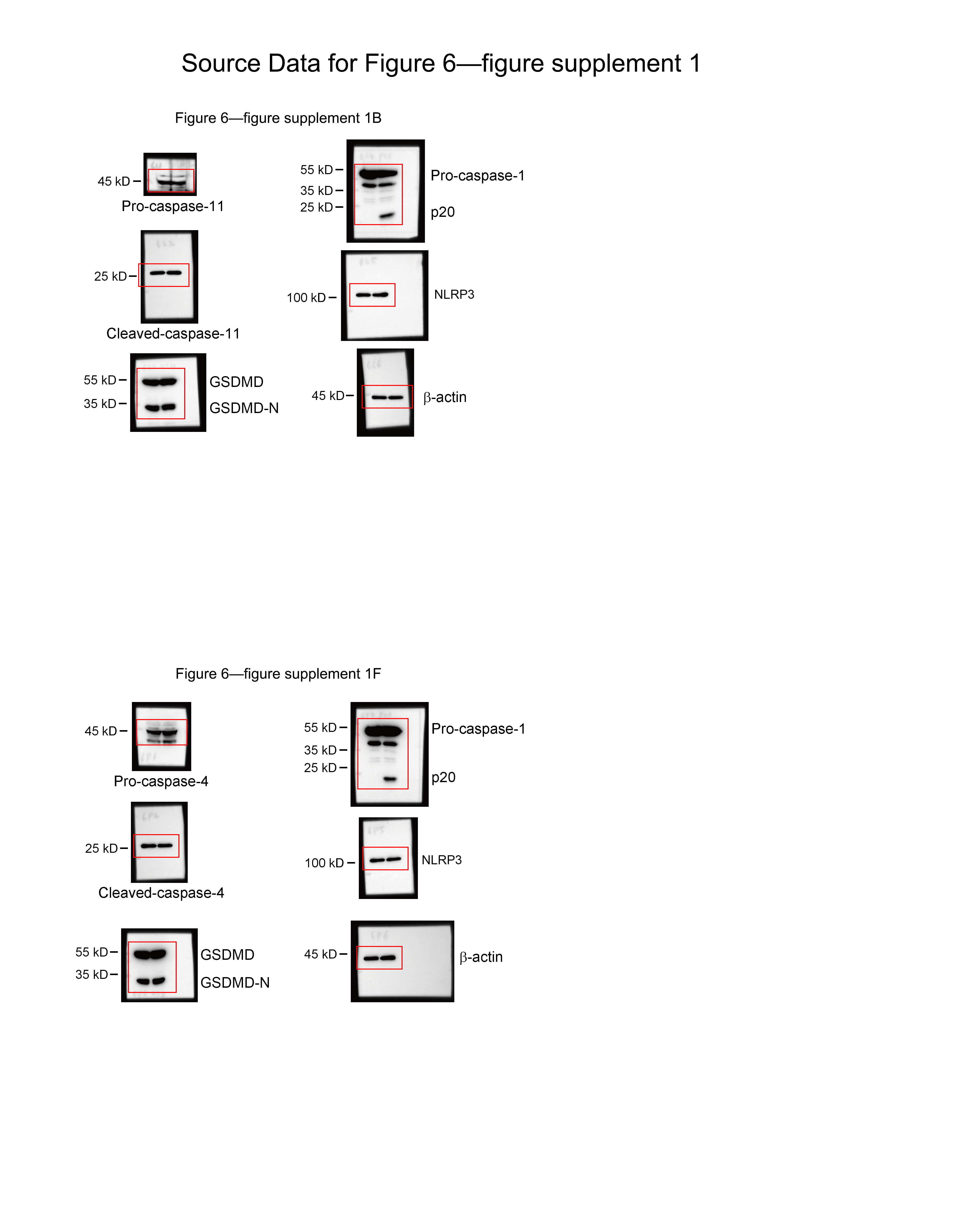

Supplement: Figure 6—figure supplement 1—source data 1. [file elife-99939-fig6-figsupp1-data1.zip › Figure 6-figure supplement 1.tif]

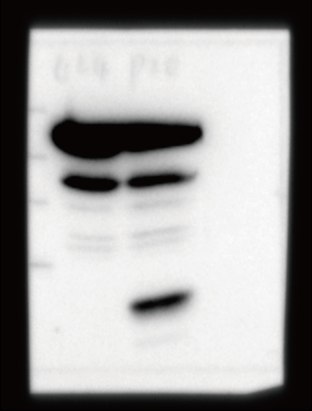

Supplement: Figure 6—figure supplement 1—source data 2. [file elife-99939-fig6-figsupp1-data2.zip › Figure 6-figure supplement 1-source data 2/Figure 6—figure supplement 1B caspase-1.tif]

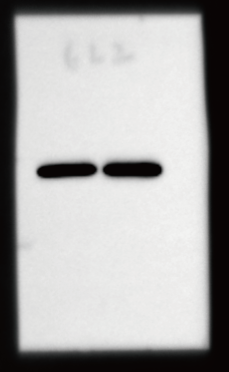

Supplement: Figure 6—figure supplement 1—source data 2. [file elife-99939-fig6-figsupp1-data2.zip › Figure 6-figure supplement 1-source data 2/Figure 6—figure supplement 1B cleaved-caspase-11.tif]

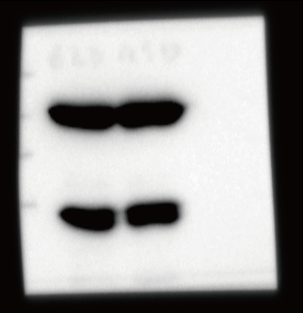

Supplement: Figure 6—figure supplement 1—source data 2. [file elife-99939-fig6-figsupp1-data2.zip › Figure 6-figure supplement 1-source data 2/Figure 6—figure supplement 1B GSDMD.tif]

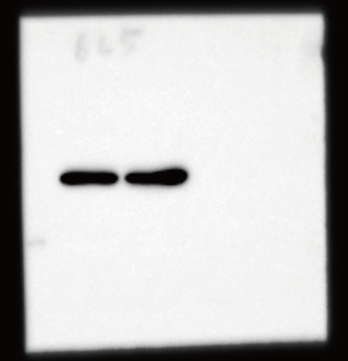

Supplement: Figure 6—figure supplement 1—source data 2. [file elife-99939-fig6-figsupp1-data2.zip › Figure 6-figure supplement 1-source data 2/Figure 6—figure supplement 1B NLRP3.tif]

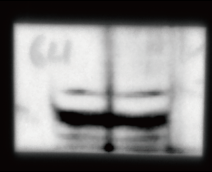

Supplement: Figure 6—figure supplement 1—source data 2. [file elife-99939-fig6-figsupp1-data2.zip › Figure 6-figure supplement 1-source data 2/Figure 6—figure supplement 1B pro-caspase-11.tif]

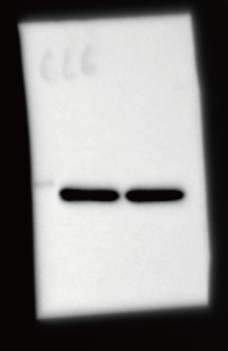

Supplement: Figure 6—figure supplement 1—source data 2. [file elife-99939-fig6-figsupp1-data2.zip › Figure 6-figure supplement 1-source data 2/Figure 6—figure supplement 1B β-actin.tif]

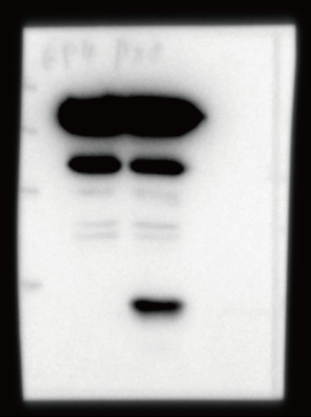

Supplement: Figure 6—figure supplement 1—source data 2. [file elife-99939-fig6-figsupp1-data2.zip › Figure 6-figure supplement 1-source data 2/Figure 6—figure supplement 1F caspase-1.tif]

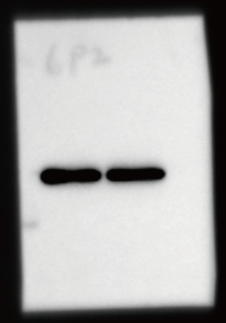

Supplement: Figure 6—figure supplement 1—source data 2. [file elife-99939-fig6-figsupp1-data2.zip › Figure 6-figure supplement 1-source data 2/Figure 6—figure supplement 1F cleaved-caspase-4.tif]

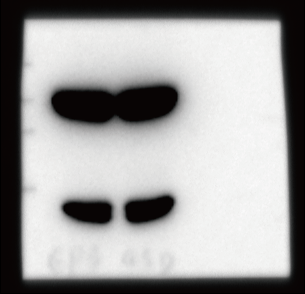

Supplement: Figure 6—figure supplement 1—source data 2. [file elife-99939-fig6-figsupp1-data2.zip › Figure 6-figure supplement 1-source data 2/Figure 6—figure supplement 1F GSDMD.tif]

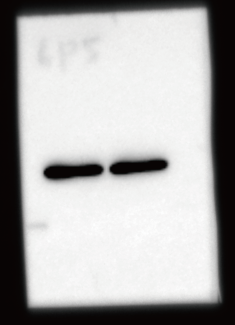

Supplement: Figure 6—figure supplement 1—source data 2. [file elife-99939-fig6-figsupp1-data2.zip › Figure 6-figure supplement 1-source data 2/Figure 6—figure supplement 1F NLRP3.tif]

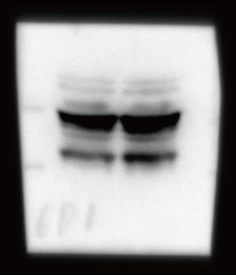

Supplement: Figure 6—figure supplement 1—source data 2. [file elife-99939-fig6-figsupp1-data2.zip › Figure 6-figure supplement 1-source data 2/Figure 6—figure supplement 1F pro-caspase-4.tif]

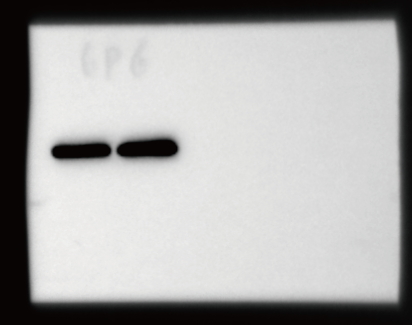

Supplement: Figure 6—figure supplement 1—source data 2. [file elife-99939-fig6-figsupp1-data2.zip › Figure 6-figure supplement 1-source data 2/Figure 6—figure supplement 1F β-actin.tif]
