# Supplementary material for: A VgrG2b fragment cleaved by caspase-11/4 promotes Pseudomonas aeruginosa infection through suppressing the NLRP3 inflammasome
Source: eLife. 2025 Feb 25;13:RP99939. doi: 10.7554/eLife.99939 (PMC11856931; doi:10.7554/eLife.99939)
Supplement: Peer review history

# Editor report: eLife Assessment

**DOI:** 10.7554/eLife.99939.3.sa0

**Authors:** Carla V Rothlin

**Affiliations:** Yale University United States

**Keywords:** Solid, Valuable

This **valuable** study on Pseudomonas subverting host immunity identifies a new immune evasion strategy. There is **solid** evidence for the cleavage of VgrG2B by Caspase 11 and the generation of fragments that inhibit activity of the NLRP3 inflammasome. This work should be of interest to immunologists and microbiologists.

# Referee report: Reviewer #2 (Public review)

**DOI:** 10.7554/eLife.99939.3.sa1

Summary:

In their manuscript, Quian and colleagues identified a novel mechanisms by which Pseudomonas control inflammatory responses upon inflammasome activation. They identified a caspase-11 substrates (VgrG2b) which, upon cleavage, binds and inhibit the NLRP3 to reduce the production of pro-inflammatory cytokines. This is a unique mechanism that allow for the tailoring of the innate immune response upon bacterial recognition.

Strengths:

The authors are presenting here a novel conceptual framework in host-pathogen interactions. Their work is supported by a range of approaches (biochemical, cellular immunology, microbiology, animal models) and their conclusions are supported by multiple independent evidences. The work is likely to have an important impact in the innate immunity field and host-pathogen interactions field and may guide the development of novel inhibitors.

Weaknesses:

Although quite exhaustive, a few of the authors conclusions are not fully supported (e.g, caspase-11 directly cleaving VgrG2b, the unique affinity of VgrG2b-C for NLRP3) and would require complementary approaches to validate their findings fully. This is minimal.

Comments on revisions:

I command the authors's effort to address my comments. They have addressed all my concerns.

# Author comment: Author response

**DOI:** 10.7554/eLife.99939.3.sa2

**Authors:** Yan Qian, Qiannv Liu, Xiangyun Cheng, Chunlei Wang, Chun Kong, Mengqian Li, Chao Ren, Dong Jiang, Shuo Wang, Pengyan Xia

**Affiliations:** Peking University Beijing China; Peking University beijing China; Peking University Third Hospital beijing China; Peking University Beijing China; Peking University beijing China; Peking University beijing China; Beijing Chao-Yang Hospital beijing China; Peking University Third Hospital beijing China; Institute of Microbiology, Chinese Academy of Sciences. Beijing China; Peking University Beijing China

The following is the authors’ response to the original reviews.

**Reviewer #1 (Public review):**

In the manuscript entitled "A VgrG2b fragment cleaved by caspase-11/4 promotes *Pseudomonas aeruginosa* infection through suppressing the NLRP3 inflammasome", Qian et al. found an activation of the non-canonical inflammasome, but not the downstream NLRP3 inflammasome, during the infection of macrophage by P. aeruginosa, which is in sharp contrast to that by *E. coli* (Figure 1). In realizing that the suppression of the NLRP3 inflammasome is Caspase-11 dependent, the authors performed a screening among P. aeruginosa proteins and identified VgrG2b being a major substrate of Caspase-11 (Figure 2). Next, the authors mapped the cleavage site on VgrG2b to D883, and demonstrated that cleavage of VgrG2b by Caspase-11 is essential for the suppression of the NLRP3 inflammasome (Figure 3). Furthermore, they found that a binding between the C-terminal fragment of the cleaved VgrG2b and NLRP3 existed (Figure 4), which was then proved to block the association of NLRP3 with NEK7 (Figure 5). Finally, the authors demonstrated that blocking of VgrG2b cleavage, by either mutation of the D883 or administration of a designed peptide, effectively improved the survival rate of the P. aeruginosa-infected mice (Figure 6). This is a well-designed and executed study, with the results clearly presented and stated.

We are deeply grateful for your recognition and positive comments on our article. Thank you for your effort and dedication in reviewing our manuscript. We are honored to have the opportunity to receive feedback form professional reviewers like you.

**Reviewer #2 (Public review):**

Summary:

In their manuscript, Quian and colleagues identified a novel mechanism by which Pseudomonas control inflammatory responses upon inflammasome activation. They identified a caspase-11 substrate (VgrG2b) which, upon cleavage, binds and inhibits the NLRP3 to reduce the production of pro-inflammatory cytokines. This is a unique mechanism that allows for the tailoring of the innate immune response upon bacterial recognition.

Strengths:

The authors are presenting here a novel conceptual framework in host-pathogen interactions. Their work is supported by a range of approaches (biochemical, cellular immunology, microbiology, animal models), and their conclusions are supported by multiple independent evidences. The work is likely to have an important impact on the innate immunity field and host-pathogen interactions field and may guide the development of novel inhibitors.

Weaknesses:

Although quite exhaustive, a few of the authors' conclusions are not fully supported (e.g., caspase-11 directly cleaving VgrG2b, the unique affinity of VgrG2b-C for NLRP3) and would require complementary approaches to validate their findings fully. This is minimal.

We sincerely appreciate your professional review and kind appraisal on our article. These comments are really valuable and helpful for improving our manuscript. According to your suggestions, we have made some modifications and added some supplemental data to make our results more convincing. The detailed responses are listed point-by-point below.

**Recommendations for the authors:**

**Reviewer #2 (Recommendations for the authors):**

I really enjoyed reading your manuscript and believe this is an important conceptual advance for the innate immunity field. Your conclusions are in general well-supported, you used a range of methodologies and the quality of the presentation of the results is excellent. I have a few comments here that I hope will contribute to improving an already great piece of work:

Elements to be improved:

Line 109-110: the author claims that the release of mito DNA is required for NLRP3 activation. ' I would support this with a reference. I believe this may not be fully agreed on in the field. Cleavage of GSDMD by caspase4/11 is required, however. A few groups showed the required for K+ efflux in this context (Broz, Brough, Schroder labs).

It is a very good suggestion. Indeed, there is still controversy over this issue, and we have revised our text to make our manuscript more neutral. We have also cited these important references to help readers understand where the controversy lies.

I disagree that OMV _+ Pseudomonas is a natural way to simulate natural infection. I would argue it is even quite artificial. Pseudomonas alone should be sufficient to generate OMV without the addition of extra OMVs.

This is a good point. Before we infected BMDM cells with PAO1 stains, we had washed with PBS for at least three times to exclude the interference of contents in the LB medium. Moreover, in our experimental system, the time for co-incubation between bacteria and host cells is very limited. During this time, the amount of OMV secreted by bacteria may not reach the level of activating inflammasomes, and this concentration is also relatively low compared to the OMV concentration secreted by bacteria under physiological conditions. Therefore, we added extra OMVs to simulate the chronic infection condition in a short time.

The co-expression of caspase with VrG2b and assume the cleavage is direct. However, the work is lacking work with recombinant proteases (commercially available), which would strengthen their conclusions regarding the ability of caspase-4/11 to directly cleave the protein. Based on the recognised sequence (DXXD), I believe caspase-4/11 is not directly responsible for this. These caspases were shown to cleave caspase-3/7, which can cleave such sequence (DXXX). As caspase-4 can cleave caspase-3/7 in their lysates, I would recommend testing this hypothesis to further strengthen the authors' conclusions.

These are very good points. As data shown on Fig. 3F, we used recombinant VgrG2b and caspase-11 p22/p10 to prove the direct cleavage of caspase-11. To exclude the effect of caspase-3/7, we treated cells with inhibitors of caspase-3/7 and found that caspase-3/7 are not the executor for VgrG2b cleavage (new Figure 3—figure supplement 1E, F).

The affinity between caspase-11 and VgrG2b-C is puzzling as one would normally expect the caspase and its substrates to quickly dissociate. Does VgrG2b-C impact the activity of caspase-4/11 upon cleavage? Can VrgG2b-C also interact with p20/p10 caspase-1? I believe the authors only tried the full-length version of caspase-1 in supplemental.

These are very good questions. We agree enzymes and substrates only have temporary interactions normally, which are not easy to catch. However, we used mutant caspase-11(C254A) inhibiting its cleavage of substrates, so that the combination of VgrG2b or VgrG2b-C with caspase-11(C254A) could be detected. This mutation is frequently used in immunoprecipitation (Wang K, Cell, 2020). We had tested the impact of VgrG2b-C on the enzyme activity of caspase-4/11, and showed that VgrG2b-C did not affect the cleavage of GSDMD by caspase-11 (Figure 5C). We also tried the caspase-1 p20/p10, also found that they had no interaction with VgrG2b-C (new Figure 4—figure supplement 1G).

Can more details be provided about the generation of recombinant caspase-11, VgrG2b-C, and other recombinant proteins tested?

Thanks for your suggestion, we have revised our description in the new version.

The authors assumed that VgrG2C-b does not impact other inflammasome (such as NLRC4) based on their X-gal assay. I would also confirm this with a functional assay (e.g., transfection of flagellin in macrophages).

This is a good suggestion. We have tested the impact of VgrG2b-C on NLRC4 inflammasome and found that VgrG2b-C does not affect NLRC4 activation with the transfection of flagellin (new Figure 5—figure supplement 1K).

Often, representative experiments are shown. For Elisa, cell death assays and quantitative experiments, pooling the data would be appropriate. Appropriate statistical analysis should be conducted based on this as well.

Thanks for your suggestions. In the revised manuscript, we pooled the data of three independent experiments for our analysis of ELISA and cell death assays. We also added descriptions of statistical analysis in our revised text.

VgrG2b has been suggested to be a metalloprotease (PMID: 31577948). Is its protease activity required for the phenomenon observed?

This is a very good question. The active region of metalloprotease VgrG2b-C is aa932-941, especially the core sequence of HEXXH. Structure data also confirms that H935, E936, H939, E983 play key roles in the coordination with Zn ions (Sana TG, mBio, 2015; Wood TE, Cell reports, 2019). In our study, the cleavage of VgrG2b by caspase-4/11 depends on the recognition of tetrapeptide sequence in aa880-883. We added data showing that the cleavage of VgrG2b and the inhibition of NLRP3 inflammasome were not affected by VgrG2b enzymatic activity (new Figure 4—figure supplement 1I-K).

What is the affinity of VgrG2b-C for NLRP3? Is it higher than NEK7? A quantitative experiment would be required to claim this.

This is a great point of view. We added the quantitative data certifying that VgrG2b-C has higher affinity with NLRP3 compared with NEK7 in the revised manuscript (326 nM VS 681 nM).

The Material and Method section is a bit light and would benefit from adding more information (e.g. cell density, microscopy details, number of cells imaged, etc).

Thanks for your suggestion. We have added more details in the Material and Method section in revised manuscript.
